# Supplementary material for: How to implement a clinical ethics committee in an oncological research hospital: Qualitative results from a process evaluation study using normalization process theory (EVACEC)
Source: PLoS One. 2025 May 6;20(5):e0318870. doi: 10.1371/journal.pone.0318870 (PMC12054913; doi:10.1371/journal.pone.0318870)
Supplement: S1 File — NPT core concepts: this file contains the description of the concepts of the Normalisation Process Theory (NPT), the framework we used to inform both the interview guide and the analysis of our data. (DOCX) [file pone.0318870.s001.docx]

*Core NPT concepts and related sub-concepts, downloaded from the NPT online toolkit^2^*

| **CONCEPT** | **KEY ATTRIBUTE** | **WORKING DEFINITION** |
| --- | --- | --- |
| **Coherence**  *Sense-making*  The extent to which individuals understand all the elements of the intervention and the reasons for adopting a new intervention | Differentiation | Whether the intervention is easy to describe to participants and whether they can approach how it differs or is clearly distinct from current ways of working |
|  | Communal specification | Whether participants have or are able to build a shared understanding of the aims, objectives, and expected outcomes of the proposed intervention |
|  | Individual specification | Whether individual participants have or are able to make sense of the work-specific tasks and responsibilities – the proposed intervention would create for |
|  | Internalization | Whether participants have or are able to easily grasp the potential value, benefits and importance of the intervention |
| **Cognitive participation**  *Engagement*  The extent to which individuals believe in the innovation provided by the intervention and start to prepare for it | Initiation | Whether or not key individuals are able and willing to get others involved in the new practice |
|  | Enrolment | Whether or not participants believe it is right for them to be involved, and that they can make a contribution to the implementation work |
|  | Legitimation | The capacity and willingness of participants to organize themselves in order to collectively contribute to the work involved in the new practice |
|  | Active action | The capacity and willingness of participants to collectively define the actions and procedures needed to keep new practice going |
| **Collective action**  *Enacting*  What happens when the intervention is operationalized | Interactional workability | Whether people are able to enact the intervention and operationalize its components in practice |
|  | Relational Integration | Whether people maintain trust in the intervention and in each other |
|  | Skill Set workability | Whether the work required by the intervention is seen to be parceled out to participants with the right mix of skills and training to do it |
|  | Contextual integration | Whether the intervention is supported by management and other stakeholders, policy, money and material resources. |
| **Reflexive monitoring**  *Appraisal*  The act of keeping an innovation under review and of adapting it intelligently to changing circumstances | Systematization | Whether participants can determine how effective and useful the intervention is from the use of formal and/or informal evaluation methods |
|  | Communal appraisal | Whether, as a result of formal monitoring, participants collectively agree about the worth of the effects of the intervention |
|  | Individual appraisal | Whether individuals involved with, or affected by the intervention, think it is worthwhile |
|  | Reconfiguration | Whether individuals or groups using the intervention can make changes as a result of individual and communal appraisal |
