# Supplementary material for: How to implement a clinical ethics committee in an oncological research hospital: Qualitative results from a process evaluation study using normalization process theory (EVACEC)
Source: PLoS One. 2025 May 6;20(5):e0318870. doi: 10.1371/journal.pone.0318870 (PMC12054913; doi:10.1371/journal.pone.0318870)
Supplement: S3 File — interview topic guides. (DOCX) [file pone.0318870.s003.docx]

# Supplementary Material - Interviews Topic Guide

Table 1: outline of NPT framework topics and their application to CEC implementation

| **Coherence** | What is the process? | How participants involved in its implementation perceive the CEC and whether they experienced the CEC as valuable and agreed about its usefulness and purpose. |
| --- | --- | --- |
| **Cognitive participation** | Who performs the process? | Whether participants saw the CEC as a legitimate part of their work and whether they supported it over time |
| **Collective action** | How is the process performed? | How was the CEC built within the existing context, how the embedding and integration had proceeded, and what factors promoted or inhibited the work |
| **Reflexive monitoring** | How is the process understood? | How participants individually and collectively evaluate the CEC |

## **Local health Directors (LD)**

### **Themes to be explored:**

1. Perception of the role of the CEC in the local context and reasons for implementing the CEC.
2. Understanding of its own activity in support of the CEC.
3. Understanding of strengths and weaknesses related to the service.
4. Reflection on service utilization and perception of impact in the local context

### **Interview guide:**

#### **Opening**

- Good morning, thank you for your time to participate in this study. Regarding the purpose of the research, do you have any questions or concerns that you would like to clarify?
- Would you like to tell me about your experience with the CEC?

#### **Theme A – coherence:**

- In your opinion, what is the role of the CEC within the Local Health Authority of Reggio Emilia?
- How, in your opinion, it is different from other services and what is its specificity?
- What is the goal of the CEC and how does it act in the local context?
- Are there any motivations that led you to support/promote this service?

#### **Theme B – cognitive participation**

- How do you help promoting this service?
- What do you think about the value of a service like the CEC to your work and to your colleagues’?

#### **Theme C – collective action**

- Thinking about the activities carried out by the CEC, how have they been integrated into the local context?
- Are there any limitations - organizational/internal/external to the service/economic? – that you perceive with respect to the CEC? If so, which ones?
- Are there any potentialities with respect to the service that has been implemented? If yes, which ones?

#### **Theme D – reflexive monitoring**

- In light of what has been done in these 16 months, how do you evaluate the implemented service?
- In what ways could the CEC be improved?

#### **Conclusion**

- Compared to what was covered in this interview, do you have any additional thoughts, examples, comments to share? Are there any important things that did not come up during the interview?

#### **Closing**

- Thank you very much for your availability. Can I contact you again, if there is any point to deepen?

**Themes of the interview to CEC Members (CM)**

1. Perception of the role of the CEC in the local context and reasons for being a member of the CEC.
2. Understanding of own activities in support of the CEC.
3. Understanding of strengths and weaknesses related to the service.
4. Reflection on service utilization and perception of its impact in the local context

#### **Opening**

- Good morning, thank you for your time to participate in this study. Regarding the purpose of the research, do you have any questions or concerns that you would like to clarify?
- Would you like to tell me about your experience with the CEC?

#### **Theme A – coherence**

- In your opinion, what is the role of the CEC within the Local Health Authority of Reggio Emilia?
- How, in your opinion, is it different from other promoted services and what is its specificity?
- What is the goal of the CEC and how does it act in the local context?
- What are the motivations that led you to take part in this service?

#### **Theme B – cognitive participation**

- What does your work involve in this service?
- What do you think about the value of a service like CEC to your work and your colleagues’?

#### **Theme C – collective action**

- Thinking about the activities carried out by the CEC, how have they been integrated into the local context?
- Are there any limitations - organizational/internal/external to the service/economic? – that you perceive with respect to the CEC? If so, which ones?
- Are there any potentialities with respect to the service that has been implemented? If yes, which ones?

#### **Theme D – reflexive monitoring**

- In light of what has been done in these 16 months, how do you evaluate the implemented service?
- In what ways could the CEC be improved?

#### **Conclusion**

- Compared to what was covered in this interview, do you have any additional thoughts, examples, comments to share? Are there any important things that did not come up during the interview?

#### **Closing**

- Thank you very much for your availability. Can I contact you again, if there is any point to deepen?
