# Supplementary material for: How to implement a clinical ethics committee in an oncological research hospital: Qualitative results from a process evaluation study using normalization process theory (EVACEC)
Source: PLoS One. 2025 May 6;20(5):e0318870. doi: 10.1371/journal.pone.0318870 (PMC12054913; doi:10.1371/journal.pone.0318870)
Supplement: S5 File — Interviews transcripts_raw data: this folder contains all the raw data used in this study, the interview transcripts (in original Italian). (ZIP) [file pone.0318870.s005.zip › S5_Interviews transcripts_raw data/01CM.docx]

CC: Esperienza in ambito di etica clinica/bioetica a livello professionale

PLD: (00:25) ho iniziato di occuparmi di bioetica clinica durante il master in cure palliative (*redacted*) con un particolare focus sull’ambito delle cure palliative e di fine vita, perché durante quel master che appunto era sul tema di cure palliative multidisciplinare, io da subito avevo approfondito quale poteva essere il ruolo dell’eticista all’interno dell’equipe di cure palliative e ho trovato un ambiente molto fertile per questo tipo di lavoro, perché in effetti quello che accade all’interno di questi team di cure palliative è molto spesso un tema che interessa l’etica clinica. Dopo, con il dottorato di ricerca in bioetica, ho approfondito con il mio progetto di ricerca di dottorato (01:15) quale poteva essere il supporto della bioetica clinica concretamente focalizzandomi sul tema dell’etica della comunicazione, sempre in cure palliative, e, da quando ho finito il dottorato, ho iniziato al lavorare in questo IRCCS oncologico a Reggio Emilia con un progetto sperimentale che riguardava non soltanto la ricerca, ma anche quali potessero essere le forme migliori di supporto etico e di bioetica clinica sempre in un ottica di ricerca per individuare gli outcomes di questo lavoro, quindi diciamo che è una esperienza decennale ormai e con questo progetto sperimentale di cui stiamo tuttora valutando non solo l’implementazione ma anche i vantaggi e gli svantaggi e le barriere. E poi dal 2020 sono presidente di questo comitato per l’etica nella clinica. (02:22)

CC: Ti chiedo se puoi raccontarmi anche un po’ dell’esperienza con il CEC appunto, come presidente del CEC e del CEC in generale.

PLD: (02:32) Questo ruolo di presidente è stato scelto diciamo perché serviva un presidente che non avesse soltanto un ruolo istituzionale ma che lavorasse, e che quindi avesse esperienza di bioetica clinica ma anche tempo da dedicare alla scrittura dei pareri etc. Ero stata componente di un comitato etico per la sperimentazione in qualità di esperto di bioetica, non avevo mai avuto esperienza prima in un CEC ma appunto avevo l’esperienza da bioeticista individuale che lavorava con l’equipe. Senza dubbio ho imparato facendolo perché un presidente, insomma, necessita di doti non soltanto sulla bioetica clinica ma anche organizzative di gestione di un gruppo in questo caso un gruppo di persone molto esperte, molto adulte con tanti anni di esperienza professionale in diversi ambiti (03:40) e quindi c’è un doppio binario: quello della bioetica clinica e quello organizzativo, e sull’ambito organizzativo ho imparato facendolo cercando sempre le modalità migliori per farlo, e come bioeticista clinico invece è stata un’esperienza che completa un percorso che sto facendo, il CEC stesso credo che completi un servizio di bioetica clinica perché sono due tipi di supporto etico completamente diversi, quello del CEC e quello del bioeticista, e quindi mi è sembrato di aggiungere l’ultimo tassello al servizio che avevamo costruito. Direi che è un’esperienza di completamento. (04:30)

CC: quali sono le motivazioni che ti hanno spinto a prendere parte a questo servizio?

PLD: (04:42) Io personalmente non ero molto convinta di istituire un CEC, pensavo che fosse sufficiente il servizio di bioetica così come era stato costruito e anzi quello che mi premeva era trovare un’altra figura che facesse anche consulenza etica, supporto etico, perché le persone che c’erano in quel momento erano persone che facevano ricerca, (*redacted*) quasi al 100% e le persone che collaboravano con noi nel 2020 pure, erano impiegate su progetti. Per cui mi premeva più trovare una persona che si formasse anche nel fare quello e non creare un CEC. Poi quello che è successo col Covid, le richieste che sono arrivate di consulenza mi hanno fatto capire invece quanto istituzionalizzare questo servizio potesse aiutare e in un qualche modo anche tutelare (05:41) chi faceva il lavoro di supporto etico, visto che la figura del bioeticista non esiste a livello istituzionale. E soprattutto ho cominciato a sentire la necessità della multidisciplinarietà. Quindi poi ho appoggiato questa idea che non è venuta da me ma è venuta dall’allora direttore scientifico. (06:04)

CC: quindi ti ha chiesto lui di ricoprire il ruolo di presidente immagino..

PLD: (06:13) Si, tutti i componenti di questo CEC sono stati scelti su chiamata, è un purposive sampling come si dice, e quindi anche il mio ruolo è stato scelto dal direttore scientifico, dal direttore operativo dell’IRCCS e dal direttore generale. Non è stato votato il presidente, come si usa fare nei comitati per la sperimentazione, così come non è stato fatto un bando per i componenti del CEC, ma sono stati selezionati sulla base dei curriculum, delle esperienze e delle conoscenze. Tutto questo è stato possibile farlo sia perché non esiste una normativa che regola la costituzione di questi CEC, ma anche perché essendo oggetto di un progetto di ricerca sperimentale questo ci ha lasciato libertà. (07:00)

CC: secondo te qual è il ruolo del CEC all’interno dell’azienda?

PLD: (07:13) Allora il CEC è una struttura che deve servire ad orientare le policy, e quindi dovrebbe essere chiamato in causa dai decisori che sono all’interno dell’azienda e quindi questo per quanto riguarda ad esempio i pareri su questioni generali su cui questo CEC può esprimersi, ed è un tipo di compito. Ma anche ad esempio se dovesse venire ad istituirsi una biobanca il CEC potrebbe collaborare nella scrittura del regolamento della biobanca. Poi il ruolo fondamentale è quello di supporto ai professionisti sanitari. Le modalità per farsi che questo tipo di supporto sia percepito come tale e funzioni sono oggetto del progetto di ricerca, quindi personalmente in questo momento continuo ad avere qualche perplessità rispetto a come questo CEC riesca a svolgere il suo ruolo effettivamente. (08:17)

CC: perplessità del tipo?

PLD: (08:20) io penso che il servizio di supporto etico sia funzionale e utile se entra nei reparti. Quindi il gruppo di saggi che si riunisce una volta al mese e che il professionista autonomamente deve contattare per ricevere supporto lo vedo una realtà molto lontana da quello che succede quotidianamente. Quindi io ho cercato di scrivere il regolamento di questo CEC si ispirandomi al comitato nazionale di bioetica al parere del 2017, ma anche basandomi sulla mia esperienza, e quindi puntando molto sulla flessibilità. Per esempio quindi abbiamo un calendario di sedute però se serve un’urgenza ci riuniamo e dovete essere disponibili a farlo, dovete essere disponibili eventualmente a creare dei sottogruppi che vanno in reparto. Nonostante questo io vedo che, probabilmente perché io faccio questo lavoro da più anni, però i professionisti preferiscono fare riferimento a me e magari avere la consulenza di un singolo che la mattina alle 8 va all’interno della riunione di equipe e gli porta un punto di vista diverso, piuttosto che fare tutta la procedura formale, rivolgersi ad un CEC. Perché lo vivono più (09:44) come, appunto, un supporto istituzionale. Per cui si rivolgono di solito i professionisti che magari hanno bisogno di un parere scritto, cosa che il bioeticista singolo di solito non produce, a meno che non si sia espressamente richiesto, perché il bioeticista singolo lavora con l’equipe, viene in qualche modo a fare parte dell’equipe, invece questo CEC viene vissuto come qualcosa di istituzionale, e quindi questo è un vantaggio se i professionisti hanno bisogno di qualcosa di scritto, perché magari vogliono condividerlo con l’equipe, perché magari c’è stato un conflitto grosso con l’equipe. Diventa uno svantaggio se magari non vogliono che resti qualcosa di scritto, per tanti motivi. E allora dicono va be meglio che chiamiamo il bioeticista che ci aiuta, ci dà un supporto e andiamo avanti così piuttosto che rivolgerci ad un CEC. Oltre al fatto che il formalismo, che noi abbiamo cercato di ridurre al minimo, ma che comunque ci deve essere. Quindi il fatto di pigiare un bottone, fare una richiesta, produrre una relazione, allegare i documenti è qualcosa che blocca dei professionisti che vanno sempre di fretta e hanno sempre bisogno di tempo per fare le cose. E paradossalmente, da un occhio esterno che non è clinico, può sembrare assurdo che non trovino il tempo di allegare dei documenti o scrivere una breve relazione, invece io ad esempio lo capisco, penso che sia molto più facile alzare il telefono e chiamare. (11.23) E poi quello che ho cercato di fare in quest’ultimo periodo, non dall’inizio, perché ci ho pensato dopo sinceramente, è stato quello di selezionare io le richieste che arrivavano a me, e quindi poter suggerire, alcune, di mandarle al CEC. Quindi questo per tornare anche alla prima domanda, credo che un presidente di un CEC debba essere uno profondamente esperto di etica clinica, che riesca a capire il tipo di richiesta, se è idonea, come il CEC può affrontarla (se in un piccolo sottogruppo o collegialmente), che tipo di risposta serve e, nel nostro caso, visto che esiste un servizio di bioetica clinica anche individuale, capire qual è la forma di supporto migliore. Ecco, penso che questi CEC debbano far sempre parte di una proposta un po’ completa (12.20 problema tecnico), affinché sia utile all’azienda questo, e poi affinchè sia utile all’azienda va’ fatto un bombardamento informativo sul servizio, non basta la diffusione di una news, ma neanche di 10 news, non basta la formazione, perché non è sufficiente. Quello che ancora oggi riscontriamo dopo 2 anni è che ancora molti dicano “Ah, si esiste un CEC? Non lo sapevo”. E non so quali siano le modalità migliori per fare questa diffusione perché dire che noi ci siamo impegnati molto. Il servizio comunicazione ci ha aiutato moltissimo, news, materiale, formazione, formazioni molto partecipate, eppure certi messaggi non passano. E quindi penso che non sarà abbastanza utile fintanto che non ci sarà un attenzione a livello istituzionale. Ad esempio c’è chi dice che questi CEC devono far parte della direzione sanitaria. Solo se fanno parte della direzione sanitaria allora vengono percepiti come uno strumento, pertanto alcuni dei problemi che arrivano alla direzione sanitaria, come la medicina legale, vengono girati al CEC che diventa l’istituzione deputata a risolverli, questa potrebbe essere una soluzione. Mi riferisco proprio ad iniziative di tipo istituzionale più che di diffusione delle informazioni, è fondamentale affinché funzioni. Forse il posizionamento in direzione sanitaria va’ in quella direzione (14:15).

CC: Si perderebbe però un po’, tra virgolette, l’indipendenza del CEC

PLD: (14:21) Ma non penso, nel senso che è importante veicolare il messaggio che non è la direzione sanitaria, semplicemente da un punto di vista strutturale è all’interno della direzione sanitaria, anche adesso non è del tutto indipendente, perché è promosso dall’unità di bioetica e quindi all’interno della direzione scientifica, ma sai, adesso c’è questo alone di progetto di ricerca per cui ci può stare, ma quando il progetto di ricerca si concluderà, quello per cui tu mi stai intervistando, andrà posizionato da qualche parte no? E io credo che l’unica posizione possibile sia la direzione sanitaria. O a tutt’al più la medicina legale, ma comunque la medicina legale è dentro la direzione sanitaria. (15:12)

CC: sulla base della tua risposta, le persone che fanno una richiesta a te come servizio di bioetica, che tu decidi di veicolare sul CEC sono già consapevoli che esiste CEC? Cioè, chi conosce l’unità di bioetica e il tuo lavoro già conosce il CEC?

PLD: (15:42) No, non necessariamente. L’ultima richiesta che abbiamo ricevuto.. allora, noi abbiamo avuto un numero alto rispetto alla media internazionale, non alto in assoluto quindi, credo che abbiamo avuto circa 6 richieste, su una media internazionale che è 2-3, se non sbaglio. Mi è capitato, l’ultimissima in ordine di tempo, è di un gruppo sulla nutrizione artificiale, che vuole un supporto nella scrittura delle linee guida per l’attivazione della nutrizione artificiale nei pazienti con un certo tipo di patologia, non mi ricordo quale. Loro hanno fatto richiesta al CEC pensando di farla all’unità di bioetica, nonostante siano molto ben divisi i siti. Questo per dire, no, anche la confusione. In realtà la richiesta però è da CEC, perché solo un CEC ti può aiutare nel (16:45, si sovrappongono) delle linee guida, quindi poi io ho spiegato loro la differenza e loro hanno detto “no no vabbe, a noi ci serve quello” quindi va bene il CEC. Però, questo per rispondere alla tua domanda no? E non è bastato sgolarsi sulla differenza. Tutte le formazioni iniziavano con la differenza, no? Che l’unità di bioetica o il mio lavoro sia più conosciuto è normale, lo faccio dal 2016, ci sta, il CEC è dal 2020. Però io mi rendo conto che come singolo ho modo di entrare dove voglio abbastanza facilmente, invece il CEC questo discorso di entrare nelle dinamiche vedo che si fa molta difficoltà. (17:26)

CC: L’eterna lotta tra il pro della multidisciplinarietà e il limite però dal fatto di avere tanti esperti che devono accordarsi con i tempi, organizzarsi e comunque non possono partecipare in massa alle riunioni delle equipe, quindi. Effettivamente, sono necessità difficilmente conciliabili con le caratteristiche del servizio stesso. Ti chiedo se hai qualcosa da aggiungere sul tema proprio della specificità del servizio del CEC rispetto ad altri servizi promossi in azienda come appunto l’unità di bioetica o altri servizi che..

PLD: (18:13) non ho niente da aggiungere. Penso di averlo già detto. Non mi viene in mente nient’altro.

CC: Si si. Invece, ti chiederei un po’ di approfondire l’obiettivo del CEC, in che modo agisce nel contesto locale dell’azienda. Se hai altre cose da aggiungere anche qua.

PLD: (18:36) Oddio, rispetto a quello che ho detto. Cioè, forse l’unica cosa che si potrebbe aggiungere è che questo CEC va a colmare un vuoto che c’è attualmente in Regione Emilia Romagna, quindi la sperimentazione di questo CEC in qualche modo è stata anche spinta, dalla Regione che non ha dato un mandato ufficiale due anni fa, ma poi ha mostrato interesse, anche organizzando il convegno regionale del 6 di aprile di quest’anno, ad una riorganizzazione regionale, nell’ottica di un panorama nazionale che si sta muovendo. Perché anche ieri mi hanno mandato un articolo di Furlan uscito sulla rivista italiana di cure palliative, in cui si promuove l’organizzazione di una rete nazionale di CEC pediatrici. Mi sono arrivati, in questi due anni, l’ultimo in ordine di tempo due settimane fa da Trento, la spinta ad organizzare una rete nazionale di CEC, quindi di mettere insieme le realtà già esistenti. Per cui credo che per Reggio Emilia, che è un IRCCS, produrre evidenze su questo tema possa essere valore aggiunto, no? Cioè dire: d’accordo, i CEC funzionano, ma solo se fatti in questo modo, quali outcomes abbiamo individuato. Questo penso possa essere il valore aggiunto in un’azienda che ha al suo interno un IRCCS, ovviamente. Perché questa è la conditio si ne qua non. Poi direi nient’altro in più (20:13)

CC: anche alla prossima parzialmente hai già risposto, ma magari puoi entrare un po’ più nel dettaglio sul tuo lavoro all’interno del CEC

PLD: (20:32) dunque io sono presidente, forse sarebbe stato meglio chiamarlo coordinatore, più che presidente, perché mi sembra più un ruolo di coordinamento. Io, appunto, oltre a valutare le richieste che arrivano al CEC, e quindi capire se indirizzarle o meno al CEC, se sono idonee e opportune, mi occupo della redazione delle risposte a queste consulenze, sulla base, chiaramente, del verbale e di quanto emerge durante la discussione in plenaria, poi la bozza di queste risposte viene riletta e corretta da tutti i componenti del CEC, ma io produco la prima bozza. Che è una operazione che richiede tempo, quindi da tenere in considerazione anche questo quando si organizza un CEC, tutto il lavoro di back office che c’è dietro. Nel senso che, mentre una segreteria scientifica di un comitato per la sperimentazione gestisce la documentazione e scrive il verbale, questo non può farlo una segreteria scientifica di un CEC, perché è una responsabilità dei componenti del CEC. Io ho preso naturalmente questo ruolo perché non avrei saputo a chi altro assegnarlo, non avrei potuto chiederlo ad uno dei miei colleghi di farlo. Quindi poi redigo la versione finale della risposta raccogliendo tutti i pareri e conduco le discussioni, quindi cerco in un qualche modo anche di dare un metodo ai miei colleghi che, per quanto siano illustri esperti nei loro campi, magari sulla bioetica clinica non sono così tanto esperti. Per cui conduco le riunioni e cerco di fare un po’ anche di informazione sui metodi di analisi dei dilemmi etici. (22:40)

CC: è stata mai fatta formazione ai membri su questo aspetto?

PLD: (22:46) No, ed è stato un errore. Assolutamente, una cosa che va implementata. Nel processo di costruzione di un CEC va implementata, deve essere lunga. Poi dopo che ho letto l’articolo di Magelsen (?) su come fanno loro, fanno 3 anni di formazione continua ai componenti del CEC, quindi penso che con le risorse si possa fare un po’ tutto. Magari non tre anni, però sicuramente un corso di formazione prima ben fatto va’ proposto. Che sia un corso in FAD, che sia un corso in presenza ma va fatto assolutamente. E poi va’ fatta formazione continua, va ripreso continuamente. E un’altra cosa che va fatta a livello di formazione e di implementazione del percorso secondo me è la condivisione di un metodo di analisi. Quindi, che sia un sistema six-step model, che sia un idea qualsiasi cosa ma va condiviso e va’ adottato. Perché quello serve a formare ma serve anche ad avere sempre un punto di riferimento. (23:49)

CC: Potenzialmente è più efficace il processo proprio che adottando un metodo del genere. Secondo te l’impatto, cosa pensi del valore che ha un servizio come quello del CEC per i professionisti sanitari?

PLD: (24:11) Secondo me un valore immenso, se lo capissero. Ma non è colpa loro. Si possono individuare mille difetti ai professionisti sanitari ma non che non siano ricettivi. Lo sono per natura e per professione. Per la natura della loro professione. È che bisogna trovare le modalità per parlarci. È il CEC che si deve adattare ai professionisti, a mio giudizio, e non viceversa. Quindi se non arriva abbastanza, se l’informazione non arriva abbastanza, bisogna farsi la domanda del perché. Se viene percepito come un’istituzione formale e lontana dalla realtà quotidiana bisogna domandarsi perché. Io penso che in generale qualsiasi forma di supporto etico sia indispensabile e non soltanto in alcuni ambiti. Io sono specializzata in cure palliative, ma secondo me non c’è ambito che non richieda una forma di supporto etico, che è completamente diversa da qualsiasi tipo di supporto psicologico si possa attuare, però va fatta con metodo. E anche un CEC deve individuare un metodo scientifico, perché qua parliamo di scienza. E quindi il CEC deve dotarsi dello stesso metodo. E dare risposte che siano concrete, evidence-based ovviamente rapporto all’etica, che non è evidence-based per definizione però in un contesto … (CC: dare delle risposte motivate) si, si ma anche utili, concrete, poi si aggiunge il punto di vista etico perché se no non ci sarebbe la particolarità, però deve essere tutto molto operativo. (26:06)

CC: pensando all’attività che svolge il CEC, in che modo secondo te sono state integrate nel contesto locale?

PLD: (26:20) di sicuro, appunto, il fatto che Reggio Emilia fosse una realtà molto ricettiva sulla bioetica ha aiutato. Sono state integrate bene da quel punto di vista e questo magari ci ha portato ad avere più richieste rispetto alla media internazionale. Non ancora abbastanza, senza dubbio. Anche oggi la medicina legale ci ha attivato su un caso, ma ha attivato me come bioeticista clinico per un confronto che secondo me era benissimo da CEC. Ma non è venuto in mente neanche alla medicina legale il cui direttore è dentro al CEC (26:59)

CC: Qualcuno me lo hai già detto di limiti intrinseci del CEC, ce ne sono altri che ti vengono in mente?

PLD: (27:18) no, direi oltre quelli che ho detto no

CC: invece potenzialità che intravedi nel CEC, rispetto a implementazioni del servizio, possibili attività che magari ti piacerebbe che svolgesse

PLD: (27:38) allora la potenzialità principale è che diventi istituzionale, nel senso che si chiuda la sperimentazione e diventi un servizio dell’azienda, finanziato (fondamentale). Questo CEC può essere un modello per tutta la regione, e questa è un’altra grossissima potenzialità perché comunque portiamo un’esperienza, e quindi gli altri CEC nascenti possono e dovrebbero secondo me regolarsi sull’esperienza di Reggio. Ovviamente mettendo in conto che anche il CEC di Reggio può cambiare. Quando uno chiude questa sperimentazione raccoglie dei dati, lo modifica in base ai risultati. Però comunque può rappresentare un modello e continuare sicuramente a far si che Reggio Emilia sia su questo argomento ferrata e quindi sia presa come punto di riferimento. Questa la vedo come potenzialità principale. E poi arrivare davvero a tutti i professionisti. Quindi anche magari modificare il regolamento in tal senso. E quindi le funzioni ma soprattutto le modalità con cui queste funzioni del CEC si sviluppano. (28:50)

CC: ma hai già in mente delle migliorie possibili al regolamento che permettano di includere più professionisti?

PLD: (28:59) si, ad esempio vorrei inserire nel regolamento questa selezione dei problemi che arrivano al presidente ma non solo, cioè tutti i componenti del CEC devono avere per regolamento la possibilità di gestire la richiesta che arriva come una richiesta gestibile dal CEC, quindi, così come lo faccio io, se al medico palliativista arriva una richiesta che secondo lui è da CEC può girarla al CEC, e questo è importante inserirlo nel regolamento. Così il medico legale e tutti gli altri professionisti interni. Ovviamente, gli esterni non lo possono fare. Vorrei enfatizzare la flessibilità, il lavoro in sottogruppi e la relazione sempre con la direzione sanitaria rispetto alle policy, per cui vorrei che la direzione sanitaria ogni volta che fa una riorganizzazione degli ospedali perché c’è un’ondata Covid ci coinvolgesse. O i vaccini. O a campagne informative rispetto a degli screening particolari. Secondo me ci sono tantissime questioni e questo verrebbe aiutato dalla diffusione …? (30:19) sia dal regolamento che dal legame con la direzione. (30:24)

CC: mi riallaccio ad una cosa che hai detto prima sul fatto che ti auguri che con l’istituzionalizzazione il CEC venga anche finanziato, prima abbiamo parlato dei limiti solo interni del CEC, ma questo per esempio è un limite al servizio del CEC attualmente?

PLD: (31:14) si, diciamo che fare lavorare in forma di volontariato secondo me non funziona. O tu decidi di fare volontariato, ed è un discorso. Ma il CEC non può essere oggetto di volontariato. È un lavoro. (31:37 si interrompe video, riparte come segue) (CC: dicevi che non può essere un attività di volontariato) No no assolutamente, innanzitutto per la gestione dei dati con cui entriamo in contatto: entri in contatto con dati personali dei pazienti, per forza di cose è inevitabile. Quindi già questo non lo può prefigurare come un’attività di volontariato. Quindi è un’attività lavorativa vera e propria e in quanto tale necessità di contributo per l’impegno, che non è soltanto quello delle sedute ma anche quello di back office. Noi ne abbiamo saltate due di sedute, una perché non avevamo raggiunto il numero legale e l’altra perché era agosto e quindi non era proprio calendarizzata. Non c’è stata seduta in cui non abbiamo avuto comunque qualcosa da fare, perché anche quando non arrivavano richieste avevamo da scrivere un parere, poi ci siamo preparati rispetto alla questione del suicidio assistito quando si pensava di dare questo compito ai CEC, la cosa è ancora in discussione perché il ministero della salute ha individuato i comitati per la sperimentazione ma alcune Regioni hanno chiesto comunque, tra cui l’Emilia Romagna, che il compito andasse ai CEC. Quindi se passasse anche questa cosa il gettone di presenza sarebbe assolutamente dovuto proprio, sono anche tante ore di lavoro. Gli esterni non so come lo facciano, rispetto ai loro orari di lavoro - molti non hanno un orario di lavoro in realtà, perché sono ricercatori che non hanno un cartellino tipo (*redacted*)mi viene in mente o (*redacted*), altri sono in pensione quindi non hanno il problema. Gli interni lo fanno in orario di servizio, quindi sono ore che tolgono ad altre attività. Va regolato anche da quel punto di vista, se non lo si fa è un limite (33:38).

CC: altri limiti esterni al CEC ma che in qualche modo ne ostacolano…

PLD: (33:45) ma secondo me il riconoscimento a livello normativo è il limite principale. Fin quando non c’è un riconoscimento normativo si fa fatica a farli esplodere. E credo che l’organizzarsi in una rete nazionale possa essere di aiuto anche per spingere sulla normativa. Secondo me sarà un passaggio importante, per quello che è nel mio potere lo promuoverò tantissimo perché ci credo molto. Secondo me mancano le evidenze sull’utilità, che vanno prodotte. E questo è un altro limite esterno. Perché anche a livello internazionale non ce ne sono. Vengono descritte delle esperienze, vengono descritti dei modelli ma rispetto alla ricerca degli outcomes siamo ancora molto indietro non sappiamo ancora neanche quali sono. Diminuiscono il moral distress, questi CEC, degli operatori? Impattano sulla qualità della vita dei pazienti? E quindi individuare gli outcomes, vedere come valutarli. Vedere se ci sono, se dei risultati ci sono. Anche questo potrebbe essere un limite esterno. Cioè il fatto che non ci siano evidenze. (35:00)

CC: Ti chiedo invece adesso una valutazione complessiva del servizio e delle attività che sono state implementate in questo tempo di attività del CEC

PLD: (35:21) la valutazione complessiva è buona. Soprattutto durante l’ultima consulenza che abbiamo fatto, in cui il professionista, il medico che aveva fatto domanda è venuto anche in audizione nella seduta di inizio luglio, ho pensato, in seguito a quella seduta, “è davvero utile” questo strumento, aiuta veramente tantissimo i professionisti. Un po’ perché l’audizione è stata molto interessante, un po’ perché il medico ce l’ha detto, ci ha detto “grazie, mi avete aiutato tanto”. Un po’ perché ho visto quello che è successo dopo: noi abbiamo scritto un parere molto lungo di 6 pagine sulla questione che ci portava il medico e lui ha deciso di condividerlo con tutta la sua equipe chiamandomi in quel caso. Io durante quella seduta mi sono occupata di redigere la risposta, però non sono intervenuta direttamente perché mi sentivo molto coinvolta, parte dell’equipe – perché ero intervenuta come bioeticista – quindi ho redatto la risposta ma nella discussione ho cercato solo di condurla senza entrare troppo nel merito e il medico curante ha deciso poi di condividere questa risposta del CEC con tutte le sue equipe e questo mi è sembrato un ottimo risultato, perché credo che abbia trovato degli spunti di riflessione interessanti. Quindi, soprattutto alla luce di quest’ultima esperienza io penso che non si possa che dare una valutazione positiva con ampi, ampissimi margini di miglioramento e soprattutto la richiesta ufficiale (si interrompe il video, 37:09) questa iniziativa non può che essere seguita dalla formalizzazione famosa di cui parlavo prima, che servirà per farlo funzionare. Ecco se non dovessi riuscire in quell’ottica per me la sperimentazione si chiude, nel senso che proprio in quanto sperimentale dopo tre anni, il mandato è di quattro anni, ma essendo oggetto di sperimentazione io posso dire i dati che abbiamo raccolto sono questi, i termini per migliorarlo sono questi altri, se non si realizzano si può chiudere perché così credo che non faccia passi avanti. Credo che potremo continuare ad avere una media di un paio di richieste, ma non credo che così faccia differenza come servizio. E siccome è molto time-consuming e non me la sento di chiedere ancora ai componenti questo sforzo, se non c’è un investimento economico e strutturale (CC: non ha senso mantenere il servizio). Questa è la mia valutazione complessiva. (38:17)

CC: rispetto alle attività che avete svolto di più all’inizio, noti qualche differenza in questi più di 16 mesi di attività rispetto alle cose che facevate, cose che magari avete iniziato a fare più avanti.

PLD: (38:42) no, siamo partiti.. all’inizio ci siamo concentrati più sui pareri perché c’era l’entrata in terapia intensiva e il triage del Covid e poi c’era l’etica della vaccinazione. Però diciamo che le attività sono rimaste uniformi, c’è stata la scrittura di pareri su questioni generali, l’attività di consulenza (che è un po’ cresciuta nel tempo) e la formazione che abbiamo concluso. È chiaro che nella programmazione del prossimo anno sicuramente reinserirei della formazione, ma soprattutto auto-formazione. Ma a livello di modalità non è cambiato molto, è rimasto come da regolamento. (39.25)

CC: tra le attività che svolge il CEC, secondo te che cosa ha più presa sugli operatori sanitari, su chi vi chiede supporto?

PLD: (39:35) La consulenza su casi singoli. Perché i pareri servono più per un dibattito pubblico che per i professionisti sanitari, quindi tu puoi dire che il CEC si posiziona in questo modo e non mi sono sembrati molto utili neanche per la direzione sanitaria nel momento in cui dovesse prendere delle decisioni strategiche. Direi che per i professionisti sanitari l’attività di supporto etico sui loro casi singoli è quella più utile, che ha più impatto. Quella su cui puntare di più, secondo me.

CC: l’ultima domanda sarebbe in che modo il CEC potrebbe essere migliorato, mi sembra che ne abbiamo già parlato diffusamente.

Niente da aggiungere
