# Supplementary material for: How to implement a clinical ethics committee in an oncological research hospital: Qualitative results from a process evaluation study using normalization process theory (EVACEC)
Source: PLoS One. 2025 May 6;20(5):e0318870. doi: 10.1371/journal.pone.0318870 (PMC12054913; doi:10.1371/journal.pone.0318870)
Supplement: S5 File — Interviews transcripts_raw data: this folder contains all the raw data used in this study, the interview transcripts (in original Italian). (ZIP) [file pone.0318870.s005.zip › S5_Interviews transcripts_raw data/02CM.docx]

*Qual’è la tua esperienza in etica, in ambito bioetico?*

La mia esperienza è fondamentalmente connessa alla (*redacted*) perché ho iniziato a ragionare dei temi etici e bioetici al mio Master di cure palliative (*redacted*) anni fa dove appunto nella mia classe c’era appunto (*redacted*) e con la quale ha fatto la tesi di master sulla comunicazione in ambito oncologico che è un ambito chiaramente di cure palliative …………………… Dopo di che l’approfondimento etico ha continuato con la tesi di dottorato questa volta della (*redacted*) che era invece qua a Reggio Emilia intervistando i professionisti e anch’io stessa lo feci su questioni di tipo etico e quindi diciamo che secondo me la visione più ampia rispetto alle problematiche che io incontravo nella clinica sicuramente avendo frequentato lei, me l’ha data parecchio lei. E poi negli anni come tu ben sai abbiamo fatto quel corso in particolare su quello cioè il corso di etica ……….. di cure palliative fino dopo ad arrivare alla partecipazione a questo comitato, quindi è un po’ diciamo una attenzione un avvicinamento all’argomento tratto dall’incontro che è stato quello con la (*redacted*), poi è chiaro che ci sarà anche una sensibilità mia, cioè io vengo comunque da una cultura anche cattolica in cui certe domande rispetto alle persone fragili me le faccio, cioè non per nulla ho scelto di fare la disciplina che faccio ecco perché comunque non è che mi interessava semplicemente la persona alla fine della vita ma mi interessavano le tematiche, le cose che si andavano a quei tempi, a fare in questa fase di malattia ………………………….ma anche tutte le questioni di etica, di autodeterminazione, di tutela dei fragili, scelte, decisioni insomma tutto quest’ambito qua. Così insomma un mix personale, professionale …………mi hanno aiutato a ……….. in questo campo qua.

*Rispetto alla tua esperienza con il comitato per l’etica nella clinica?*

Ma guarda io mi sono avvicinata a questa cosa con estrema umiltà e ………… l’ascolto perché quando mi è stato chiesto di partecipare volentieri ho detto di sì però non ero assolutamente, cioè non sapevo assolutamente cosa mi avrebbe aspettato e se ero in grado, se avevo le competenze per contribuire in qualche modo a questo comitato, capito? Quindi non è che come altre cose per esempio se uno mi dice vai a vedere o vai a partecipare all’unità di cure palliative intra-ospedaliere io so qual è un certo livello e so cosa andare a cercare e so cosa aspettarmi. Per il comitato per l’etica nella clinica io non sapevo assolutamente cosa aspettarmi e quindi ci sono andata proprio in ascolto. No questo per dire che la mia esperienza sicuramente è positiva ma perché credo che già solo nel mettere delle persone competenti in tanti ambiti di un certo livello come sono alcuni componenti del comitato etico nella clinica attorno ad un tavolo e discutere di situazioni che creano dei dilemmi agli operatori è già tantissimo, per cui è un’esperienza sicuramente positiva anche perché partivo con nessuna aspettativa ecco era tutto di guadagnato, capito? Era tutto da costruire e non avevo termini di paragone; invece immagino che chi l’ha messo su cioè si sia documentato abbia visto cosa c’era in giro, cosa non c’era in giro ma dal mio punto di vista per me era tantissimo già aver solo costituito questo comitato perché nella nostra esperienza l’unica direzione etica che un po’ ci veniva data era quando l’eticista ci faceva la supervisione d’equipe, al di la di quello era veramente tabula rasa non c’era niente, cioè il comitato etico non è una cosa del genere non aiuta per niente in questa cosa solo è solo diventato una macchina burocratica quindi io per il comitato etico per la clinica io avevo solo - mi faceva solo piacere partecipare, ecco capisci non è che. Quindi l’esperienza è buonissima.

*Quindi le motivazioni che ti hanno spinto a prendere parte a questo servizio alla luce anche di questa situazione d’incertezza che mi hai un po’ descritto?*

Sicuramente crescita mia cioè una delle motivazione è crescere io in quest’ambito e mettermi a confronto su certe questioni, sicuramente. Secondo per dare un aiuto più concreto, per dare un aiuto perchè secondo me non c’è niente a parte un clinico come me si trova ………………………….. Queste due sono state sicuramente le motivazioni principali. Ah e poi la curiosità, la curiosità di partecipare a qualcosa che secondo me era estremamente innovativo. Mi piace andare un po’ su questi argomenti su questi ambiti che io vedo di frontiera, di frontiera non nel senso ai margini, ma nel senso di scoperta, no. Quindi la motivazioni è questa, sì.

*Qual è il ruolo che ricopre il CECH all’interno dell’Azienda?*

Il ruolo è quello di riuscire a rispondere ad aiutare i clinici …………… quando si trovano ad affrontare delle questioni etiche dove sono incastrati in cui non sanno da che parte muoversi, almeno io vorrei aiutarli in questo senso aiutarli nel ragionamento e a rendergli un po’ più chiara la situazione che hanno davanti, aiutarli quindi a fare il bene del paziente. Sicuramente credo che non sia ancora entrato nelle corde dei nostri clinici, ci sia ancora qualche barriera da superare. Questo sì.

*Secondo te quali sono queste barriere?*

Io credo ci sia molto lo spauracchio della burocrazia cioè nel senso di dire …………………… capita a me …………… ultimamente anche quella psicologa ……………….. secondo me meritava una consulenza nostra o del comitato per l’etica nella clinica invece vabbè ha chiesto a me io invece qualche risposta glie l’ho data perché non posso scindermi ……………………………………. tutto al palliativista che poi non risponde più a niente e manda tutto al CE. Poi ad esempio io ho avvertito proprio anche questa volta la difficoltà per la parte burocratica che viene percepita anche se in pratica è all’osso però viene percepita come una cosa in più. E l’altra cosa è che secondo me non siamo abituati, cioè io non ero abituata, poi figurati gli altri che non fanno neanche le cure palliative ma altre discipline, non siamo abituati a ragionare in quest’idea a questo livello capito?, quindi c’è sempre anche un alone di mistero, questo comitato etico per la clinica qualcuno diceva bho farò le domande giuste? Sì alla fine mi ha risposto la Tanzi, cioè secondo me c’è anche un timore di non saper porre bene le domande, capito? Perché non siamo abituati ad un ragionamento etico e far un ragionamento a quel livello lì che magari è davvero non è puramente etico, ma poi magari dopo ti aiuta il confronto col comitato a capire qual è il livello etico in cui impostarlo e poi la parte burocratica Uno in sé perché secondo me quando c’è etica si pensa al comitato etico …………………. sperimentali e l’altra perché ormai in ospedale la procedura, tutto quindi penso sia un deterrente, ma poi penso anche tanto la parte di sentirsi competenti nel fare la domanda giusta e anche del non far la figura da fagiolo, cioè non fare delle figure di merda quando invece magari non è una questione così difficile e dici magari anche solo confrontandomi con i miei colleghi e più o meno ne son saltato fuori invece di andare di fronte a delle persone esperte così insomma dai è sempre un certo imbarazzo.

*In che modo questo CEC è diverso da altri servizi che sono stati promossi dall’Azienda, qual è la sua specificità?*

La specificità è che risponde solo lui per queste questioni qua. La specificità sta proprio ………………………. È proprio far capire questo. Io proprio penso sia più importante di altri servizi che fornisce l’Azienda solo che è un pochino più difficile da far capire Perché noi, vedi cioè anche vedendo i miei colleghi di consulenza che mi chiedono siamo pieni a livello ospedaliero di problematiche etiche un po’ non le vediamo, un po’ siamo abituati a sbrigarcela da soli o ben che vada a buttarla sulla medicina difensiva e quindi ……………………….. medicina legale, ……. Ma è un’ipostazione completamente sbagliata anche un po’ culturalmente …………………… …………….. perché in realtà invece è un servizio che ha una specificità intrinseca di aiutarti dove nessun altro ti aiuta a risolvere i problemi ………………………….. di nessuno ……………………………………con la tua coscienza la tua cultura se ne hai, la tua capacità di ragionare sai che te lo fai però è altamente specifico ma non penso che sia ben compreso.

*In che modo le attività che sono state promosse in questi quasi 2 anni dal CEC sono state integrate nel contesto locale?*

Come si potrebbe fare - questi 2 anni sono niente nel senso che l’unità di cure palliative intra-ospedaliera perché qualcuno iniziasse a capire quello che facciamo ecc. ci son voluti 5/6 anni, ok? 2 anni son veramente pochi e poi 5/6 anni in cui noi abbiamo fatto in continuazione come dei martelli pneumatici ….……….. formazione e ricerca ………………… adesso poi ……..….. però, cioè martellando non solo dal punto di vista di formazione ma martellando anche dal punto di vista che la mattina ci vedevano in corridoio e li coinvolgevamo nella ricerca e questo ha fatto sì che diventassimo un unità inserita dentro l’ospedale cioè non è bastato metterci in pianta organica, poi è chiaro che ci sono state delle cose strutturali che ci hanno favorito, cioè quando hanno fatto il CORE, cioè ti faccio questo esempio perché per me sono stati dei passaggi che in effetti hanno aumentato l’integrazione dell’UCP e penso che alcune cose potrebbero essere riprodotte anche all’……………….. …………………………………………Cioè ho presente la domanda che mi hai fatto -stavo dicendo per dire quando siamo passati al CORE e fisicamente ci hanno trovato un posto in cui fisicamente c’è scritto sezione unità Cure Palliative e sull’ascensore c’è scritto dove siamo anche quello ha aiutato hai capito? Invece prima eravamo una specie di succursale della medicina oncologica senza targhetta. Cioè il fatto che anche il comitato per l’etica nella clinica è un entità on line, faccio un esempio, no? Senza uno spazio fisico senza una concretezza, questo potrebbe essere anche un passaggio che bisogna fare, capito? ……………….. fisicamente l’opposto, però essere identificati nell’organigramma da qualche parte e poi sicuramente fare, e attività non solo pubblicitaria, cioè anche se ci stiamo su Intranet, ma anche di formazione non perdere mai l’occasione per dire che ci siamo lo so. Tutti i comitati etici ………………… Faccio degli esempi no? Ogni tanto che alcune di queste figure compaiano proprio in corsia chiaramente in modo strutturato, non fare un giro e uno si chiede chi cazzo è questo qua cioè anche fare delle consulenza quando si riportano per dire il parere del comitato etico, che sia una persona fisica che lo riporta al clinico e che la volta dopo quindi si sia costruito anche questa piccola relazione capito? Secondo me sono cose che aiutano perché se un servizio ha la velleità di dare aiuto ai clinici secondo me questo servizio lo devono vedere lo devono toccare, sennò non capiscono che ne possono usufruire come non so, il servizio di medicazione delle ferite, ti faccio un esempio, cioè oggi secondo me è anche poco tempo perché comunque bisognerà fare una fase di come muoverti appunto perché credo sia una novità non solo per me , ma proprio una novità …………………………………….. per capire anche i nostri limiti le nostre potenzialità, come muoversi ecc. quindi però alcune cose diciamo per rendere meno aleatoria la questione e più concreta si possono fare secondo me all’interno di un ospedale o di una Azienda.

*Hai parlato di una mancanza a cosa ti riferivi nello specifico?*

No, non mi ci ritrovo in questa parola (mancanza) No dicevo che secondo me si è partiti anche per quello che si poteva sapere anche se secondo me si poteva sapere ben poco e poi è chiaro che non è partita a bomba frequentando i reparti e facendo pubblicità da tutte le parti già con un luogo fisico perché bisognava anche capire come muoversi quali erano i nostri limiti o che… come poteva essere messo in pratica questo comitato per l’etica ecc. le sue procedure dalle più importanti alle meno importanti, ecco, questo dicevo.

*Hai riscontrato delle potenzialità rispetto al servizio che è stato implementato?*

Beh tantissime le potenzialità perchè comunque è molto bello il confronto che si ha con le persone con le componenti sia bioetiche intendo che etiche che ci sono lì dentro e di quelli esperti delle Leggi, cioè ha delle potenzialità …………………….. anche solo perchè di solito questa è una cultura che chiunque si fa un fai da te anche magari leggendo i giornali, quindi è una cultura che noi non la rimandiamo ai libri, non la studiamo bene ma ce la costruiamo durante la nostra professione e quindi qui ha grandissimi limiti facendo riferimento a delle fonti …………........ Invece lì è come se uno avesse la potenzialità di poter avere un confronto diretto con le fonti delle questioni però ce li hai lì ha portata di mano di persone che sono competenti e che ti rispondono in modo molto molto puntuale. Per cui la grande forza è quella, è la visione multipla di tanti occhi che vedono la stessa questione da tanti punti di vista, ma per arrivare appunto a consigliarti qualcosa di veramente sostenibile. Secondo me la grande potenzialità è quella aver messo attorno al tavolo ste competenze qua e che sono adesso alla portata, alla portata di chi va poi al letto del malato.

*RIFLESSIONE DELL’INTERVISTATORE*

Questo è il messaggio che bisogna far passare è che bisogna capire in che modo perché viene visto come qualcosa se c’è c’è come una barriera ………………………pratica nella clinica, ma se invece passasse il messaggio che in realtà ti aiuta a maneggiare di più temi che secondo me stanno a cuore a noi clinici, che affrontiamo alle volte facciam fatica a riconoscere, che ci incasinano sicuramente il cuore e la mente, se capissimo appunto questo comitato ci aiuta ad affrontare questi aspetti qua è una grandissima potenzialità di cui i clinici hanno bisogno. Però ci son delle altre cose che sono più delle minuzie che riducono l’accesso o limitano l’accesso o fan sì che al clinico lo veda con un po’ di timore. Però è proprio questa invece la cosa che bisognerebbe far capire, che i clinici dovrebbero capire cioè nel tentativo di ………………………..questione ti vai a leggere degli articoli ti vai ad ascoltare dei programma alla tv, ti fai degli approfondimenti da solo che invece lì in 4 e 4otto riusciresti ad avere molta più chiarezza sulla tua domanda puntuale su quel paziente lì, su quel caso lì ……………………………………………… oltre di competenza personalizzata su quello che ti sta incasinando in questo momento te, sarebbe in realtà un guadagno di tempo e non un'altra procedura da fare con questi qua che magari mi giudicano o una domanda che chissà se la faccio bene, capito? Perché la situazione è questa , ma in realtà l’offerta è assolutamente diversa da questa cosa che viene percepita. E poi l’altra cosa che è formidabile che mi viene in mente adesso parlando con te e che una volta che ………………………..un ragionamento etico ………………………….etica cioè voglio dire alla fine il ……………………. Ti aiuta …………………………………. Una volta che tu ti abitui un po’ a far ragionamenti a quel livello li dopo ti semplificherà anche altre situazioni. Però, secondo me queste 2 robe non sono passate, cioè, ma non sono passate perché nella cronologia della storia di una Azienda è appena iniziato sto servizio.

*Alla luce un po’ di quello che è stato fatto in questi mesi qual è anche una tua valutazione rispetto alle dinamiche che ci sono state in questi mesi, magari dall’inizio dei lavori fino al punto in cui si è arrivati oggi? Sia interne che anche fuori*

Interne secondo me è venuto fuori un po’, adesso non mi viene il termine giusto, però mi sembra che contraddica le cose che ho detto in precedenza, però cioè c’è un po’ d’impreparazione come ….. Cerco di spiegarmi, quando poi affronti veramente nella pratica dei casini che succedono nella ………………………….. con le sfumature che ci sono ecc. ecc. non è facile dare un giudizio armonico e non è facile il confronto e non è neanche facile avere lo stesso linguaggio che non abbiamo perché abbiamo competenze diverse, diversi punti di vista. Quindi secondo me è venuto fuori in questi confronti di quest’anno alle volte un po’ questa difficoltà, cioè che si vede che siamo un comitato per l’etica giovane che è da poco che ragiona insieme che ci sono dei punti di forza nelle tue zone di competenza, ma che nelle altre materie non sei poi così forte, quindi bisognerebbe ascoltarli di più e parlare di meno, però li poi diventa difficile anche per il singolo capire quando è la zona di mia pertinenza e quando no. Manca un po’ di armonizzazione, secondo me ecco su questo comitato per l’etica e credo che questa cosa si vada ad appianare, il linguaggio si vada ad accumunare, coltivando, lavorando insieme,no? Credo anche che forse certe attività formative per il comitato andrebbero fatte, cioè è chiaro che io parlo dal punto di vista delle cure palliative, cioè se tutti sapessero cosa fa il comitato per le cure palliative ospedaliere, sarebbe interessante che tutti lo sapessero sennò nelle discussioni viene fuori che ………………………………….Come forse sarebbe importante fare formazione su altre cose di non mia competenza in modo tale che almeno da una base comune si parte, poi chiaro che c’è il guru di quella materia lì, non so c’è il guru appunto della medicina legale che sarà un livello maggiore rispetto alla medicina legale e quindi non avrà bisogno della formazione su quello, ma magari sarà quello che la fa. Quindi secondo me qualcosina all’interno del CDE, qualcosa di perfettibile c’è.

*Invece dal punto di vista esterno?*

Non so, secondo me bisognerebbe cercare di iniziare a fare dei circoli virtuosi nel senso che non so dei circoli virtuosi li possono creare le consulenze che si fanno …………………………cioè, forse la butto lì, è un idea che mi è venuta in mente adesso, se al ritorno della consulenza da quel medico di base ……………………………………….. se al ritorno fosse dato a tutti i medici di base , faccio un esempio, forse riuscirebbe a creare un circolo virtuoso perché anche chi non si espone riuscirebbe a capire in che termini il comitato si è espresso e quindi magari la volta dopo anche l’altro medico di base ………………………………….. viene a far la domanda, hai capito? Invece se ……………… un dialogo ………………………… cioè il clinico porta il caso e poi ritorna al clinico, magari per i prossimi 20 anni è sempre solo quel clinico lì che li richiede. Perché poi, per esperienza, i motivi di confronto tra i clinici son pochissimi, cioè noi UCP abbiamo un equipe 2 volte a settimana, ma siamo gli unici …... Cioè anche per dire nei reparti ci sono ……… della mattina in cui si parla di tutto, ………. meno che di queste questioni, e quindi lo scambio ………………………………….. che magari ha fatto la domanda al CEC, poi dopo non ha occasione di tornare indietro al suo gruppo e dire, invece se il ritorno, fosse anche non so una occasione formativa per tutto il reparto della Medicina 1, faccio un esempio, si potrebbero creare in questo modo dei circoli virtuosi per cercar di far capire di più cosa è il comitato etico e cosa no è, cioè, capisci? Anche la formazione pubblicitaria del tipo ci siamo ed esistiamo e facciamo questa cosa, va benissimo anche quel tipo di pubblicità, chiamiamola più che formazione, pubblicità, va benissimo, la pubblicità è l’anima del commercio e quindi non prendi niente se non sanno che esisti. Però secondo me per quello che fa, e per farlo comprendere meglio ci va uno sforzo un pochino più organizzato per provar a far capire ai clinici in cosa possano essere aiutati ………………….. Se quel tot di casi di consulenze che sono state chieste al comitato ………………..…………………………….. son più contenti, secondo me, senza che quel clinico si senta giudicato.

*Ci sono degli altri commenti, pensieri o qualcosa che ti è venuto in mente rispetto a questo servizio che ti va di condividere?*

Beh intanto bisogna che ci incontriamo in presenza, secondo me, come comitato per l’etica nella clinica, non so 1 volta l’anno 2 volte l’anno come successo per il Covid, però anche le tavole più assurde di lavoro a cui ho partecipato …………………………………………… perché comunque fa la differenza e sto pensando, ma no, mi sembra di no, il discorso della fisicità delle consulenze o dei nostri pareri adesso a parte il discorso di quei circoli viziosi che ti dicevo ci fosse la modalità di renderli più visibili anche su Intranet, forse anche quello aiuterebbe. Non lo so eh - Intranet è molto visto da tutti, veramente

*Intervistatore*

Secondo me si perché aumenti la comprensione del servizio e poi vabbè sempre pensando all’esperienza del CCP cioè noi ad esempio all’inizio avevamo fatto la formazione su 18 reparti, era stata una formazione a tappeto che però facevamo solo nei reparti che ci chiedevano cosa facessimo, quindi solo una formazione dove la si sentiva l’esigenza …………..….una formazione al gruppo delle altre persone e chiedere un parere lo proporrei sempre ………………. Poi altre cose non me ne vengono in mente, direi che le mie riflessioni te le ho dette tutte .
