# Supplementary material for: How to implement a clinical ethics committee in an oncological research hospital: Qualitative results from a process evaluation study using normalization process theory (EVACEC)
Source: PLoS One. 2025 May 6;20(5):e0318870. doi: 10.1371/journal.pone.0318870 (PMC12054913; doi:10.1371/journal.pone.0318870)
Supplement: S5 File — Interviews transcripts_raw data: this folder contains all the raw data used in this study, the interview transcripts (in original Italian). (ZIP) [file pone.0318870.s005.zip › S5_Interviews transcripts_raw data/04CM.docx]

1. Esperienza con etica

La mia esperienza legata all’etica e alla bioetica risale a quando ho cominciato a far parte del CE nel 2010/2011 in cui ho cominciato ad approcciarmi a questo tipo di lavoro nel quale l’obiettivo principale è quello di valutare dal pdv etico gli studi che vengono presentati al CE. Tra le funzioni del CE c’è anche la discussioni di dilemmi etici nella pratica clinic anche se lo spazio dedicato a questa pratica è ridotta rispetto alla necessità di valutare gli studi dal pdv metodologico , scientifico ed etico. Nonostante questo, dall’inizio di questi temi e ne è parlato ed ho partecipato anche a casi etici nei quali il CE ha aggiunto questa funzione di supporto e consulenza. Chiaro che il CE ha un ruolo istituzionale e formalizzato , questo lavoro è stato impostato dal presidente che allora era….Nel mio primo mandato, il presidente era il Dott. XX, che (*redacted*), prima era presidente del nostro CE. Poi c’è stata la Dott.ssa (*redacted*), poi (*redacted*), poi il Dott. (*redacted*). Io ho partecipato alle varie trasformazioni dei CE e nel corso del tempo, il CE ha avuto diverse riorganizzazioni. Devo dire che ho partecipato ad alcuni casi, ne ricordo uno con la neonatologia, dove in un caso c’era stata necessità di un confronto tra personale medico ed infermieristico su un caso di un neonato che era stato trattato,. Devo dire che è un ambito che mi ha sempre interessato, perché è molto difficile riuscire a leggere nella pratica, correttamente, i temi etici e soprattutto a discuterli e a trattarli in un modo strutturato e utile. Spesso viene fatto in modo spontaneo, casuale, ma credo sia necessario un supporto. E all’interno del CE questo spazio, seppur piccolo e insufficiente, ho potuto vederlo nel corso del tempo.

1. Esperienza con il CEC

Intanto credo che la cosa fondamentale sia il fatto di avere questo organismo dedicato dove sono presenti professionisti che hanno una competenza specifica, e questo per me fa la differenza, nel senso che nel CEC lo spazio è dedicato proprio a discutere di aspetti, di problematiche di casi che abbiano una problematica etica, ma la cosa che ho potuto vedere, e che per me è molto importante, è il fatto che ci sia qualcuno che ha la competenza e gli strumenti per approfondire queste tematiche. Iop ho sentito la necessità di studiare, di approfondire , ma ovviamente ho la percezione…seppur di apportare un contributo legato alla mia pratica – che è quella dell’organizzazione, degli aspetti etici legati agli studi, c’è proprio bisogno di qualcuno che ci guida che ci dà gli strumenti per discutere e trattare i casi dal pdv etico. Questa cosa è fondamentale. Attraverso l’esperienza del CEC quello che secondo me è molto importante è: intanto qualcuno che ha gli strumenti per gestire gli argomenti, poi il confronto multidisciplinare, perché ognuno porta la sua esperienza- dal medico legale, al clinico, al bioeticista, a qualcuno che non conosce la pratica clinica ma che ha una visione più filosofica o più della bioetica, è sicuramente un arricchimento, perché c’è questa contaminazione dei saperi che ci aiuta ad avere uno sguardo a 360 sul problema, che magari potevamo vederne solo una parte, o quello che era legato alla nostra percezione che comunque è sempre parziale.

1. Motivazioni

Proprio perché io credo che….le problematiche etiche che tutti i giorni si vivono nella pratica clinica possono mettere in crisi i professionisti non solo nel trattare i casi clinici ma, me personalmente, quando mi occupo di organizzazione, quando mi occupo di riuscire a trattare gli aspetti dell’organizzazione con equità, considerando tutti i valori messi in campo, cercando di dare delle risposte, in modo appropriato. Problemi che hanno suscitato in me magari dubbi e problemi, Il fatto di poter sviscerare questi aspetti è importante. E poi appunto avendo avuto esperienza - anche se limitata - di problematiche di tipo etico, il fatto di ignorarle e di poterle affrontare con qualcuno che ci aiuta a leggerle meglio secondo me è molto importante per il gruppo, per il singolo professionista, ma anche per dare delle risposte ai pazienti, in generale, più appropriate.

1. Qual è il ruolo del CEC

Nell’azienda, il CEC ha un ruolo fondamentale di orientamento, guida e supporto. Quello che mi piacerebbe far crescere rispetto a questo ruolo…fa parte delle sue funzioni, ma che ho visto, come CEC, ancora in modo parziale, è la formazione degli operatori. Quello che io avverto sempre di più, e in modo ancora più forte, e l’ho fatto anche per gli studi che ho messo in campo, tra cui uno in cui c’erano gli aspetti etici rispetto alla gestione del Covid - Mi rendo conto che i professionisti non sono in grado di tirare fuori i problemi etici, confondono i piani, perché non c’è sufficiente formazione, oppure appiccicano delle etichette di problemi etici a problemi che invece non lo sono. Il fatto di poter avere una formazione, un’ educazione e un confronto con qualcuno che la bioetica la conosce e che ha gli strumenti, per me è stato importante ed è per questo che ritengo che il ruolo del CEC, oltre a esprimere pareri, poter supportare i clinici nella lettura dei casi, sia prima che dopo, - abbiamo visto l’altro giorno: per me è stato veramente un momento molto importante, l’abbiamo anche preparato, il confronto con i professionisti, è stato molto importante dal pdv professionale ma anche personale, credo che oltre a questo vi sia anche la funzione educativa, nei confronti dei professionisti, e perché no, anche dei cittadini (?)

1. La specificità del CEC rispetto ad altri servizi – esempio UDB

Io penso che sono due ruoli vicini ma diversi. La bioetica la vedo più come una consulenza fatta ai professionisti, con i professionisti, dentro la pratica clinica, e quindi di fianco al professionista ed è una consulenza fatta da qualcuno che ha gli strumenti che si mette di fianco al professionista nella pratica quotidiana. Il CEC ha un ruolo più di confronto, più istituzionale, più di confronto multidisciplinare, che si mette in una condizione…non è al fianco, è a supporto del professionista sul caso, però ovviamente c’è una molteplicità di professionisti all’interno per cui è un parere che viene espresso , non vincolante, ma che comunque è a supporto dell’equipe in un modo un po’ diverso. Una domanda al CEC coinvolge un intero gruppo, e magari altri consulenti che possono aiutare su problematiche specifiche, ma che ovviamente ti configurano non come una consulenza fatta ad personam dentro la pratica clinica. E’ vero che è a supporto della pratica anche quella, ma ha un ruolo un pochino più multiprofessionale e trasversale. Sono due aspetti importanti ma leggermente diversi come risposta.

1. Qual e obiettivo e in che modo sta nell’ospedale.

Io penso che il ruolo del CEC sarà sempre più importante. Primo perché ci sono delle tematiche sui cui i professionisti sono chiamati a rispondere, che necessitano di un approfondimento che non c’era …o meglio, ce n’erano meno, perché avevamo più certezze da un certo punto di vista, perché la normativa e la situazione era diversa. Oggi penso alla normativa sul fine vita, penso alle tematiche legate all’inizio della vita (PMA, diagnosi di malattie metaboliche infantili., o sopravvivenza di neonati) che possono porre delle tematiche legate all’appropriatezza delle cure palliative o assistenza parte pediatrica. Quindi fine vita in generale ma non solo: tematiche etiche sono sempre più tematiche che ci riguardano, penso anche al discorso dell’uguaglianza dei diretti delle persone ‘’con genere fluido’’ , che possono mettere il professionista di fronte a problematiche a cui non ci sentiamo pronti. Non soltanto perché sono argomenti difficili, ma soprattutto perché sono argomenti nuovi, innovativi, che non abbiamo avuto il tempo di poter approfondire, ne dal pdv professionale , e neanche in equipe e oggi le risposte non sono mai di un singolo professionista ma di una molteplicità di professionisti che ruotano intorno al pz, e c’è la necessità di una comunicazione e di un supporto dell’equipe importante. Non sempre di fronte ad un pz e ai suoi problemi, c’è un’equipe che ha chiara la direzione e quindi c’è bisogno di qualcuno che ci aiuti ad affrontare, approfondire le tematiche, un po’ anche , con questa visione del CEC che non è mai quella di dare giudizi personali, ma di valutare sempre quali sono le tematiche etiche, distinguendo il livello etico da quello normativo, e soprattutto ognuno per l’aspetto che conosce meglio. Io, anche se non mi ritengo un’esperta di bioetica, ho un’esperienza nell’organizzazione e però mi sento sempre di dare un contributo specifico rispetto ad un ambito che conosco meglio e, il fatto di confrontarsi con altri, che portano il loro contributo, etico o clinico, per me è arricchente e dà una visione più complessiva e utile a tutti.

1. Il suo ruolo nel CEC

Io svolgo il ruolo di (*redacted*) e sono un po’ timorosa (*redacted*) perchè non ho strumenti approfonditi. Sono un’apprendista in corso di apprendimento. La prima volta è stato difficile, penso di aver fatto del mio meglio, però sicuramente ho tanto da imparare ancora. Il mio contributo è legato all’esperienza personale rispetto al ruolo che svolgo, sono (*redacted*) e ho un’esperienza di organizzazione anche in altri dipartimenti, quindi conosco i setting delle professioni sanitarie abbastanza bene, e poi l’altra cosa che svolgo da tanto tempo, è la ricerca e nell’ambito della ricerca ci sono sia aspetti etici e aspetti legati all’eticità degli studi e al fatto di mettere sempre al primo posto il bene del paziente, da questo punto di vista. Lascio fuori la parte metodologica perché ha meno a che fare. Occupandomi di ricerca dal (*redacted*), ho visto tanti risvolti da questo punto di vista. Sicuramente queste due esperienze che ho portato avanti possono contribuire complessivamente. Poi come le dicevo prima è in progress, si impara sempre, e io non ho la competenze specifica. Sono molto sempre in ascolto e soprattutto guidata da chi ha più esperienza e conoscenza di me.

1. Percezione del CEC da arte di professionisti.

Io penso che dobbiamo fare in modo che il CEC venga conosciuto e dobbiamo diffondere il ruolo che può avere e l’aiuto che può dare. Nel momento in cui il professionista tocca con mano l’utilità e l’aiuto che può essere fornito dal CEC, il professionista è contento perché va nella direzione di supportarlo sia come professionista ma aiuta anche secondo me dal pdv personale, perché ti aiuta a mettere a fuoco, quali sono state le motivazioni di un comportamento, quali sono state gli aspetti che hanno messo in crisi il professionista, fanno capire qual è la percezione dell’altro professionista che magari ha messo in campo dei comportamenti professionali che magari erano diversi o magari incomprensibili al momento, quindi secondo me nel momento in cui il professionista capisce quanto può essere utile il CEC non può che accogliere bene questa esperienza. E’ chiaro che nel mare magnum di tutte le cose, bisogna lavorare affinché venga conosciuto e soprattutto dobbiamo fare in modo che il lavoro del CEC sia, come abbiamo fatto fino ad ora, in un tempo utile per le necessità dei professionisti, perché ha senso questo lavoro se è contestuale alla richiesta che viene fatta dai professionisti. Se queste due cose sono coerenti con il bisogno e il tempo necessario per…io penso che sia molto utile. Dopodiché il lavoro del CEC potenzialmente aumenta per i professionisti che ne fanno parte. Penso anche al tema della SMA. Noi sappiamo che è il CE che deve stendere un parere oppure il CEC che può…è comunque un aumento di lavoro.

1. Rispetto alla formazione. Direbbe che i professionisti avvertano questo bisogno di formazione?

No, non c’è ancora questa consapevolezza. Mi è capitato di intervistare alcuni professionisti nell’ambito di quello studio che le dicevo sul Covid, mi hanno tirato fuori dei problemi di tipo etico parlando di problemi relazionali e mi hanno detto sull’aspetto etico delle cose che erano più organizzative che etiche. Alcuni professionisti confondono un po’ i piani. C’è una grande fatica…poi sto generalizzando e le generalizzazioni non vanno mai bene, poi ci sono casi, tipo l’ultimo che ci è capitato, dove il medico è stato molto bravo e preciso a tirare fuori dei problemi di tipo etico. Ma in generale secondo me è difficile che ci sia la consapevolezza di cosa sono problemi etici, dilemmi etici. Queste cose non sono così chiare e consapevoli nella mente dei professionisti. Non è che non hanno idea, è che non sanno tirare fuori dei problemi….lo dico alla luce del fatto che probabilmente nella formazione di professionisti stessi l’etica è all’interno di altri corsi e i formatori stessi non sono formati, non hanno le competenze specifiche..i testi stessi che io ho guardato sono molto teorici, molto generalisti. E’ un ambito molto difficile…non sono consapevoli e questo bisogno esce nel momento in cui qualcuno li aiuta a comprendere meglio quali sono gli aspetti etici di cui tener conto.

1. Questa mancanza di consapevolezza è identica in ogni reparto?

C’è molta differenza a seconda del contesto specifico – casi, problematiche, caratteristiche dei pazienti curati nel reparto stesso. Faccio un esempio: negli anni 80 quando c’è stata la questione dell’hiv, le malattie infettive si sono trovate a doversi confrontare con questi aspetti di tipo etico e sono diventate più sensibili nel tempo. L’allora capo sala delle malattie infettive fece parte del CE proprio perché sentiva questa necessità. Nel tempo hanno sviluppato…Poi dipende dalla sensibilità dei professionisti, dalle esperienze che fanno . E pi dipende anche dalle situazioni contingenti di casi che si possono trovare a gestire, sia dal pdv assistenziale che organizzativo. Quindi no, non c’è la stessa percezione in tutti i reparti e ci sono reparti più sensibili e aperti, altri che non hanno la percezione di questo tipo…

1. Le attività del cec come sono state integrate

Nel contesto locale sono state integrate aggiungendo a chi fa parte…per quanto riguarda i componenti del CEC, sono sia interni che esterni, (*redacted*), quindi aggiungo alla mia attività quella del CEC, quindi è aggiuntiva rispetto a quello che io faccio, extra. Per quanto riguarda, invece, l’attività complessiva nella struttura è stato introdotto attraverso una formazione, per promuoverlo e per promuovere le attività che svolge, che secondo me ha bisogno di trovare ancora una maggiore spazio. Ci sono delle richieste, ma sono contestuali un io’ al periodo forse…nel bene e nel male. Un po’ perché il Covid ci ha messo di fronte a delle problematiche di tipo etico, quindi il CEC ha sentito l’esigenza di scrivere pareri rispetto a vaccinazione e triage e solitudine dei pazienti, e secondo me c0’era bisogno di esprimere queste cose…quello che io non so è se questi pareri, disponibili per tutti, se davvero i professionisti li hanno letti, cosa hanno pensato, quindi c’è bisogno di integrare ulteriormente non solo la diffusione della presenza del CEC nell’azienda, ma c’è da pensare anche a come fare in modo che i professionisti ci restituiscano che cosa ne pensano del materiale messo a disposizione, dei pareri. Abbiamo ricevuto richieste di supporto e consulenza, ma io non so se nei diversi reparti hanno letto…perché i parei sono stati pubblicati nella intranet, ma non abbiamo mai preso in considerazione il ritorno per dire: questo tema: come è stato vissuto? Che cosa ne pensano? È utile? Ha aggiunto un valore positivo? A me interesserebbe molto perché vuol dir che c’è una reciprocità…(..) parlo per le professioni sanitarie, che sono quelle che conosco meglio, ma forse in qualche modo vale anche per i medici.. siamo talmente presi dalla pratica quotidiana per i pazienti che la parte di lettura di articoli di letteratura o di pareri trasversali è casuale, non è sistematica. Non che vengano prima altre cose, ma forse non se ne percepisce l’utilità fino in fondo di questi aspetti, proprio perché non c’è consapevolezza, o si pensa venga prima qualcos’altro. Allora probabilmente c’è bisogno di fare qualche cosa per accrescere…poi magari quando un professionista si mette la e lo legge dice magari l’avessi letto prima, ci sono dei suggerimenti …quindi bisogna secondo me lavorare anche in questa direzione.

1. Limiti

Un limite sono stati gli incontri fatti a distanza, che se da un lato annullano gli impegni, perché si raggiunge ogni luogo e si fa fronte agli impedimenti, perché di fatto è molto flessibile, sono gli incontri a distanza su piattaforma. Che però rappresentano anche un limite perché a volte la connessione non è sempre buona, e si fa fatica a capire il punto, si perdono dei pezzi, si è più distratti perché si è portati a fare qualcos’altro, e ad essere meno attenti ad alcuni passaggi,. La relazione a distanza non è la relazione in presenza: questi aspetti necessitano di un confronto che più è diretto, più è in presenza, più riesce a cogliere gli aspetti importanti che passano attraverso questo confronto. E’ sicuramente positivo perché ha raggiunto tutti, ma dobbiamo tenere in considerazione il limite che non ci vediamo, non ci sono tutti gli altri sensi che nella relazione sono importanti 8la vicinanza delle altre persone). Secondo me è importante nella relazione soprattutto quando ci si confronta su questi aspetti. I limiti sono legati al fatto che questi organismo è stato creato in forma sperimentale, e che i componenti esterni non sono remunerati da questo punto di vista. Quindi magari lo fanno come,,,,si partecipano a questa parte, lo fanno, ci credono, ma non è riconosciuto come attività esterna. Finché è a distanza e posso stare a casa mia, allora, va bene, però secondo me toglie un po’ questa formalità e istituzionalizzazione di questo organismo. Limiti. Nasce per la prima volta e non ci siamo mai confrontati.. sicuramente io ho messo dei limiti personali, forse come me, non avendo mai partecipato al comitato per l’etica nella clinica, è un apprendimento costante, ho sempre cercato di partecipare e non ho mai …perché ci tengo molto ed è un impegno che mi piace rispettare e onorare, però a volte c’è bisogno anche di avere un tempo per studiare, leggere e fare delle cose e non sempre questo…perché non facciamo solo questa cosa. Poi, dal pdv dei professionisti che partecipano, anche la composizione del CEC è nato in modo sperimentale e probabilmente va integrato con altre figure che possono portare altra esperienza, oppure al bisogno sappiamo che le possiamo attivare.

*(casi particolari in cui avrebbe desiderato un esperto?)*

Sicuramente, ne abbiamo parlato anche con (*redacted*), il fatto del MMG (audio interrotto). Il MMG, abbiamo cercato di coinvolgerli: secondo me le cure primarie sono un ambito molto importante, sia perché a domicilio i pazienti vengono assistiti, poi …è un ambito secondo me importante da considerare. Poi rispetto ad altre …mancano forse i neurologi…però la possibilità di attivare al bisogno egli altri professionisti non ha mai fatto sentire la mancanza. L’altro tema secondo me importante, che non è stato stabilito e che invece è, è il fatto delle competenze in bioetica. Cioè all’interno di un CEC una formazione specifica, fatta insieme, tutti i componenti, al di là delle proprie competenze personali che si portano all’interno del CEC, probabilmente, questa forse è una mia esigenza, il fatto di poter condividere una formazione specifica o degli strumenti, ci metterebbe probabilmente di fronte al fatto che tutti conosciamo la metodologia, il metodo, con ciò possiamo discutere i casi. Siamo sempre accompagnati da (*redacted*), e altre persone che sanno, però a volte la chiarezza, o qualche elemento in più secondo me potrebbe aiutarci.

1. Potenzialità

Credo di averne già ampiamente parlato. Formazione per i reparti, secondo me anche un supporto – non dico che lo deve fare il CEC – però anche il supporto a chi insegna nei corsi di laurea delle professioni sanitarie. E’ proprio legato alla competenza etica. Secondo me il CEC potrebbe fornire supporto alla cittadinanza, per esempio…Non perché vogliamo trovare l’etica dappertutto, non è questo, è perché vogliamo saper individuare bene le problematiche senza confondere i piani, saper individuare quando è necessario approfondire questo tema, e quando invece si parla più di temi relazionali, tecnici…poi nella comunicazione c’è sempre una parte etica, non è che ..operò secondo me è importante, un contributo credo sia fondamentale. Se glielo devo sintetizzare in una parola, io non penso che i sistemi sanitari possano andare avanti senza avere un CEC.

1. Valutazione del lavoro dei mesi di attività

Il lavoro è stato organizzato e condotto in modo molto efficace perché chi ha condotto e ha guidato è qualcuno che è esperto, che ha strumenti e che…io mi sono sentita guidata e supportata in questo. Il gruppo secondo me ha all’interno dei professionisti che hanno competenze molto avanzate, probabilmente possiamo integrarci meglio e la formazione che le dicevo prima probabilmente può aiutare ad individuare una linea comune, però ritengo che sia stato progressivamente…però io ritengo che sia per i tempi che per le modalità il lavoro è andato avanti bene e mi sono sentita accompagnata. Certo è che molto lavoro è stato svolto da (*redacted*) e (*redacted*): Gli altri hanno espresso il loro parere, hanno partecipato, ad esempio nella formazione che abbiamo fatto…i clinici hanno portato la loro esperienza e questo mi è piaciuto molto, (…), sicuramente il lavoro progressivamente è migliorato, certo è che è un gruppo che è nato ed è cresciuto nel tempo, che ha scontato un po’ il fatto di non essersi mai incontrato in presenza, questa cosa secondo me si è sentita. Non perché sia mancato il lavoro, ma perché c’è stato dietro un lavoro di regia molto forte, nel quale chi effettivamente ha portato avanti il lavoro è stato il Presidente e la segreteria tecnico-scientifica, poi è vero, con l’aiuto di tutti, ma una grande conduzione.

1. La sua opinione rispetto all’impatto sulla realtà dell’azienda.

Il CEC si sta facendo conoscere e abbiamo ricevuto richieste…forse il periodo che abbiamo vissuto da un lato non ha aiutato, perché non c’è stato il tempo, o comunque ..a volte non si è consapevoli neanche della necessità di avere un CEC di supporto …nonostante questo tempo che ha orientato magari il lavoro…io se penso a me ho aperto una rianimazione di 10 posti letto per 49 gg per il covid e tutti i gg ero li a fare…ci sono un sacco di problemi che mi hanno messo in crisi dal pdv etico e mi sono fatta delle domande, non avevo però il tempo e non ho pensato, pur facendo parte io del CEC, di attivare il CEC su questo tema. O meglio, abbiamo pensato dopo e abbiamo scritto ilo parere…Però questo è quello che ho fatto io, può darsi che altri professionisti non abbiano trovato la motivazione o il tempo per farlo. O in altri casi il fatto di essere entrati in contatto con la formazione, o con l’esperienza di qualcuno che li ha messi al corrente che c’è un CEC ha fatto porre il problema. E’ un lavoro ancora all’inizio, e secondo me bisogna andare avanti sia con la formazione che con la diffusione, essendo consapevoli che questo potrà aumentare solo il lavoro. Dall’altra parte dobbiamo bilanciare le richieste che vengono fatte ai componenti del CEC. Non è che possiamo dire: si si chiamate il CEC poi vediamo, e poi diventa un lavoro a tempo pieno, perché è un lavoro impegnativo.

1. Che cosa funziona di più rispetto alle necessità della realtà locale

La discussione dei casi e il confronto sui casi. Secondo me questo è quello che ha l’impatto sull’equipe e sul singolo. Secondo me dobbiamo fare anche un lavoro di restituzione, perché è chiaro che è il professionista che ci chiede la consulenza che può farlo, noi siamo vincolati al segreto..però se questo diventasse patrimonio dell’equipe e venisse percepito in modo più completo, potrebbe aiutare moltissimo.

1. In che cosa si potrebbe migliorare?

Condividere il percorso di formazione potrebbe aiutarci a migliorare molto, i tempi, la procedura per fare…secondo me qualche incontro in presenza potrebbe aiutarci a condividere. Abbiamo quasi sempre partecipato tutti, non sempre tutti riescono a partecipare, più riusciamo ad avere la voce di tutti i componenti…parlo anche per me, che a volte ascolto molto ma perché ho bisogno di capire e non mi sento sempre all’altezza di poter intervenire…forse aiuterebbe il confronto. Ma questa è una nota…mi sono trovata molto bene nel gruppo e mi sembra ci sia una relazione positiva tra i componenti che favorisce lo scambio. Abbiamo loa fortuna di avere persone del calibro di (*redacted*), (*redacted*), persone che portano contributi moto importanti, oltre ai clinici che portano la loro esperienza, secondo me è molto positivo questo lavoro, l’ho trovato molto arricchente.

1. Commento finale

Mi piacerebbe confrontarmi con altri CEC. Perché in Italia sono a macchia di leopardo e mi piacerebbe molto vedere come funzionano…capisco che ci sono delle differenze, legate ai componenti che fanno parte del gruppo, ovviamente ogni gruppo ha delle caratteristiche specifiche. Operò mi piacerebbe molto confrontarmi con altri CEC di altre regioni per vedere l’approccio che hanno e vedere gli strumenti che utilizzano, per vedere le caratteristiche che mettono in campo e da cui magari possiamo imparare…(….) credo che per le caratteristiche che ha il CEC credo che dovremmo diffondere di più quello che facciamo anche attraverso pubblicazioni scientifiche. Dovremmo diffondere di più questo lavoro anche dal pdv scientifico per fare in modo che non rimanga questa esperienza nei cassetti. Rispetto al confronto con altri CEC ma in particolare rispetto alla nostra esperienza di un CEC che è nato come oggetto di un progetto di ricerca, dobbiamo assolutamente documentare ciò che stiamo facendo perché aiuterebbe noi a non mettere nel cassetto e a farla diventare solo un’esperienza nostra, ma anche a diffondere il CEC.
