# Supplementary material for: How to implement a clinical ethics committee in an oncological research hospital: Qualitative results from a process evaluation study using normalization process theory (EVACEC)
Source: PLoS One. 2025 May 6;20(5):e0318870. doi: 10.1371/journal.pone.0318870 (PMC12054913; doi:10.1371/journal.pone.0318870)
Supplement: S5 File — Interviews transcripts_raw data: this folder contains all the raw data used in this study, the interview transcripts (in original Italian). (ZIP) [file pone.0318870.s005.zip › S5_Interviews transcripts_raw data/01LD.doc]

```
D: 	La prima domanda che le rivolgo è se mi vuole raccontare un po’, dal suo punto di vista, come è stata proprio la parte iniziale. Noi abbiamo già raccolto delle interviste con i componenti del Comitato per l’Etica nella Clinica, con le persone che si sono rivolte a questo comitato, le chiedo invece, dal suo punto di vista, come è stato l’inizio, quali sono state un po’ le tappe che hanno portato alla sua costituzione.
R:	Le tappe… ovviamente il mio è un punto di vista forse un po’ particolare ecco, da questo… perché le tappe sono un po’ nate un po’ in divenire, nel senso che da una parte, come dire, avevamo la possibilità di avere all’interno una professionista come la dottoressa (redacted), che era veramente un’esperta di bioetica e l’avevamo reperita, ovviamente virgolettato, perché l’ambito della bioetica era uno degli ambiti di studio che volevamo sviluppare all’interno delle attività di ricerca degli (? 01:14). Quindi ovviamente temi di bioetica soprattutto in ambito oncologico, il nostro è un IRCCS oncologico, sono temi all’ordine del giorno e quindi di conseguenza c’era una particolare sensibilità; un po’ perché erano temi posti spesso all’attenzione da parte delle unità operative e c’era la necessità di dare in qualche modo la risposta univoca e strutturata ai clinici e dall’altra la necessità in qualche modo, metodologicamente di in parallelo studiare l’approccio a questo tipo di attività, intesa come domanda e come risposta, nel rapporto ovviamente con i clinici e di conseguenza cominciare a sviluppare dei temi di ricerca su questo tipo di attività. Quindi, come sempre facciamo, da una parte avevamo in qualche modo la parte più clinica, istituzionale, a cui si agganciava in maniera molto transnazionale la parte della ricerca; questo è stato un po’ il motivo. Ci sono stati poi anche diciamo sollecitazioni da parte della legislazione rispetto ai temi di bioetica, il problema del biotestamento è che hanno coinvolto anche alcuni progetti di ricerca e quindi in qualche modo l’esigenza è un po’ nata da questo. Un terzo tema, che è legato alla ricerca, in fondo è abbastanza legato al tema che lei poneva e che si voleva fare non solo ricerca sui temi di bioetica, ma anche sull’organizzazione e quindi le risposte rispetto a questo tema, che l’organizzazione poteva dare rispetto ai quesiti. Quindi era anche una ricerca di tipo organizzativo, in qualche modo si poneva anche una ricerca di tipo organizzativo. Quindi di conseguenza in qualche modo questi temi hanno e continuano ad andare molto in parallelo; da una parte, la parte istituzionale ha visto la strutturazione del Comitato per l’Etica Clinica e quindi una strutturazione, come dire, anche con un organismo complesso, perché è un organismo che coinvolge, se non sbaglio sono quindici membri e soprattutto all’interno abbiamo sia soggetti esterni, che soggetti interni. Nell’ambito dei soggetti interni, abbiamo anche una forte interazione fra soggetti molto diversi fra loro; cioè per i medici appunto in servizio all’interno dell’azienda, il personale sanitario. Poi abbiamo il rappresentante dei cittadini, per cui in una logica di empowerment – e questo era anche un altro tema – il rapporto coi cittadini e con l’organizzazione dei cittadini nel come si strutturavano da un punto di vista organizzativo su temi di questo tipo, anche questo sia un filone organizzativo, che un filone di ricerca, ha visto proprio nella strutturazione del Comitato per l’Etica Clinica in qualche modo… cioè vederlo proprio formare insomma e nascere in questo senso. La partecipazione anche di molti soggetti istituzionali del territorio insomma dà proprio il senso della trasversalità, in qualche modo, di questo organismo. E da quelli che sono ovviamente i feedback che ho rispetto a… adesso io ricordo, sono circa due anni che questo… due anni e mezzo quasi che questo Comitato è in funzione e insomma i feedback che abbiamo sono di una forte interazione, sia fra i soggetti che appartengono al Comitato, sia rispetto a coloro che richiedono fondamentalmente una consulenza. L’altro tema è anche il modo con cui può essere richiesta la consulenza, cioè la facilità, l’accedere via internet e richiedere la consulenza; anche questo è un aspetto che è stato molto gradito e quindi il tema dell’accessibilità come strumento organizzativo interattivo è un tema importante. L’altro tema molto importante è anche quello della formazione, perché si è svolta proprio non solo un’attività consulenziale, ma nella logica sempre dell’empowerment, in qualche modo dei professionisti coinvolti, anche un’attività formativa. Quindi, come dire, queste caratteristiche ne hanno a mio avviso determinato proprio il buon funzionamento ma anche l’innesto di una consapevolezza culturale, cioè di una modifica culturale nei professionisti e anche nei cittadini che comunque partecipano, ma molto nei professionisti. Il modello a mio avviso è un modello che funziona e quindi come tale si è cominciato a pensare a un’esportabilità del modello, poi la normativa che sappiamo ha poi, come dire, sta imponendo, tra virgolette, le realtà, anche dinamiche di presenza di Comitati per l’Etica di tipo trasversale. Questo a mio avviso è un bene, le faccio un parallelo, anche se siamo due ambiti diversi, rispetto ai Comitati Etici, che sono nati come Comitati Etici aziendali e poi mi sembra di vedere un po’ un processo simile, comitati Etici aziendali e poi dopo sono diventati Comitati Etici di area vasta, il secondo livello, eccetera. Perché è comunque, come dire, il secondo stadio di vita in qualche modo di questi organismi, nell’ambito del quale a fronte delle prime esigenze, cioè a domanda rispondo ma poi devo rendere facile la richiesta e alla formazione, come dire, svolgo anche attività di ricerca come abbiamo detto prima e attività formativa, si impone un terzo tema che è quello della in qualche modo armonia, cioè in un ambito così delicato un’omogeneità, cioè il tema anche dell’omogeneità rispetto alle risposte, rispetto ai quesiti diventa un tema di governance, che è una governance a questo punto tendenzialmente di livello regionale. Quindi si passa ovviamente attraverso le aree vaste ma poi di livello regionale, perché l’omogeneità di approcci è effettivamente nell’ambito organizzativo un’esigenza più regionale; mentre la richiesta di avere appunto delle risposte e in qualche modo attivare un servizio funzionale al professionista è un’esigenza tendenzialmente più aziendale. Ma quest’ultima esigenza potrebbe cozzare, tra virgolette, con aspetti di uniformità che invece sovrintendono a livello di governance regionale.
D:	Mamma mia, la ringrazio, è stata veramente… mi ha dato delle informazioni utilissime in questo ultimo veramente prezioso, un punto di vista veramente… la ringrazio.
R:	Niente.
D:	Le faccio un’altra domanda, un po’ legata a questo aspetto anche di differenziazione un po’, che mi stava dicendo, del servizio. In che cosa, secondo lei, un po’ mi ha detto, quindi la formazione, l’esigenza di dare delle risposte sui temi di bioetica; in che cosa secondo lei si distingue se c’è una specificità di questo Comitato per l’Etica nella Clinica, che lei vede?
R:	Intanto la trasversalità dell’approccio. È nato, come dire, la trasversalità dell’approccio che si vede anche nella trasversalità delle competenze dei componenti. E questo secondo me è un valore aggiunto, nel senso che se era stato inizialmente pensato, nato diciamo così da un’esigenza prima di tutto legata all’ambito oncologico, pensiamo al fine vita, ma dall’alta parte poi era stato affiancato a un’esigenza che veniva da un altro settore, cioè l’ambito pediatrico e quindi in qualche modo… che erano un po’ i due ambiti abbastanza più pressanti, ha visto quindi nella modalità di approccio nei confronti dei servizi questa trasversalità e quindi la capacità di affrontare il tema di fine vita, per esempio, è un esempio naturalmente, in un contesto oncologico, il tema delle cure palliative sul quale c’era dato l’IRCCS, dato il modo con cui si veniva a strutturare ormai l’IRCCS, una maggiore maturità. Dall’altra parte invece l’urgenza posta dal settore pediatrico ha cominciato ad avere… che era legata magari a singoli eventi, singoli casi ma estremamente delicati, ha visto piano piano anche qui una empowerment dei professionisti della pediatria; qui ho visto proprio questo effetto, dei professionisti della pediatria in generale, del materno, infantile, di quell’area lì, e quindi qui l’aspetto informativo ha inciso molto, si è spostato verso la capacità di dare una risposta non solo ai singoli casi, ma una risposta organizzata e professionale a temi che si possono presentare e quindi anche a un approccio più trasversale. C’è un altro tema secondo me che, come dire, si caratterizza dal fatto che ci sono molti temi che hanno coinvolto l’area delle professioni sanitarie e questo è un altro aspetto che va verso quello che dicevamo e cioè un approccio culturale e quindi l’ambito delle professioni sanitarie con la sua trasversalità è stato fortemente coinvolto anche nei temi proposti, ma non soli nei temi proposti, ma anche per esempio nel coinvolgimento alle volte di questo Comitato, non solo con un ambito consulenziale, diciamo di consulenza sui generis, presente nella […] sì, facevo un esempio; cioè le professioni sanitarie hanno coinvolto questo comitato con i componenti non solo in un ambito consulenziale legato al caso concreto, ma anche – e questo è il passaggio importante – e questo non l’hanno fatto solo loro devo dire la verità, perché anche la pediatria l’ha fatto, ma hanno cominciato loro e sono insomma state un po’ caratterizzanti rispetto a questo, ma anche per esempio coinvolti nella costruzione di procedure, ma anche di PDTA, quindi si è passati – e questo è un salto di qualità – nell’essere coinvolti nel momento in cui tu vai a costruire dei percorsi di cura, di assistenza e quindi in un ambito assolutamente progettuale. Questo a mio avviso è la cartina di tornasole di un salto culturale e quindi un po’ c’è questo aspetto. E poi un ultimo aspetto, questo ovviamente sempre dal mio punto di vista, che guarda anche un pochino chiamiamola alla ricerca epidemiologica e questo per esempio so che ci sono stati, come dire, diversi… un po’ perché all’interno se non sbaglio dei componenti comunque c’è un epidemiologo che fa ricerca e quindi anche una serie appunto di ricerche rispetto per esempio al biotestamento, quindi a quell’aspetto che riguarda un pochino chiamiamolo così l’approccio che è sempre un approccio di cura, ma un approccio rivolto non solo al percorso di cura ma anche alle caratteristiche della popolazione rispetto a una serie di azioni che toccano il Comitato per l’Etica. Ecco, questo è l’altro aspetto, quindi direi una forte trasversalità.
D:	Ho capito. E ripensando un po’ alla sua esperienza, al suo coinvolgimento, anche all’inizio; quali sono secondo lei gli step, i punti fondamentali per implementare un servizio di questo tipo? Oltre al fatto, alla professionista… […] stavo dicendo quali sono secondo lei i punti necessari per poter implementare questo servizio all’interno di un’azienda sanitaria? Prego, grazie.
R:	Allora, intanto ovviamente prima di tutto la presenza di competenze, cioè di persone, nel caso di specie, appunto abbiamo una professionista, la dottoressa (redacted) che è una persona particolarmente competente, è una ricercatrice competente in ambito di bioetica. […] E quindi per competenze, ma non bastano, perché dall’altra parte a mio avviso, nel nostro caso c’è stata una particolare sensibilità del contesto organizzativo di riferimento, rappresentata dall’avere l’IRCCS all’interno, nell’ambito del quale si volevano sviluppare ambiti di ricerca in ambito… cioè nella bioetica. E quindi questo è stato un po’ il la, diciamo però questa è una caratteristica che potrebbe essere in altri contesti organizzativi non ripetibile; nel nostro caso l’esigenza non è sorta da un dovere rispondere a una sollecitazione normativa, ma è stata proprio quella di volere implementare un ambito di ricerca. Come sempre capita, cioè come capita nell’ambito del nostro IRCCS o anche unirsi in ambito di modelli assistenziali, come vi dicevo prima la ricerca… diciamo una prima fase di ricerca, punto, si è passati invece a un secondo step di implementazione in qualche modo, anche un po’ sulla base di quelli che erano un po’ gli esiti appunto e l’esperienza che si era fatta nell’ambito di ricerca, al volere strutturare un organismo, quindi darsi un’organizzazione che rispondesse in maniera strutturata e quindi non solo nell’ambito di progetti di ricerca, perché (? 19:21) con progetti a offrire, uso il termine servizi, ma è improprio, ma tanto per capirci, nell’ambito di progetti di ricerca. Come spesso capita in un ambito di ricerca, si parte con un progetto o vari progetti, quello che è l’esito positivo del progetto diventa qualcosa di strutturato. Questo è stato il passaggio, l’esito positivo ci ha determinato a strutturare invece un organismo. Io mi rendo conto che forse questo è qualcosa di difficile ripetibilità, però sarebbe auspicabile, perché la ricerca evidenzia in qualche modo, ti fa vedere quella che è la domanda e l’offerta, non so come dire, e quindi è una sorta di prima prova; dopodiché ti permette quindi di dire, di focalizzare – questo è il termine giusto – di cosa hai bisogno. E allora questo punto lo strutturi. Questo vuol dire che nel momento in cui tu lo strutturi, c’è anche da parte dell’istituzione dell’azienda, non la volontà di organizzare perché lo dice la norma, non so come dire, ma di strutturare quell’istituzione, quell’organismo perché ne ho sentito la necessità, perché risponde a una domanda. È questo secondo un passaggio importante, la ricerca è uno strumento che permette di farti capire di cosa hai bisogno, in che modo, e quindi è una sorta di prova generale, che ti permette poi di farti un’idea di come puoi strutturarla. Nel momento in cui la strutturi lo fai perché ci credi, lo fai perché stai rispondendo a un’esigenza che si è evidenziata e che anche una risposta strutturata, che quindi non deve essere legata solo al singolo progetto ma deve strutturarsi. Io penso che questo è stato il nostro percorso e questo potrebbe essere il percorso ottimale proprio per dare valore, nel senso perché ci credo ecco, fondamentalmente, e credendoci ovviamente è uno strumento che poi usi perché ne hai il bisogno e perché te lo sei strutturato in funzione delle tue esigenze, o meglio quelle dei tuoi professionisti. Questo penso sia il modo migliore. E quindi potrebbe essere questo il modo migliore per farlo anche a livello macro, attraverso percorsi di ricerca in rete fra più strutture. Cioè io il modello che si è determinato qui e questa esperienza penso che sia replicabile, ovviamente con ricerche multicentriche, quindi che magari possono avere focus diversi rispetto ai nostri, però… tra l’altro la ricerca un po’ evidenzia anche le differenze e le similitudini fra le varie strutture, quindi aiuta anche a strutturare poi un organismo che nel momento in cui viene strutturato contempera, mediandole anche, le varie esigenze in modo che l’armonizzazione non sia imposta, ma sia bottom/up.
D:	Chiaro. E secondo lei questi, mi pare di capire, siano anche dei punti di forza, pensando un po’ all’esperienza e alle future aperture e futuri rilanci; le chiedo se invece lei ha riscontrato in questo percorso, sia all’inizio ma anche in questi due anni e mezzo di attività dei limiti, qualche criticità.
R:	La criticità penso venga in realtà dalla… cioè non la criticità ma quanto la caratteristica, ci vuole molta energia, fondamentalmente, per… cioè è un percorso di accreditamento e di credibilità, che deve essere esercitato con convinzione (? 23:53) i componenti. Ecco, questo secondo me è un aspetto di forza ma anche di debolezza; quindi la capacità di dare delle risposte e di potere, in realtà da parte dei servizi facilmente; cioè devi metterti nelle condizioni di offrire tra virgolette ai servizi un accesso facile, ma anche dare loro una risposta che in qualche modo li accompagni non soltanto alla risposta del caso concreto, ma come dicevo a dotarsi di strumenti nel loro agire quotidiano. Quindi secondo me è un punto di forza e di debolezza allo stesso punto; in questo senso dico di debolezza, ci vuole una grande volontà nei componenti e una grande competenza e mi viene da dire anche deve essere un gruppo coeso, quello dei componenti, perché la risposta deve essere una risposta veramente multiprofessionale, quindi quel doversi accreditare è il percorso di accreditamento della propria credibilità è un percorso non semplice e secondo me non trascende dalle caratteristiche dei singoli. Quindi questo non funziona a prescindere, perché abbiamo creato il Comitato, i componenti del Comitato fanno la differenza; questo è un punto anche di debolezza, se si vuole, ecco.
D:	Ho capito. E accreditamento le chiedo se può essere inteso anche come forma di integrazione, cioè di…
R:	Assolutamente sì. Integrazione sì, perché quando un servizio o un dipartimento mi chiede di partecipare nella creazione del PDTA vuol dire che io mi sono accreditato.
D:	Okay, quindi ecco da questo punto di vista, un po’ anche con le informazioni che mi ha dato prima, secondo lei c’è stata questa integrazione.
R:	Secondo me sì. E si potrebbe pensare di misurarla, tra virgolette ecco, quindi non soltanto sulla base di episodi, però secondo me potrebbero esserci degli indicatori, uno di quelli che le dicevo potrebbe essere un indicatore. Cioè potrebbe essere utile dotare in qualche modo il lavoro attraverso un sistema di indicatori, chiamiamoli indicatori di gradimento a medio-lungo periodo ecco, feedback rispetto agli esiti dell’attività consulenziale che si è fatta. Ecco, quello potrebbe essere secondo me un buon sistema.
D:	Molto utile, molto interessante. Le faccio un’ultima domanda, stavo un po’ guardando anche i vari punti che volevo affrontare con lei, qual è il suo ruolo in questo momento, per questo servizio?
R:	Allora, sicuramente nell’incipit ho svolto un ruolo, nel senso che ci abbiamo creduto proprio in questo passaggio. Il mio ruolo è come Direttore operativo degli IRCCS, cioè continua a essere un ruolo, in qualche modo è un ruolo di stakeholder quasi, di stakeholder interessato, nel senso che l’interesse è quello a che il Comitato e i loro componenti continuino questa attività perché ne abbiamo effetti positivi, come le dicevo, anche proprio nelle procedure dei servizi stessi, e nell’altro perché continui questa natura di assistenza e ricerca, in parallelo, perché per noi come IRCCS ovviamente, come IRCCS abbiamo tutto l’interesse, essendo IRCCS, anche ai modelli assistenziali e questo è un tipico esempio a mio avviso di ricerca sul modello assistenziale e su un percorso di cura; cioè è comunque sul modello assistenziale, quindi di conseguenza… e tra l’altro la ricerca sanitaria sui modelli assistenziali è una ricerca difficile, una ricerca difficile da farsi, la fanno in pochi, ma se penso anche a livello regionale penso che siamo tra i pochi a farla e penso anche che potremmo guadagnarci, ed è questo un po’ l’obiettivo in lungo periodo, medio-lungo periodo, di ritagliarci anche con un’attività del Comitato di questo tipo, cioè (? 29:03), diventare un po’ Hub rispetto ad alcuni temi sui modelli assistenziali, rispetto al livello regionale, sia come meccanismo, sia anche come ricerca, in parallelo ricerca sull’attività che svolge. E quindi è un po’ questo ecco, sono un po’ uno stakeholder interessato diciamo così, che cerca, diciamo aggancia le risorse del budget della ricerca per permettere ai componenti del Comitato di continuare a fare ricerca. Il mio ruolo è questo, devo dire il mio scopo e il mio obiettivo insomma. Trascendendo dal livello regionale, adesso siamo un passaggio importante, cioè che è quello dal livello aziendale a un passaggio verso il livello più macro, nel quale l’idea è quella di accreditarci anche come IRCCS, visto che IRCCS è in modelli assistenziali, nei confronti del livello regionale. Affinché ci possa magari un domani commissionare della ricerca che diventa una ricerca in rete su implementazioni di modelli di questo tipo.
D:	Molto interessante quello che diceva, appunto di diventare un Hub anche rispetto a determinate tematiche, mi viene in mente insomma il nuovo decreto anche, riguardante il suicidio medicalmente assistito, questi aspetti qua, per i quali c’è stato una forte discussione, che è tuttora in essere, quindi insomma, anche avere queste esperienze, queste progettualità è sicuramente utile per avere anche qualcosa di concreto su cui ragionare.
R:	Proprio così.
D:	Perfetto. Dunque, io non le voglio rubare ulteriore tempo, perché siamo quasi in scadenza anche del tempo, le chiedo però in conclusione, mi ha raccontato tantissime cose e mi ha dato delle informazioni veramente utilissime e preziosissime, le chiedo se rispetto a quello di cui abbiamo discusso questa mattina, se ci sono degli altri elementi che lei ha piacere di condividere rispetto a questo CEC, a questo Comitato per l’Etica nella Clinica, qualcosa che magari non è ancora emerso e che rispetto alla sua esperienza invece ritiene sia utile, sia importante.
R:	Forse il tema del rapporto con i cittadini, potrebbe essere un tema ancora… non perché non sia stato considerato il fatto… infatti l’esempio all’interno del Comitato per l’Etica abbiamo un rappresentante dei cittadini, però penso che sia, come dire, un tema nel quale qualche cosa debba essere fatto, o su un’attività di formazione/empowerment del cittadino, penso un po’ a diverse linee del PNRR sull’M6, cioè su queste linee di empowerment dei cittadini, in forma singola organizzata, penso che questo aspetto che credo è iniziato, non è… però potrebbe essere in qualche modo qualche cosa che viene affiancato, in più collaborazione con varie associazioni che abbiamo all’interno, con le quali costantemente collaboriamo per condividere il tema non a seguito di li chiamo articoli di cronaca, ma per cominciare a parlare di questo tema, che è un tema delicato, non più in maniera sensazionalistica ecco, non so come dire, e quindi cominciare a introdurre una cultura, come è avvenuto, come sta ancora avvenendo per i professionisti, anche con i cittadini, partendo ovviamente da quelle che sono le associazioni più vicine a noi; penso in ambito pediatrico, come già sta avvenendo e in ambito oncologico ecco, ma non solo, penso anche per esempio all’ambito del fine vita legato alle persone anziane e quindi dove ci sono molte associazioni che partecipano insomma insieme a noi e ci aiutano. E penso anche per esempio che qualcos’altro potrebbe essere svolto rispetto al tema della domiciliarità, cioè l’altro ambito è il D.M. 77, e quindi che vede uno spostamento di attenzione molto forte verso il territorio, verso quella che è l’offerta che dovrà essere data nel rapporto ospedale/territorio. Ecco, io lì vedo degli ambiti che potrebbero essere esplorati ecco, devo dire la verità, esplorati, sì.
D:	Benissimo, li esploreremo, cercheremo di… no, no, sulla base di questi rilanci sicuramente appunto il Comitato si organizzerà di conseguenza; adesso staremo anche un po’ a vedere che cosa succede, anche a livello un po’ legislativo, se ci saranno delle novità in questo senso, però intanto ecco, mettere a punto quali sono i passaggi fondamentali, affinché un servizio di questo tipo funzioni, sia efficace all’interno del suo contesto è sicuramente un primo punto da cui partire, anche per poi riprodurlo fuori. Quindi io la ringrazio veramente ancora per la disponibilità, per insomma tutto quello che mi ha detto e direi di chiudere qui, chiudo la registrazione.
Fine trascrizione 00:35:33
```
