# Supplementary material for: How to implement a clinical ethics committee in an oncological research hospital: Qualitative results from a process evaluation study using normalization process theory (EVACEC)
Source: PLoS One. 2025 May 6;20(5):e0318870. doi: 10.1371/journal.pone.0318870 (PMC12054913; doi:10.1371/journal.pone.0318870)
Supplement: S5 File — Interviews transcripts_raw data: this folder contains all the raw data used in this study, the interview transcripts (in original Italian). (ZIP) [file pone.0318870.s005.zip › S5_Interviews transcripts_raw data/02LD.doc]

```
D: 	Dunque, la prima domanda appunto che le volevo un po’ chiedere è una domanda generale, su come è stato così un po’ il processo all’inizio, se vuole raccontarmi com’è iniziato un po’ questo percorso, anche di progettazione e di attivazione di questo servizio.
R:	Allora, il tutto nasce devo dire da una ricchezza che questa azienda aveva, che è quella di avere appunto una professionista come la dottoressa (redacted) all’interno del nostro IRCCS; quindi diciamo che siamo partiti molto favoriti in questo processo. Noi siamo IRCCS oncologico dal 2011, dal 2011 sono attive dentro l'ospedale Santa Maria Nuova, che allora era azienda ospedaliera, quindi era un'azienda separata rispetto all'azienda ULSS nella quale io ho sempre lavorato, ci siamo fusi il 30 giugno, 1° luglio del 2015. Quindi dicevamo, mentre l’azienda territorio dagli anni ’80 lavorava ad esempio sullo sviluppo delle cure palliative, quindi comunque aveva invitato ad affrontare tutto il problema ad esempio del fine vita, insomma problemi che attengono anche alla bioetica, l'azienda ospedaliera nel momento in cui è nato l’IRCCS si è dotata di unità di cure palliative ed anche di unità di bioetica; adesso non le so dire in che anno è arrivata l'unità di bioetica; comunque insomma diciamo che nasce all'interno dell’IRCCS, quindi della vecchia azienda ospedaliera e matura all'interno, dopo la fusione di un'azienda molto grande, quindi con tante altre sfaccettature, che non è solo quella della parte ospedaliera. Quindi oltretutto la fusione ha portato anche ad un grande rafforzamento della medicina legale, tutto questo ha un po’ coinciso con l'uscita della Legge Gelli, quindi c'è stato tutto diciamo una collaborazione molto importante, spontanea; ecco diciamo questo, la cosa che mi ha colpito di più in questo processo è che il tutto è nato dalla base insomma, è nato buttom/up e non top/down. Quindi è nato dai professionisti, per professionisti intendiamo i professionisti che si occupano di cure palliative all’interno dell’IRCCS, la medicina legale che si è occupata dell'applicazione della Legge Gelli, le cure palliative territoriali. Insomma diciamo che in questo contesto ha creato un po’ l’humus giusto affinché dalla base emergesse questa idea, supportata anche dal fatto che essendo anche un’azienda territoriale noi ci siamo dovuti confrontare negli anni con tanti casi, su come gestire un fine vita che non è quello classico a cui siamo abituati, le persone che non sono più coscienti e quindi devono in qualche modo avere qualcuno che decide per loro. Ad esempio, noi ci siamo trovati con dei minorenni, molte volte, con dei minori con dei problemi enormi, patologie croniche emotive, con problemi di fine vita anche molto lunghi, che potevano questioni importanti di bioetica, ma lo stesso si può dire anche per gli anziani ad esempio ricoverati nelle RSA. Quindi insomma è nato soprattutto da loro comunque, che più volte interpellata, anche la (redacted), su dei casi specifici, penso che abbia dato loro un po’ l'occasione per dire perché non facciamo qualcosa che non è il Comitato Etico, che stesso ci si rivolgeva in modo non corretto al Comitato Etico, che ha un’altra finalità, un’altra mentalità, un’altra formazione, che si occupa soprattutto di sperimentazioni. Mentre invece un Comitato - infatti l’abbiamo chiamato per l’Etica e non Comitato Etico - per l’Etica è stata una… insomma un’importante innovazione che è nata soprattutto dalla loro volontà, dalla loro voglia, della loro competenza e da esigenze che via via sono emerse nel territorio. La Legge Gelli devo dire ci ha dato un po’ una mano su questo, sul far emergere il valore di quello che avevamo in casa e quindi… e le dirò di più, che quando è stato deliberato, chi è rimasto ha fuori sotto il diavolo quando perché ci voleva essere e mi riferisco ad esempio alla medicina generale, che in prima battuta non era stato contemplata all'interno del comitato per l’etica clinica. Quindi a testimoniata anche di una Provincia molto sensibile a queste tematiche, comunque tutti gli attori ci volevano essere; quindi questo è un po'…. Dal punto di vista della direzione, non abbiamo potuto che apprezzare l'idea e quindi sostenerla, come sempre accade devo dire, quando l'idea nascono dai… le idee valide nascono dai professionisti, hanno ancora maggior valore, perché per noi è una delle cose più difficili, quando hai un’idea trascinarti dietro gli operatori, se sono gli operatori che trascinano noi è più facile. E qui di fatto è andata così.
D:	Ok, molto chiaro.  E rispetto a quest'ultimo punto di cui mi ha parlato un po' no di questa anche volontà da parte di altri attori dell'azienda di esserci, mi vuole raccontare come è stato un po' la costruzione proprio di questo Comitato all'inizio, quali sono stati i passaggi secondo lei fondamentali per metterlo in piedi, fondamentalmente; al di là punto di quello che mi ha raccontato, dell’emergere di una…
R:	Allora, sono un po’ impreparata, nel senso che quando le dico nata BOTTOM UP è una cosa che i professionisti hanno elaborato loro, cioè non è che sono venuti e mi hanno detto cosa ne dici se facciamo un Comitato per l’Etica Clinica? Incominciamo a lavorarci? Loro prima ci hanno lavorato, poi mi hanno detto noi saremmo pronti, la direzione cosa dice? A quel punto la direzione, come dicevo, era solo che contenta; quindi in realtà come si è costruito è più da chiedere a loro che non a noi. Quando si è trattato invece di parlare con le persone che volevano esserci e in quel momento non c’erano, abbiamo semplicemente detto, signori il gruppo si può sempre ampliare, vediamo come funziona e poi dopo ovviamente si può aprire ad altre realtà. Io so che adesso anche da altre realtà provinciali e regionali ci chiedono insomma poi di poter percorrere la nostra stessa strada, ma devo dire è proprio uno dei campi più bottom/up che ci siano. Poi (redacted) fa molta ricerca, cioè è anche una persona un po' diciamo vulcanica per certi versi, quindi insomma è molto brava a trascinare un po' le folle e a trasmettere entusiasmo; quindi direi che è soprattutto lei il cuore di questa attività. Noi direzione siamo stati ben felici, attori un po’ più passivi; se dovessi dire che era un’idea mia, no. Però ne sentivo l’esigenza eh,  perché soprattutto quando facevo altri mestieri, cure primarie, Direttore di distretto, così alla volte le questioni proprio etiche non sapevamo a chi farle presenti, con chi confrontarci, quindi indubbiamente è una grande ricchezza questa.
D:	Ho capito, è interessante anche questo punto di vista, il fatto di… poi non so se magari questo CEC, per come è… CEC è l’acronimo per Comitato per l’Etica nella Clinica, quindi ogni tanto uso questo acronimo, se magari anche dal suo punto di vista può essere a livello anche di direzione magari, no? Può essere comunque percepito come un supporto. Non lo so, questa è una domanda che mi è appena venuta in mente.
R:	Allora, è soprattutto un supporto per i professionisti, per chi è sul campo; poi è chiaro che chi è in direzione, cioè a mano a mano che il problema si fa più grande sale. Ad esempio, noi adesso vediamo il caso molto complesso di una ragazzina minore, con una famiglia molto complicato, è chiaro che viene… in questo momento, quando ero io Direttore sanitario, più volte sono dovuta intervenire in questa situazione, oggi che va beh, io non sono più Direttore sanitario, ma l’attuale Direttrice sanitaria è lei che gestisce questa situazione; quindi avere il Comitato per l’Etica Clinica è chiaro che per noi, che non siamo dei clinici, soprattutto, che non siamo dei clinici, è un supporto enorme; lo è per i clinici, ma a maggior ragione, secondo me, lo è per chi non è più un clinico. Perché sono scelte su cui la bioeticista ti mostra la realtà che tu vedi, te la legge in un modo per cui arrivi poi alle decisioni in modo più sereno e consapevole, ecco, questo sicuramente sì.
D:	Ho capito. Immagino che quindi anche avere un gruppo comunque multidisciplinare, perché tra l'altro questo CEC è composto da…
R:	Sì, certo, certo, multidisciplinare, perché le decisioni non le può mica prendere una persona sola, soprattutto decisioni di questo calibro. Adesso, mi dispiace, non sono molto, ma io faccio il (redacted) di una struttura (? 10:39) con 7.600 dipendenti, 800 convenzionati, problemi molto più prosaici tutti i giorni e quindi non… diciamo che poi quando le cose vanno bene, la direzione è quella meno coinvolta, perché vuol dire che le cose vanno avanti da sole insomma. Quindi noi siamo stati coinvolti nel momento dell’istituzione e poi insomma abbiamo delle informazioni, parliamo di budget, però non è… ecco, la cosa importante che abbiamo fatto è stato quello di spostare l’unità operativa sotto la medicina legale, responsabilità professionale, che dipende direttamente dalla Direzione sanitaria, quindi è proprio un’unità operativa incardinata nella Direzione sanitaria. E anche questo avvalora il fatto che il Comitato per l’Etica Clinica è al servizio dei professionisti ma anche della direzione; il fatto di stare lì ha un significato importante. Lavora molto con Giorgio Gualandri, è il responsabile direttorio della medicina legale, responsabilità professionale e anche questo non lo so insomma se è così dappertutto, sinceramente non me ne sono occupata, (? 11:58).
D:	Ė un bel punto di domanda, nel senso che come le dicevo prima non ci sono esperienze di questo tipo, cioè sono molto spontanee un po', è difficile avere anche un modello in base a cui dire ok, questo funziona, funziona in questo modo oppure no. Dunque, le chiedo un attimino se a livello di processo diciamo così ci sono delle situazioni che lei ritiene un po' ostacolanti di questo servizio.
R:	No, sinceramente no, anzi, secondo me fanno fatica, faranno fatica, il gruppo, che fa anche tante altre cose a… insomma, ad assecondare tutte le richieste di consulenza e di collaborazione che ha. No, ostacolanti direi che sinceramente non… almeno io non ne ho viste, o comunque non mi sono state riportate. Proprio perché dicevo è una Provincia che ha investito su questi temi, pur in modo più settoriale, come quello di cure palliative, da 40 anni; piano piano loro sì che erano una roba, negli anni ’80 era veramente qualcosa di strano, oggi no, sinceramente non ne ho mai viste. Poi può essere che (redacted) le vede e io non lo sappia.
D:	Quindi anche a livello un po’ dì elementi che invece hanno facilitato il processo, continuano diciamo così a facilitare il processo ed anche l'integrazione di questo Comitato all'interno dell'azienda? Rispetto un po' a quello che mi ha detto prima, cioè delle…
R:	La cultura, cioè non sono cose che si improvvisano queste, non sono cose che si possono imporre, secondo me sono figlie di una cultura che si è sviluppata negli anni. E insomma, anche… adesso io parlo sempre di cure palliative perché è una delle cose che conosco meglio, perché ho, voglio dire, quando lavoravo alle cure primarie ho avuto grandi occasioni di lavorare per palliativisti e sono sempre rimasta affascinata dall’apertura che hanno queste persone grazie alla formazione continua che fanno, proprio cambia le teste. Un giorno parlavo con una collega, medico di medicina generale, palliativista, lei mi disse io non sarei il medico di famiglia che sono se non fossi anche un palliativista; proprio perché entri in dinamiche, la comprensione dell’altro molto diverse e molto più profonde rispetto a un medico qualunque. Quindi diciamo che questa è una cultura che è nata da pochi, poi piano piano si è espansa, ha colonizzato l’ospedale, che l’ospedale è sempre più duro a farsi colonizzare, però è riuscito a colonizzare forse anche alla presenza della dottoressa Tanzi, eccetera e ha prenderne atto un po’ tutti, compresi quelli che stanno in territorio. Abbiamo esperienze importanti anche nelle RSA, quindi insomma direi che è stato forse questo l’elemento (? 15:29). Di ostacoli devo dire la verità non ne ho visti.
D:	E secondo lei ci sono dei potenziali, aldilà del processo che c’è stato prima, pensando anche ad una futura evoluzione di questo servizio, ci possono essere ipoteticamente anche delle cose che possono inficiare il servizio oppure ostacolarlo, ostacolarne l’integrazione all’interno dell’azienda?
R:	Mah, sinceramente non ne vedo; non ne vedo perché ormai è talmente tanto parte integrante di questa azienda… poi è vero che cambiano le persone, cambia, potrebbe anche cambiare il panorama, non vedo un cambiamento a breve termine, in senso peggiorativo anzi. Non lo vedo, poi io dico magari (redacted) li vede e io no, perché io sono in una posizione un po’ particolare, nel senso che sono ricettacoli di problemi; quando i problemi non mi arrivano vuol dire che le cose vanno bene. Insomma, non ho neanche sentore, poi è chiaro che ci può esser sempre il personaggio che viene, dice, sono tutte cazzate, questo lo possiamo far cadere, però si tratterebbe di un elemento da isolare. In questo momento non ne vedo; poi dico, magari sono… anche perché sono un’inguaribile ottimista, potrebbe essere anche questo.
D:	Poi mi ha molto colpito quello che lei mi ha detto del passaggio sotto la medicina legale, quindi anche di questo riconoscimento a livello istituzionale dell'unità operativa e anche del CEC, io credo che sia…
R:	Sì, di essere proprio una parte integrante della direzione. Adesso magari forse bisognerebbe sentire un po’ la dottoressa (? 17:23) che magari, non lo so se ha un appuntamento anche con lei, la Direttrice sanitaria, perché direttamente dipendente dalla Direzione sanitaria. Cioè, dal mio punto di vista ponti d’oro e grandi soddisfazioni e la direzione ovviamente è sempre disponibile a rimuovere eventuali ostacoli che si dovessero proporre. Ma insomma in questo momento, ribadisco, abbiamo tanti problemi, per fortuna non questo.
D:	Finché tutto tace, della serie, va tutto…
R:	Sì, lo negherà, sei un po’ superficiale, dovresti andare a sentire, però ha detto tutti gli anni ci vediamo al budget e insomma, c’è un’attività che viene rendicontata, anche raccontata insomma, sempre molto positiva. Per quel che è un lusso, allora forse qualcuno lo può considerare che è un lusso avere un’unità di bioetica, per noi è diventata prima necessità, ne vediamo proprio la estrema utilità, non ci rinunceremo mai, mentre ci sono aziende che non ce l’hanno e vivono lo stesso; però per noi ce l’abbiamo, è un nostro punto distintivo importante.
D:	Ho capito. Va bene, allora… dunque, sto riguardando un attimo le domande che avevo predisposto, per vedere se magari mi è sfuggito qualcosa, però direi che mi ha risposto fondamentalmente a tutto; cioè a tutti i punti che volevo un attimo toccare, le chiedo in chiusura se magari ha qualche suggerimento ecco, per migliorare questo servizio in generale, se c'è qualcosa che le viene in mente, che può essere attuato a livello migliorativo.
R:	Mah, adesso, lo ripeto, faccio un po’ fatica a vedere… diciamo che forse, una cosa che potrebbe essere utile, ma magari la fanno già e io non lo so eh, potrebbe essere quello che visto che siamo in un momento di grandissimo rinnovamento anche proprio delle risorse e delle persone, votare molto sulla formazione e informazione delle nuove leve che entrano in azienda; mi riferisco ai nuovi medici di medicina generale, nuovi specialisti, quindi potrebbe, ma magari lo stanno già facendo, lo hanno già previsto insomma, riprendere in mano il discorso formativo, perché abbiamo un turnover molto, molto alto di personale e quindi per evitare appunto che il nuovo che entra non abbia la stessa sensibilità di chi l’ha preceduto. Però dico, secondo me c’hanno già pensato eh. Che non è che rappresentiamo l’America; dopo di che aumenta sempre di più l’età media, i problemi si pongono in modo sempre più irruento, le persone, i cittadini proprio sono in una fase di stress importante, questo lo vedete, di disagio; insomma, il post pandemia, la pandemia ha slatentizzato evidentemente un disagio che covava sotto la cenere, non posso pensare che sia stato solo un lockdown a far scoppiare tutto sto casino; la gente è incazzata, la gente non sopporta più niente, compreso gli insuccessi in medicina, che invece purtroppo la medicina non è una scienza esatta e quindi probabilmente anche contribuire così a far fronte, a fronteggiare con una corretta comunicazione, divulgazione, formazione i nostri cittadini sul fatto che esiste la morte, esiste, è l’unica certezza della vita, c’è poco da star lì a dirlo ecco, perché oggi sembra che non si possa morire più. Quindi insomma è un discorso che nei momenti di maggior tensione, secondo me riposizionare un po' il valore della medicina, il valore della vita, il fine vita, forse ci potrebbe aiutare; però secondo me lo sanno meglio di me, i bioeticisti.
D:	No, è un ottimo suggerimento, anche pensando a quale può essere un mandato di un servizio comunque istituzionale di questo tipo, questo è sicuramente un ottimo… comunque anche per noi.
R:	Sì insomma, sicuramente è una nostra esigenza, in che maniera ci possano dare una mano è da pensare, ma sicuramente ce la possono dare.
D:	Ho capito. Va bene, dunque come vi dicevo, i punti un po’ che volevo esplorare erano sostanzialmente questi ecco. Le chiedo se in chiusura di questa intervista magari ci sono delle altre considerazioni che ha piacere di condividere, cioè di raccontare rispetto a un po’…
R:	Ma no, direi di no insomma; le cose che… io non mi sono preparata per questa intervista, quindi è proprio molto nature quello che arriva dall’esperienza. Insomma è chiaro che in chiusura insomma non posso che rinnovare questa soddisfazione che noi abbiamo, per essere stati in grado grazie ai professionisti, di sviluppare questa funzione. È chiaro che il marito è tutto loro, noi abbiamo solo il merito di essere stati d'accordo, questo è insomma…. Ci lascerei con un, come dire, con la consapevolezza che quello che esce dai professionisti in genere è quello che chi vuole ecco insomma, il valore delle idee dei professionisti. Questa è la cosa che in questo ambito mi colpisce di più ed oltretutto è proprio un valore scientifico, fa stare bene questa volontà di essere al servizio dei cittadini, in questo caso anche proprio dei cittadini più fragili, che dovrebbe essere la nostra missione. Alle volte non è sempre così, non in tutti gli ambiti è la stessa cosa, questo sì; quindi insomma questa cosa diciamo è una di quelle che ci fa essere contenti di fare questo mestiere.
D:	Ok, sì, sono molto d’accordo con le sue parole, anzi mi sembra un ulteriore mandato diciamo così per questo… per il proseguimento e per gli sviluppi futuri di questo servizio, quindi questo sicuramente, questo sì.
R:	Oltre al fatto che siamo anche tra i pochi a fare ricerca qualitativa, quindi anche per il nostro IRCCS è un tratto distintivo, di cui abbiamo anche molto bisogno, perché di IRCCS ci sono ancora oggi ce ne sono tanti, di IRCCS oncologici che si occupano di ricerca qualitativa e di bioetica non ce ne sono così tanti, anzi.
D:	Molto interessante anche questo aspetto sull'IRCCS, sul valore della ricerca; poi io adesso appunto sto facendo ricerca, quindi quello è il mio ambito e quindi sì, mi rendo conto che dall'esterno, cioè anche confrontandomi con esperienze esterne, su questi aspetti su questi Comitati per l'Etica nella Clinica il valore della ricerca, questo è il mio pensiero, si vede un po’ poco ecco.
R:	Non c’è niente, se non delle suggestioni insomma. Quindi contribuire, creare delle evidenze scientifiche insomma non è poco. Dopodiché è chiaro che oggi se scopri il neurone X probabilmente hai un peso in comunità scientifica diverso, ma dobbiamo continuare a lavorare proprio affinché anche il peso della comunità scientifica della bioetica sia un peso importante, perché i cittadini ne hanno bisogno, tanto quanto di saper cosa (? 26:18), per dire sono una cosa vecchia, ma vuoi interleuchina o immuno qualcosa, insomma, siamo un po’ troppo abituati a vedere la ricerca come qualcosa di biologico, non di etico insomma. Quindi questo è un tipo di ricerca che ha bisogno di essere implementata e sviluppata, insomma noi sia nei pochi che lo fanno. Quindi insomma anche questo è secondo me qualcosa che ci fa distinguere dagli altri.
D:	Ho capito. Bene, allora, mamma mia, mi scusi ma ho un po' la voce un po’ bassa. Io la ringrazio nuovamente tantissimo, anche per il tempo.
R:	Di niente, ci mancherebbe, mi dispiace che abbiamo trafficato tanto.
D:	Beh mah cioè per me veramente problemi non ce sono stati, ci mancherebbe, quindi grazie ancora della disponibilità e della…
R:	Allora buon lavoro.
D:	Anche a lei.
R:	E poi fatemi sapere come è andata a finire questa storia, va bene?
D:	Sì, la terremo aggiornata, grazie.
R:	Arrivederci, di nuovo, grazie.
D:	Arrivederci, buona serata, arrivederci.
Fine trascrizione 00:27:31
```
