# Supplementary material for: How to implement a clinical ethics committee in an oncological research hospital: Qualitative results from a process evaluation study using normalization process theory (EVACEC)
Source: PLoS One. 2025 May 6;20(5):e0318870. doi: 10.1371/journal.pone.0318870 (PMC12054913; doi:10.1371/journal.pone.0318870)
Supplement: S5 File — Interviews transcripts_raw data: this folder contains all the raw data used in this study, the interview transcripts (in original Italian). (ZIP) [file pone.0318870.s005.zip › S5_Interviews transcripts_raw data/03CM.doc]

```
D: 	Allora, ti faccio la prima domanda e ti chiedo se vuoi raccontarmi un po’ la tua esperienza in etica o comunque in ambito bioetico.
R:	Sì, come dicevo l’altra volta, diciamo la mia formazione è prevalentemente di tipo clinico. Già all’Università fondamentalmente, di etica e di bioetica fondamentalmente non se ne parla nella maniera più assoluta. Ovviamente facendo il medico ti trovi di fronte a grosse problematiche di tipo bioetico, per cui alla fine quella che è la mia esperienza in termini di bioetica, fondamentalmente me lo sono maturato sul campo. Facendo il rianimatore poi forse è la parte dove questa componente bioetica, all’interno della professione medica, secondo me raggiunge i livelli più elevati ecco. Penso che un dermatologo o un’oculista insomma, con rispetto parlando, ovviamente ecco, abbiano magari delle considerazioni biotiche molto inferiori a quella che poi ha un rianimatore ecco, penso questo.
D:	Okay. E rispetto a questo, mi vuoi raccontare un po’ qual è stata la tua esperienza con il Comitato per l’Etica nella Clinica?
R:	Allora, la mia esperienza nel Comitato per l’Etica Clinica, penso di farne parte, correggimi magari Marta tu, se sbaglio, da circa due anni; secondo me questo è il tempo in cui questo Comitato è stato creato. Diciamo, la mia esperienza all’interno del Comitato, mi sento un attimo un po’ una figura di secondo piano ecco, nel senso che, come ti dicevo l’altra volta, ci sono dei colleghi – (redacted) – dove secondo me il problema etico lo affrontano da decenni, in maniera secondo me anche molto approfondita ecco. Io senz’altro rispetto a loro sono in una posizione diciamo più di secondo piano ecco. Quello che si discute all’interno di questo Comitato però alcune riflessioni posso portarle all’interno del mio lavoro quotidiano ecco. Poi c’è diciamo un aspetto più veramente organizzativo, che è il seguente: ovviamente per diciamo amalgamare un gruppo in queste riunioni anche online devi scegliere un attimo quello che è l’orario ovviamente più comodo al gruppo e penso che oggettivamente le due e mezza, per il gruppo sono un orario comodo ecco. Io come responsabile di struttura semplice di rianimazione, purtroppo il mio lavoro è una sorta di quiete fin verso le undici, mezzogiorno, da mezzogiorno fino verso le tre e mezza, quattro, diciamo la parte più pesante del mio lavoro, in quanto in questa fascia d’orario fondamentalmente arrivano malati dalle sale operatorie e poi io sono deputato alla comunicazione con i parenti e l’orario dell’inizio di questa comunicazione è attorno all’una e mezza. A volte la comunicazione coi parenti, in giornate particolarmente pesanti, inizia all’una e mezza fino alle quattro meno un quarto ecco, quindi a volte riesco a ritagliarmi un pochino di tempo, dico ai ragazzi “se alle due e mezza qualche d’uno mi può venire a dare il cambio”, a volte soprattutto se è seguito dei casi abbastanza delicati chiamiamo il cambio, secondo me non rende la performance del mio lavoro di comunicazione, secondo me veramente molto scadenti, quindi a volte il cambio volendo i miei colleghi me lo danno anche, non c’è problema, però non mi sento a volte io di chiederlo ecco.
D:	Chiaro, chiaro.
R:	La comunicazione, la cura diciamo del paziente, ma anche della famiglia insomma, non so se ti ricordi, c’era un monito anni fa, I Care, vuol dire io mi prendo cura, che non vuol dire che curo il paziente; certo che curo il paziente, ovvio, ma mi prendo cura di tutto ciò che sta attorno, secondo me è parte integrante del mio lavoro ecco. Quindi a volte purtroppo non sono mai puntuale né mai lo sarò ecco, hai capito, perché ovviamente non posso modificare gli orari d’ingresso di una rianimazione, che sono questi da decenni ecco, tutto qua.
D:	No, poi queste interviste anzi ci servono anche per insomma aggiustare il tiro rispetto anche a questi aspetti organizzativi, quindi va bene.
R:	No, a volte (redacted), come ti dicevo, alle volte alcune riunioni non ci sono stato né ho mandato il preavviso, perché magari se tu me l’avessi chiesto fino a venti minuti, mezz’ora prima avrei detto sì, oggi ho una riunione del CEC, poi sei travolto dagli eventi, capito? E anche mandare un’email, a volte quel mezzo non è opportuno prenderlo ecco, hai capito? Poi ovviamente sembro un attimo un asociale, cose di questo genere, ma vi assicuro che io sono ben altra persona, ecco.
D:	Anzi, ci mancherebbe. Va bene, allora, la prossima domanda che ti faccio invece, se mi vuoi raccontare quali sono le motivazioni che ti hanno spinto a prendere parte a questo servizio.
R:	Una di queste motivazioni l’ho già accennata, che fondamentalmente faccio un lavoro dove la componente bioetica secondo me è abbastanza pesante e non ho alcuna formazione specifica, se non una formazione sul campo, che è derivata dal fatto che ti piaccia o non ti piaccia ti ci trovi dentro ecco, a queste cose. Quindi secondo me è un modo anche per ampliare il mio orizzonte di vedute ecco, perché ovviamente parlando con i rianimatori a volte ti sembra quasi di parlare con te stesso ecco, qualsiasi cosa dici; fondamentalmente tutto il nostro gruppo ha maturato un’esperienza sul campo e fondamentalmente l’esperienza è la stessa, quindi non hai dei feedback perché ti sembra di parlare con te stesso; alla fine di tutti dicono va bene. È come se un mio collega mi chiedesse delle considerazioni la penserebbe alla stessa maniera. Qua fondamentalmente ci sono dei componenti che riescono un attimo a vedere il problema da punti di vista differenti e secondo me è utile anche per la mia maturazione di medico rianimatore, sapere che c’è anche una vision diversa da quella che potrebbe essere un rianimatore ecco. Poi diciamo in questi anni fondamentalmente su certi temi dello stesso file, tipo eutanasia, suicidio medicalmente assistito, cure palliative, sedazione palliativa profonda, secondo me c’è stata una full immersion anche dal punto di vista mediatico ecco. Forse una quindicina, una ventina di anni fa diciamo di questi argomenti se ne parlava o per nulla o molto meno ecco. Ultimamente insomma, dal caso del (redacted) in poi, secondo me è stato via via un crescendo ecco; forse a mia memoria prima nel caso di (redacted) era un silenzio piatto ecco, almeno dal punto di vista mediatico. Di lì in poi secondo me è stato un progressivo crescendo, anche per un semplice motivo: che la medicina è evoluta, siamo arrivati di fronte a delle situazioni dove, forse fine anni Settanta, fine anni Ottanta, non saremmo arrivati. Stiamo portando la vita a limiti estremi, grazie anche a innovazioni tecnologiche, nuove tecniche chirurgiche e diagnostiche e cose di questo genere, che hanno portato a spingersi molto ai confini della vita; ai confini della vita, secondo me problematiche tipo quello dell’eutanasia e mettici tutti i derivati di questo sono diventati secondo me via via argomenti più importanti.
D:	Chiarissimo. Quindi diciamo una motivazione, provo a riassumere per vedere se ho capito, una motivazione di tipo personale, per avere una visione altra su comunque delle tematiche sempre più sentite e verso le quali ci si confronta, cioè ci si è costretti a confrontarsi sempre di più, per i motivi che mi hai chiarificato in maniera veramente molto precisa. Okay, queste erano le motivazioni personali, e invece qual è secondo te il ruolo di questo servizio all’interno dell’azienda? Un po’ mi hai detto qual è il ruolo per te che ha questo servizio, ma dal tuo punto di vista qual è il ruolo che questo servizio ricopre appunto per l’ospedale, per l’azienda?
R:	Penso che come il sottoscritto, tutti gli altri medici, o forse gran parte degli altri medici ecco, non posso ovviamente parlare per altri, una grossa formazione in campo bioetico non ce l’abbiano avuta e quindi hanno un’esperienza di altre scuole di specialità, ma faccio fatica a pensare che in anestesia e rianimazione di bioetica non se ne parli, ma se ne parli, che ne so, in una specialità di medicina interna, cose di questo genere. Quindi penso di poter parlare per me ma penso di poter parlare anche per altri. Secondo me copre un vuoto, che almeno culturalmente penso ogni medico abbia ecco; ad esempio mi sono piaciute le discussioni, ma faccio l’esempio dell’ultima, del dottor Mattia Soliani; tra parentesi è un collega con cui ho avuto modo di lavorare, mi sembra una persona molto pratica e ben inquadrata ecco, che alla fine chiedeva conforto su come si sarebbe dovuto comportare. Mi sembra pratica da questo punto di vista; la discussione, secondo me, sul caso clinico è la cosa fondamentalmente più importante, oltre che ovviamente la discussione su dei temi più slegati dalla clinica e io rinnovo il fatto che quello lì, il file eutanasia, secondo me, è un file abbastanza pesante da questo punto di vista.
D:	E secondo te c’è una specificità proprio di questo servizio, rispetto magari ad altri servizi che vengono proposti dall’azienda? Cioè c’è qualcosa che lo rende specifico?
R:	Sì, […] qualcosa che lo rende specifico secondo me specifico e unico è appunto inserirsi in una sorta di buco formativo, culturale di molti di noi; ognuno fondamentalmente s’è fatto diciamo la propria ricetta personale interna di bioetica, non sempre la propria ricetta personale è la ricetta migliore, però senz’altro ti ha aiutato a sopravvivere.
D:	Ho capito, quindi un’ottica di anche, come si può dire, dare una base comune, una sorta di base comune, avere qualcosa che dia una base comune?
R:	Non tanto una base comune, quanto secondo me il coprire buchi che immancabilmente si vengono a creare; si vengono a creare perché la medicina è in evoluzione, hai capito? Forse negli anni Sessanta o Settanta non c’era questa esigenza, non lo so, io nel Sessanta non ero nato, il Settanta sì, ecco. E secondo me alla fine colma un vuoto, penso questo.
D:	E pensando all’esperienza vissuta in questi due anni diciamo, in che modo secondo te, secondo l’esperienza che hai vissuto con questo servizio, questo servizio è riuscito a colmare un vuoto? Cioè magari se vuoi farmi anche qualche esempio, qualche cosa che ti ha colpito da questo punto di vista, ovviamente nel contesto locale.
R:	Nella mia pratica clinica diciamo è più un modo di vedere le cose; casi clinici singoli secondo me non è molto probabile che arriveremo a trattare, nel senso che la rianimazione è una macchina che si muove alla velocità di una Ferrari. Io devo arrivare a delle considerazioni nell’arco di un giorno, forse due giorni può essere già tardi. Quindi da questo punto di vista nella pratica clinica non so quanto mi possa essere utile, però mi può essere utile sentire e affrontare problemi analoghi da diverse opinioni, da persone che senz’altro fanno un lavoro ben diverso dal mio ecco; cito tra queste persone la (redacted) che insomma è diciamo l’antitesi, fondamentalmente è (redacted), però è importante sapere anche come vede il problema un politico. Poi ovviamente all’interno del gruppo ci sono anche dei clinici, (redacted), faccio un esempio, (redacted)e cose di questo genere, ma con loro sono più sulla stessa lunghezza d’onda. E invece è ben diversa la lunghezza d’onda degli altri componenti.
D:	Ho capito. Bene, la prossima domanda invece che ti volevo fare è… ci sono un po’ anche di domande ripetitive in questa intervista, perché stiamo, cioè segue un framework specifico, quindi l’altra domanda che ti volevo chiedere magari se vuoi approfondire rispetto a quello che mi ha già raccontato è che cosa ne pensi un po’ del valore di un servizio come il CEC per il tuo lavoro e per quello anche dei tuoi colleghi.
R:	Come ti dicevo sul mio lavoro, secondo me più che l’utilità nel caso clinico specifico, ti faccio un esempio ecco, l’ultimo caso quello del dottor (redacted) penso che la risposta al dottor (redacted) sia arrivata nell’arco di – vado a memoria, correggimi se sto sbagliando, (redacted) – un mese e mezzo, tra un mese e un mese e mezzo?
D:	Diverse settimane sì, anche perché poi c’è stata la richiesta, l’audizione a posteriori, sì.
R:	Sì, ecco, comunque non importa, diverse settimane, okay. Da questo punto di vista, nella mia realtà, il “diverse settimane” non esiste, perché io mi muovo alla velocità del suono ecco, hai capito? Quindi a questo punto di vista fondamentalmente, almeno al momento non ci vedo utilità clinica, solo però i casi particolari, dove magari l’utilità del CEC potrebbe verificarsi. Un esempio, a volte trattiamo, noi abbiamo come rianimazione una trentina di ricoveri pediatrici e fondamentalmente sono dei bambini problematici, dove a volte forse ci si è spinti dal punto di vista medico un pochino oltre il ragionevole ecco; quindi a volte ti trovi anche a scontrarti con delle visioni anche di colleghi, ma soprattutto della famiglia che ha una propria impostazione e fondamentalmente ti chiedi quanto può essere etico, essere accondiscendente con questa famiglia, quanto può essere etico prolungare il livello di invasività di cura. E lì forse è l’unico caso dove l’andare alla velocità del suono, che è tipico della rianimazione, in questi casi rallenta molto di più, perché sono figure estremamente medicalizzate, dove se si verifica una problematica paradossalmente si verifica con dei tempi molto, molto lunghi. Quindi all’interno di una parte clinica penso che normalmente no, ma in alcuni casi selezionati, e mi viene in mente soprattutto questi casi pediatrici, con questa vita portata oserei dire oltre l’estremo ecco, secondo me a volte può essere utile fermarsi un attimo e fare alcune considerazioni etiche, soprattutto perché non sempre c’è l’appoggio totale di alcuni colleghi; non tanto sai di questo ospedale quanto gli altri ospedali, che fondamentalmente remano non un po’ diciamo a loro modo, come possa essere anche acceso il nostro ospedale per loro remi in maniera diversa. Alla fine dipende di quale osservatore dà il giudizio ecco; io che osservo loro o loro che osservano me, punto di domanda. La verità non lo so ecco, e qua secondo me potrebbe riuscire essere utile anche in alcuni casi complessi discuterli all’interno del CEC.
D:	Grazie di questa della… sì, anche della prospettiva applicativa di questo servizio.
R:	Sì, io mi scuso delle mie cose, ma è quasi trentuno anni che faccio questo lavoro, io fondamentalmente sono un pragmatico per definizione.
D:	No, certo.
R:	Io vivo nel pragma ecco. Non conviene, conviene, si fa, non si fa, alla fine le mie scelte nell’arco di una giornata sono spesso queste ecco; fino a che punto vale la pena spingersi o non è il caso di tirare i remi in barca, queste sono quelle domande che fondamentalmente a causa del mio lavoro mi faccio almeno più di una volta nell’arco della giornata.
D:	Capisco, deve essere veramente tosta, essere sempre a confronto con queste questioni.
R:	Sì, perché alla fine penso questo ecco, il non decidere non è etico - giusto che parliamo di etica - perché fondamentalmente è il tuo lavoro, se hai scelto di fare il medico hai scelto di fare un lavoro da decisionista, quindi il non decidere non facevi il medico, facevi l’impiegato delle poste o quello che volevi ecco. A volte ti tocca chiederti più volte nell’arco di una giornata: vale la pena fare questo o vale la pena fermarsi? A volte, anzi spesso, non sempre il fare vuol dire fare il bene; a volte il non fare è fare il bene, hai capito? Queste domande, all’interno di una rianimazione, vivendo nella clinica, sono domande che senz’altro ti fai più volte al giorno, senz’altro sono a volte anche dei confronti di opinioni, perché il tuo fare o non fare può essere o non essere condiviso dai colleghi. Noi siamo un po’ diciamo una medicina di squadra ecco, per parlare in termini calcistici, nessun rianimatore è una punta del calcio, fondamentalmente la nostra vittoria è portare avanti la vittoria della squadra, senza che nessuno ci segni, hai capito? Quindi a volte è utile anche fare una situazione di amalgama per quello che è la visione del gruppo, cercando di mettere diciamo d’accordo le varie posizioni ecco, cercare di creare a volte la soluzione su confronti di tipo etico e in cui non c’è concordanza di parere. A volte il mio lavoro è anche quello, arrivare a un dunque, rispettando ovviamente il parere di tutti e senza violentare quella che è diciamo l’opinione di ciascun individuo ecco, a volte proprio il tempo aiuta a maturare.
D:	Okay, ieri so che non era d’accordo su questa cosa, alla luce di queste considerazioni oggi sei della stessa idea? Non sei della stessa idea?
R:	Noi diciamo nel nostro lavoro, soprattutto quando ci sono delle scelte di astensione terapeutica, chiediamo la condivisione non della maggioranza del gruppo ma di tutto il gruppo; un individuo non è d’accordo, si va avanti fino a che quell’individuo avrà maturato una posizione e questa posizione dovrà essere una posizione condivisa tutti gli elementi del gruppo. Giusto per farti un esempio, non so se forse non ho accennato il titolo, quando a volte si decide di fare una riduzione del livello di invasività, che a volte porta alla morte del paziente ci può essere un collega che può non essere d’accordo e in quanto tale deve essere rispettato ecco. Si decide di fare una riduzione dell’invasività del paziente quando tutti gli elementi dell’equipe sono d’accordo su questo; è una sorta di garanzia nei confronti del paziente, è una sorta di garanzia anche nei confronti dell’etica professionale dell’individuo, ecco. Perché se io non ero d’accordo avete deciso di fare questo? No, dobbiamo essere tutti d’accordo, se no avanti tutta.
D:	Sì, ho capito, chiaro, chiaro. Va bene, allora le prossime domande sono domande un pochino più organizzative, rispetto alle quali mi hai già chiarito insomma il tuo pensiero, a proposito un po’ dei limiti organizzativi che mi hai sottolineato, un po’ questo fattore dell’urgenza, cioè del prendere una decisione in urgenza che diventa un po’ complicato, per quella che può essere l’efficacia di un parere del CEC, da quanto ho capito, da quello che mi ha detto.
R:	Sì.
D:	Ti chiedo se…
R:	Salvo quell’eccezione che ti avevo detto.
D:	Sì, nel caso pediatrico.
R:	Nei casi pediatrici iper medicalizzati, dove tutto si muove con una lentezza esasperante, quindi secondo me c’è la possibilità di intervenire, in altri casi secondo me cronologicamente le due cose non combaciano.
D:	Okay. Ti chiedo se invece ci sono degli altri limiti che tu hai percepito, magari di tipo magari anche esterni al servizio stesso, oppure interni, in generale.
R:	Non ho capito la domanda, (redacted).
D:	Se ci sono degli altri limiti che tu hai percepito rispetto a quella che è la tua esperienza con questo servizio, magari anche dei limiti esterni al servizio stesso.
R:	No, perché alla fine diciamo sono due realtà che si muovono con due dinamiche diverse e non sempre riesci a fare collimare diciamo le dinamiche dell’uno e le dinamiche dell’altro; ecco questo non vuol dire che l’uno o l’altro debbano cambiare ecco. Cioè, secondo me alcune questioni devono essere portate col tempo, non serve fretta perché secondo me la fretta è una cattiva consigliera e quindi non vedo elementi migliorativi ecco, semplicemente a volte le due cose non hanno contatto e penso non ci sia nulla da fare a riguardo, anche con un’ottica di tipo amministrativo, perché se il CEC ha dei suoi tempi perché alla fine i tempi sono necessari per sentire l’opinione di ognuno dei membri, come anche la replica di alcuni membri a osservazione dell’altro; capisci anche a te che non possono essere ovviamente in tempo reale, c’è bisogno secondo me di un concerto lasso temporale, che a volte purtroppo non hai.
D:	Sì certo, perché ci sono…
R:	Questo non vuol dire migliorare uno con l’altro, purtroppo… penso che le cose vadano bene così ecco.
D:	Ho capito. Infatti, la seconda domanda che ti volevo chiedere, era se secondo te ci sono invece delle potenzialità di questo servizio o comunque degli elementi anche migliorativi su cui poter puntare, in particolar modo per te che hai anche questo piede nella clinica, molto forte rispetto ad altri componenti. Se ci sono secondo te degli elementi che possono essere o che già hai notato, che comunque possono essere migliorativi per rendere il CEC più (? 29:20).
R:	Secondo me alcuni elementi sono già stati messi in atto, fondamentalmente la partecipazione di clinici all’interno del Comitato Etico secondo me è fondamentale, se no correva un pochino il rischio di essere una bella isola ma scollegata dall’arcipelago ecco, hai capito? Quindi secondo me da questo punto di vista, almeno come costruzione dei componenti, è nato bene; altri elementi migliorativi mi viene da dire fondamentalmente una maggiore visibilità, ma a volte la visibilità si guadagna col tempo ecco, non è che facendo la pubblicità su siti aziendali o cose di questo genere… a volte proprio è il passaparola che aumenta la visibilità del sistema, non tanto mettere sull’internet aziendale questo è il sintomo del Comitato Etico composto da, che si occupa di cose di questo genere. Ecco, a volte diciamo la visibilità si guadagna sul campo e penso che per un Comitato Etico neonato, mi è venuto in mente questo aggettivo…
D:	Sì, è giovane, giovanissimo.
R:	…penso che il tempo sia un elemento di forza, sia un elemento favorevole, devi passare del tempo ovviamente.
D:	Okay. Ecco l’ultima domanda che ti faccio è come secondo te, cioè qual è la percezione che c’è secondo te di questo CEC, che si rifà probabilmente un po’ a quello che mi hai appena detto. Cioè secondo te è stato… cioè come è stato integrato, se c’è stata un’integrazione di questo servizio all’interno dell’azienda?
R:	Io penso che l’integrazione in parte debba ancora avvenire, nel senso che è appena nato e probabilmente le basi di questa integrazione ci sono già; penso che manchi un documento fondamentale che è il tempo, il fattore tempo.
D:	Ho capito.
R:	Penso che non ci sia altro modo per incrementare secondo me la potenzialità di questo Comitato Etico ecco. Ė stato disegnato, bene, è nato, bene, adesso deve cominciare a gattonare e poi camminare su due gambe; è una sorta di piccolo bambino che deve ancora crescere. Almeno, nella mia esperienza personale ho visto le cose vanno così ecco. È l’utilizzo che determina la crescita di un servizio, fondamentalmente credo a questo; poco utilizzato, cresce poco, molto utilizzato vedrai che col tempo terremo giù le basi per sempre un maggior utilizzo ecco. Mi ricordo all’interno dell’azienda ci sono stati dei servizi che prima non esistevano e pianino pianino hanno cominciato a crescere; il crescere è arrivato dal fatto che utilizza una volta, utilizza due volte, poi alla fine c’è una sorta di passaparola, che non è senz’altro la pubblicità sul sito aziendale, perché dopo un po’ le cose non le guardi più. E questo secondo me sarà la vera sfida del futuro di questo Comitato Etico. Cioè non ci vedo altra possibilità, poi magari senz’altro questa possibilità, tu fai questo genere di interviste anche ad altri componenti, magari altri componenti hanno delle idee diverse dalla mia. Io però sono pragmatico ma anche realista, ecco.
D:	Infatti. No, si possono avere tante belle idee, però poi appunto bisogna sempre confrontarsi con la realtà, con le risorse che si hanno, con quello che si può fare e guardare in faccia anche dove… cioè nel senso la realtà in cui è questo servizio e adattare… cioè c’è un duplice, le cose vanno su un binario che si incrocia diciamo. Cioè non si può andare avanti per binari separati, quindi.
R:	Penso questo.
D:	Bene, allora io le domande insomma che volevo farti per questa intervista le ho finite; ti chiedo se magari…
R:	Spero di essere stato abbastanza sintetico; perché i miei colleghi mi dicono che sono logorroico, una volta che comincio a parlare… […]
R:	No, no, assolutamente; invece anche io sono una chiacchierina, ma poi credo che appunto ci sia, cioè ogni parola poi porti con sé dei significati diversi, quindi per un’intervista insomma che ha una finalità con questo progetto anche insomma, che è quella appunto di capire un po’ com’è andato questo servizio, quali cose poter migliorare, quali cose stanno andando già bene, come rinforzarlo, capire quale strada prendere, no, no, invece più esperienze si portano, più pensieri si portano e per me meglio è, sicuramente, quindi assolutamente.
R:	Se dovessi proprio dare un consiglio butterei dentro un altro clinico.
D:	Okay, ne prendo nota.
R:	Faccio un esempio, come clinico, con cui molte volte ci confrontiamo su questo tipo di problematiche è lo specialista in neurologia, dove secondo me è il lavoro che come scelta etica è più simile al mio. Poi ovviamente loro l’affrontano in una maniera diversa, perché c’è sempre un altro dopo che ti risolve i problemi e l’altro dopo siamo noi, ecco. Però questa è una battuta chiaramente.
D:	Sì, sì, ho capito. No, comunque tengo nota del consiglio.
R:	Diciamo però è un lavoro che dal punto di vista etico, secondo me, è abbastanza simile al mio, ecco. Poi ripeto, quello che è detto alla fine è  una battuta.
D:	Sì, ho capito, ci mancherebbe. No, prendo nota insomma del consiglio e poi appunto al termine di questo progetto faremo, ti faranno le valutazioni, verranno condivise comunque con il CEC e appunto è un bambino, sta crescendo e vediamo quando diventerà adulta insomma, per il momento un po’ sta gattonando forse, speriamo.
R:	Per il momento sì, un po’ sta gattonando.
D:	Sì, dai, un po’ sta gattonando. Va bene, allora io ti ringrazio ancora tantissimo per la disponibilità,
Fine trascrizione 00:37:58
```
