# Supplementary material for: How to implement a clinical ethics committee in an oncological research hospital: Qualitative results from a process evaluation study using normalization process theory (EVACEC)
Source: PLoS One. 2025 May 6;20(5):e0318870. doi: 10.1371/journal.pone.0318870 (PMC12054913; doi:10.1371/journal.pone.0318870)
Supplement: S5 File — Interviews transcripts_raw data: this folder contains all the raw data used in this study, the interview transcripts (in original Italian). (ZIP) [file pone.0318870.s005.zip › S5_Interviews transcripts_raw data/03LD.doc]

```
D: 	Ti chiedo, dunque questo è uno studio che stiamo facendo per capire come è stato questo servizio del Comitato per l’Etica nella Clinica, più che altro per capire quali sono state le tappe, qual è stato il processo, anche un po’ per raccogliere dei dati utili per poterlo poi migliorare e replicare in altre realtà, perché fondamentalmente manca un po’ quello. Nella letteratura internazionale bisogna capire effettivamente come questi servizi possono essere implementati all’interno di un’azienda sanitaria e in che modo, per capire un po’ la loro efficacia. Quindi ti chiedo se lo studio ti è chiaro, anche il motivo di questa intervista, se hai qualche altro dubbio a proposito di questo progetto?
R:	Allora, il progetto di implementazione del Comitato per l’Etica nella Clinica in azienda mi è chiaro, nel senso che conosco, ero presente quando è partita la loro progettualità e l’idea di affrontare diciamo questa bella sfida, perché ovviamente non era una cosa assolutamente scontata. Lo studio specifico in cui si inserisce l’intervista non lo conosco, immagino che sia una qualitativa, per valutare quindi… esatto (?)
D:	È una qualitativa e una quantitativa; noi abbiamo raccolto dei dati quantitativi sull’attività che è stata svolta in questi due anni. Abbiamo in progetto anche di fare una survey proprio con i professionisti, per raccogliere per capire qual è un po’ la percezione che c’è da chi, per le persone a cui hai rivolto questo servizio, di questo servizio e poi ovviamente c’è una parte qualitativa in cui abbiamo già intervistato i componenti del CEC, abbiamo intervistato le persone, i professionisti che si sono rivolti a questo servizio e poi la parte che ci interessava e che era secondo assolutamente indispensabile però per approfondire appunto con delle interviste qualitative il punto di vista di chi ha promosso in prima persona questo servizio, quindi…
R:	Okay, grazie, no, mi è chiaro, perché avevo diciamo l’idea del progetto complessivo, diciamo del progetto ombrello, ma nello specifico in questa fase cosa… okay. No, allora io ti posso raccontare quella che è la mia…
D:	Sì, assolutamente, infatti la prima domanda sarebbe stata proprio questa, qual è stata un po’ la tua esperienza con questo percorso.
R:	Allora, quando è iniziata la progettualità per il Comitato nell’Etica Clinica, così come devo dire la progettualità sullo sportello Scegliere DAT, quindi per entrambi i progetti ero in direzione sanitaria, quindi mi occupavo già di attività, ero collegata diciamo all’attività di ricerca, perché essendo una referente per l’IRCCS ero comunque già coinvolta. Quindi nei progetti diciamo più strategici, quelli più grossi dell’IRCCS venivo comunque coinvolta anche solo nella progettazione, anche se non ero nel gruppo di ricerca, comunque nella valutazione di come poter implementare. Quindi ricordo che sia il vecchio Direttore scientifico, (redacted), che è la responsabile dell’unità di bioetica, (redacted), avevano appunto illustrato quelle che erano le caratteristiche del… e ricordo di aver cercato di facilitare la costituzione. Poi dopo non sono entrata ovviamente nel gruppo di ricerca, nel vivo e Comitato per l’Etica Clinica; conosco la composizione, conosco come è strutturato, ho partecipato anche al corso di formazione che è stato fatto nel 2020, ‘21 non ricordo,
D:	‘21.
R:	Nel ‘21, esatto, nel ‘21 e la percezione che ho da esterna, non avendo, non essendo né componente del CEC né professionista che ha avuto accesso per chiedere un supporto per l’assistenza, è comunque quello che nell’ambito della nostra azienda il comitato abbia funzionato molto bene. Nel senso che a differenza di altri organismi di supporto, di consulenza che ci sono per le aziende che sono strutturati perché previsti dalla norma, dove a volte si sentono i professionisti che si lamentano perché in realtà non hanno ricevuto il supporto che volevano, oppure peggio, hanno avuto degli ostacoli posti su altre cose, sul CEC nel tempo ho raccolto feedback positivi. Quello che diciamo da persona che ha seguito lo sviluppo del progetto e che sa che ora è terminato e che bisogna capire poi come implementarlo successivamente, credo che il tema non sia tanto quello qualitativo, cioè di come funziona e delle competenze che ci sono dentro e di quanto è importante, perché è decisamente importante, poi la norma nel frattempo si è voluta, assegnando tra l’altro ai Comitati per l’Etica Clinica anche delle funzioni chiare. Come la consulenza per il suicidio assistito, che subito sembrava competenza dei Comitati Etici, poi dopo è diventata dei Comitati per l’Etica Clinica o del CEC, se non c’è il Comitato per l’Etica Clinica, e non solo comunque. Quindi sicuramente il fatto che vada mantenuto e che va e che sia reso disponibile non solo nella nostra Provincia, ma per tutte le aziende, ne sono convinta; sicuramente c’è un tema di sostenibilità, perché è ovvio che tutti i… questo vale per tutti i servizi, che quando noi li studiamo per capire se funzionano, quando abbiamo l’evidenza dobbiamo però capire come renderlo sostenibile, come quando dimostriamo che uno screening o farmaco o un intervento sono efficaci, però poi devono entrare nei LEA o nei prontuari, perché se no non lo possiamo offrire. Quindi sicuramente il tema della sostenibilità è importante per la diffusione e credo anche, ma questa è in realtà un’impressione, perché non avendolo vissuto, però immagino che ci sia anche un tema importante di formazione sui professionisti, perché quei professionisti che hanno partecipato al progetto, dandosi disponibili per far parte come componenti del Comitato per l’Etica nella Clinica, sicuramente sono dei professionisti che si sono auto selezionati, nel senso che hanno delle caratteristiche personali e di formazione specifica. Pensare di poter offrire questo servizio su larga scala significa avere immagino più professionisti; non penso che un Comitato ridotto possa servire, non so, un’intera Regione o di più. Quindi sicuramente la formazione è importante, perché vedo che anche per un Comitato, che può avere delle similitudini ma non è assolutamente uguale, com’è il Comitato Etico, all’interno del Comitato Etico loro stessi capiscono e valutano che su certi aspetti non sono formati e c’è la necessità di una formazione continua. Quindi sicuramente l’impressione che ho avuto è che il progetto ha funzionato molto bene, ha gettato le basi per dire che deve essere mantenuto come servizio, quindi entrare nella diciamo istituzionalità e deve essere però sostenibile; per essere sostenibile deve essere finanziato, perché diversamente non so come… non ci si può basare sulla ovviamente gratuità delle persone per sempre e poi bisogna investire anche in termini credo - questo lo sanno sicuramente chi ci ha lavorato dentro, chi è stato nel Comitato - in termini di formazione, perché sono ambiti molto particolari e dove effettivamente ogni professionalità può dare il proprio contributo; sicuramente il bioeticista ci deve essere, però ci devono essere anche altre figure, che devono avere una formazione specifica. So che la Regione ha intenzione di investire nei Comitati per l’Etica Clinica già dalla precedente gestione, ma anche quella attuale; questa mi sembra una buona luce rispetto alla sostenibilità, nel senso che effettivamente il fatto di dire metto una persona dedicata, do un gettone di presenza ai componenti del CEC, lo istituzionalizza, poi il mio punto di vista, soprattutto adesso che sono di qua, perché prima partivo della direzione sanitaria, avevo l’occhio sui pazienti, quindi la ricerca è un’opportunità per il paziente. Ancora adesso per me è il paziente, l’ultimo e quindi il paziente, il soggetto, l’utente, perché se parliamo di altri progetti magari non… fortuna loro non sono ancora pazienti o malati. Il fatto di poter fare ricerca sui servizi è un’ottima cosa, è bellissimo, è sfidante, la cosa difficile è poi farla entrare nel sistema. Anche perché mentre il farmaco lo decide il livello nazionale, cioè noi contribuiamo come Istituto di Ricerca, come tutti gli altri a creare evidenza su quel farmaco, su quel dispositivo, su quell’intervento chirurgico, radioterapico, però poi ci sono degli Enti regolatori che decidono se quello entra o non entra e dopodiché tu lo puoi acquisire. Sui modelli assistenziali è un po’ più difficile, non è così scontato e soprattutto succede che finché puoi finanziarlo, autofinanziarlo lo mandi avanti, poi arrivi un punto ovviamente dove, dove… Ecco, io credo che siamo nella situazione in cui essendoci una sensibilità regionale, siamo nelle condizioni di poterlo esportare, esportare e allargare, cioè trasferire ecco, in generale. Sul livello nazionale non lo so cosa si possa fare, bisognerebbe capire se altre Regioni sono disponibili, sono sensibilizzate, hanno la possibilità di poter investire. Non è un investimento enorme in termini economici, mi vien da dire, nel senso che poi con qualcuno dedicato, un gettone di presenza, cioè non stiamo parlando di un farmaco da 500.000 euro a paziente ecco. Quindi, in termini di trasferibilità credo che come tutti i modelli la cosa più difficile sia sempre la ricollocazione nel contesto; cioè perché questo modello funziona qui perché il nostro contesto è questo, il nostro contesto tra l’altro è molto favorevole a questo tipo di progetti, perché abbiamo dei professionisti abituati a questa tipologia di sperimentazione, di innovazione diciamo, perché a volte magari non è neanche innovazione per la ricerca, innovazione a migliorare e abbiamo una comunità molto stretta, adesa ecco, associazioni di pazienti, cioè una comunità che è attaccata comunque all’ospedale, all’azienda. Ci sono altre realtà, adesso la Regione secondo me è abbastanza simile a noi, magari ci sono città più grandi, come Bologna, che hanno una diversa ovviamente… però posso immaginare che Modena sia simile. Cioè come… però ci sono altre realtà dove questo sentito, penso a Milano, che ha molteplici istituti e ospedali, non vedo la comunità milanese adesa a un punto di riferimento e disponibile a partecipare attivamente. Quindi in questo senso dico che il contesto può ritarare ovviamente il…
D:	L’intervento, sì.
R:	Esatto, quindi sicuramente sulla trasferibilità le difficoltà che vedo sono quelle di tutti i progetti insomma sui modelli, nel senso che li devi ricollocare nel contesto: però la cosa positiva è che se io penso a una trasferibilità a livello regionale è vero che i contesti regionali sono abbastanza simili, non li vedo così… Ecco, la trasferibilità in Alto Adige o in Calabria le vedo più difficili, perché sono contesti secondo me proprio sociali e insomma, dato che qua siamo nell’ambito valoriale, perché alla fine… e quindi il contesto sociale, economico, politico può effettivamente cambiare. Però sul fatto della… cioè che la bontà del progetto sia… insomma sia un risultato ecco, poi insomma lo tirerete fuori voi, coi dati quantitativi e qualitativi; io sto già dicendo che… sto già dicendo per me è il risultato buono, ma ovviamente è il… Però con un occhio sia alla ricontestualizzazione che alla sostenibilità, questo sicuramente. Poi, come esperienza posso dire che sono molto contenta di avere appoggiato all’inizio questo progetto, anche se poi non lo ha seguito, anche perché ho avuto le mie vicissitudini personali, nel senso che prima è arrivata la pandemia, poi ho cambiato lavoro, poi sono ritornata qui, quindi… però insomma da lontano, tant’è che quando ero a Guastalla era uscito il corso, ho detto iscriviamoci al corso sul CEC, anche solo per sapere, insomma per diffondere l’informazione e sapere che cos’è e quali sono le capacità. Tra l’altro, l’altro giorno stavo, ero in macchina con delle ricercatrici, cioè con una ricercatrice e una clinica, che stavamo andando a un corso a Bologna e stavo proprio parlando del CEC e non sapevano cos’era il CEC, gliel’ho spiegato e una mi ha detto “ma noi abbiamo, al PDTA stiamo discutendo il caso…” un caso molto particolare di una signora, dove c’è un aspetto molto complesso dal punto di vista, eccetera; ho detto ecco, questo sarebbe un caso, arriverà anche quello, un caso dove o se non da CEC comunque sicuramente da consulenza bioetica, perché è un caso molto particolare e quindi credo che al di là… ecco, questa cosa mi fa venire in mente che per renderlo di nuovo sostenibile nel tempo e trasferibile, oltre che la formazione su chi fa parte del CEC, deve essere formata a temi specifici, ci vuole una grande opera di informazione e formazione sui potenziali clienti, passami il termine; cioè su tutti quegli operatori che devono rivolgersi al CEC. Perché non è ancora così chiaro, nel senso che… ma lo so, perché non è un limite di chi ha proposto il progetto, è che è normale. Cioè tanti progetti innovativi, tanti servizi bellissimi anche, che diamo ai pazienti, a volte i pazienti non li ricevono, non ci accedono, perché l’operatore non lo conosce; nonostante un battage informativo e quindi insomma…
D:	C’è qualcosa che secondo te da questo punto di vista può funzionare meglio rispetto a quello che è stato fatto? Perché appunto abbiamo fatto, sono stati fatti dei corsi di formazione, comunque sono presenti dei professionisti interni ma anche esterni all’azienda, però interni, quindi secondo te c’è qualche cosa che si potrebbe fare, qualche strategia particolare per…?
R:	Allora, voi avete fatto degli incontri di formazione rivolti diciamo a tutti, giusto?
D:	Sì, sì.
R:	Nel senso, forse la cosa che potrebbe essere utile, forse, è da capire, è passare in quegli incontri che vengono fatti dei dipartimenti periodici e gli incontri di dipartimento; nei Comitati di dipartimento ci sono tutti i Direttori e coordinatori delle strutture che afferiscono a quel dipartimento. Cioè per esempio, il dipartimento oncologico, oltre che a Versari e Guberti ci sono tutti i Direttori dei reparti che afferiscono e i coordinatori. Forse un passaggio lì per ribadire, facendo il giro di tutti i dipartimenti, che lo so che è oneroso, però insomma può portare vantaggi, per ribadire anche portando il risultato, magari nella fase in cui avrete il risultato, portare il risultato e raccontare com’è andata, quali sono le possibilità. Credo che quello potrebbe essere un buon punto. A me vengono in mente anche i gruppi multidisciplinari di patologia, dove ci sono i professionisti che si occupano in particolare di quella patologia, perché per esempio, nel caso che ti facevo prima, era proprio il caso di una signora che era stato discusso nell’ambito di un gruppo di patologia, dove sostanzialmente non sapevano come andare avanti con il trattamento, perché c’erano degli aspetti molto particolari, relativi sia a quello che aveva che alla patologia di base, che è una SLA. Quindi è una paziente con SLA, con patologia oncologica con dei problemi insomma di tipo complesso. Quindi forse anche quelli, è vero che magari la patologia oncologica non è tra quelle… cioè adesso non conosco bene la casistica che sia arrivata alla CEC in questi anni, però non mi aspetto che l’oncologica sia quella preponderante, secondo me sono più le degenerative, oppure la neonatologia, la pediatria.
D:	Sì, diciamo che per esempio con le demenze stiamo lavorando, anche ieri abbiamo avuto un caso insomma.
R:	Esatto, cioè immagino, poi magari arriva qualche oncologico quindi forse quel passaggio lì se c’è tempo, perché prendendo i gruppi di multidisciplinare di patologia che sono dodici, prendi tanti professionisti, però magari l’oggetto è più difficile che sia una patologia che necessita, un caso diciamo che necessita di… Forse valutando anche quelli che hanno fatto più ricorso a voi, vedendo anche in letteratura quali sono i casi che più, magari andando proprio nel reparto, cioè entrando proprio, non so, in rianimazione piuttosto che in pediatria e in neonatologia, dove però immagino che lì ci sia già una sensibilità legata al fatto che voi avete già fatto consulenza e quindi…
D:	Diciamo che sono tra… anche in neonatologia effettivamente sì, l’unità di bioetica ha lavorato parecchio. Ecco, magari rispetto a questo ci sono, mi ha già detto tantissime cose, quindi veramente, però ci sono delle cose che mi hanno un po’, come dire, mi ha fatto pensare rispetto a quello che dicevi prima; un po’ questa questione dei servizi. Hai citato alcuni servizi rispetto a cui questo CEC diciamo è particolare; quindi magari pensando anche all’attività che viene fatta dall’unità di bioetica, ti chiedevo qual è secondo te la specificità diciamo di questo comitato, e anche le motivazioni se magari si afferiscono a questa specificità, per cui secondo te, cioè all’inizio hai pensato che ne valesse la pena ecco.
R:	Certo. Cioè io ti dico quello che mi sono sempre immaginata e ho pensato del CEC, poi magari sbaglierò, perché non… Io credo che allora intanto il CEC ha un valore perché è un Comitato e quindi è una valutazione collegiale, che per definizione prende più punti di vista. Diverso è una consulenza intesa come io sono il consulente, vengo e ti dico questa cosa; e perché è importante? Perché di solito si affrontano situazioni complesse proprio dal punto di vista etico, cioè dal punto di vista etico per me è quando il problema non è la norma, poi può esserci anche la norma, che magari confligge con un aspetto di… ecco, però il problema è proprio sul livello valoriale, almeno questo è quello che avevo un po’ capito all’inizio. Nel senso che il CEC aiuta e come dire, la diade e in alcuni casi triade, operatore-paziente-famiglia, a prendere la scelta, a effettuare la scelta migliore per il paziente, tenendo conto però dei punti di vista della famiglia e dell’operatore. Perché assumendo che non si parte mai dal presupposto che l’operatore vuole nuocere al paziente, ma vuole sempre fare la cosa migliore per il paziente, però a volte ritiene di fare la cosa migliore ma non è la cosa migliore, quindi questo è il punto. Stessa cosa vale per la famiglia, che comunque ha le sue esigenze, le sue necessità, i propri valori. Quindi secondo me, tutte le volte che in un reparto, in un servizio ci si trova davanti a una situazione molto complessa da questo punto di vista, il fatto di avere un parere terzo, rappresentato diciamo nelle situazioni se vogliamo un po’ più semplici dalla consulenza del bioeticista e quelle molto complesse da un intero comitato, secondo me dà una maggiore garanzia di serenità diciamo, rispetto al fatto che quello che si deciderà […] nel senso che ci sono effettivamente delle situazioni in cui cioè serve veramente un supporto per capire qual è la decisione, ripeto, cioè io utilizzerei l’aggettivo il più serena possibile, perché corretta non lo so, serena possibile da tutti i punti di vista, nel senso che a volte in cui, cioè io immagino che siano quelle situazioni, alcune le ho anche viste. Quelle situazioni in cui il paziente ha un punto di vista o peggio non ce l’ha, cioè non lo riesci a sapere perché non è in grado di comunicartelo, un familiare ha un punto di vista, magari un altro familiare ne ha un altro ancora e l’operatore ne ha un altro ancora. E per arrivare alla decisione non è tanto un aspetto legale, poi ci può essere anche quello, ma più un aspetto etico, di cercare di arrivare. E quello che ho visto io è che quello che si cerca di fare nel supporto etico e nel supporto del Comitato è di analizzare e valutare tutti i punti di vista e tutti gli aspetti valoriali e poi dare insomma una consulenza rispetto a quello che potrebbe essere la cosa. Infatti insomma penso alle situazioni appunto in cui i pazienti non possono esprimere il loro consenso, il familiare ha una sua idea, l’operatore dice “no questa cosa va contro il paziente” e quindi c’è… La vedo anche molto come non devo decidere se hai ragione tu o tu o tu, ma devo cercare di arrivare a far sì che tutti pensiamo che questa sia la cosa migliore da fare, ecco; poi non so se vivo nel mondo delle illusioni o che…
D:	No, no, no, ma è proprio per capire.
R:	Ė quello che mi sono sempre raffigurata io. Quindi il CEC è fondamentale perché senza il CEC, l’unica pezza che rimane per questi operatori che si trovano a gestire il paziente e la famiglia è sentire il medico legale e la direzione sanitaria, che però non sono la stessa cosa, che invece confrontarsi con appunto un Comitato di professionisti, dentro i quali ci sono non solo clinici ma anche bioeticisti, poi c’è anche il medico legale. Insomma è un…
D:	Gli esperti delle patologie.
R:	Esperti di organizzazione, perché penso che anche la (redacted) ha l’occhio non solo sulla clinica e l’assistenza ma anche sull’organizzazione. Quindi insomma io credo che sia importantissimo e fondamentale e mi sembra anche non solo un problema, ma un peccato veniale tornare indietro. Cioè togliere questo servizio quando l’hai reso disponibile, anche perché ci può essere una potenziale richiesta; quindi insomma quello che posso dire come, non lo so, stakeholder, che cercherò di farlo portare avanti. Però insomma, su questo so che la Regione ci vuole investire, quindi sono ben felice; di tutte le cose che non sta facendo la Regione, almeno una.
D:	E un’altra cosa che ti volevo chiedere, tornando un attimo indietro, è quali sono stati i passaggi che hanno portato alla costituzione di questo CEC? Se te li ricordi e se rispetto a questi pensi che sia opportuno magari modificare qualcosa. Cioè proprio rispetto a come è stato implementato.
R:	Sto cercando di ricordarmi perché non so se me lo ricordo. Allora sicuramente dall’idea progettuale sicuramente se ne è parlato con la direzione, perché non si poteva fare una cosa del genere senza parlarne con la direzione generale, che si era mostrata ovviamente disponibile e interessata. E mi pare che poi si siano contattati i vari servizi di riferimento, dove poi c’erano i potenziali componenti; non mi ricordo sulla composizione come è avvenuta la chiamata, cioè se c’è stata una chiamata diciamo ad hoc, su persone che si conoscevano, si è stata così.
D:	Sì, infatti, anche perché aveva un aspetto sperimentale, quindi questo è un po’ il (? 28:00) diciamo.
R:	Esatto, sì, non era una call diciamo e penso che forse neanche adesso potrebbe essere così, come la fanno per il Comitato Etico, proprio perché secondo me non ci sono sufficienti professionisti formati, per poter capire a cosa eventualmente decidono di aderire, penso. Nel senso che se… cioè poi su questo valuterete voi ovviamente, perché invece magari in questi anni è stata talmente diffusa è talmente chiara che magari ci sono operatori che hanno ricevuto essi stessi una consulenza che ritengono di poter… e poi dopo ci sono stati tutti i passaggi formali, ovviamente di formalizzazione del Comitato e poi dopo è stato fatto il sito dedicato, cioè lo spazio dedicato e poi sono iniziate le… ci sono le sedute che non mi ricordo se sono periodiche, definite, giusto? E poi dopo si raccoglie, si va alla seduta e si discute quel caso, esatto. C’è una verbalizzatrice che sei tu, credo…
D:	Sì, sì, io sono l’addetta…
R:	Con tua grande gioia, sì, come segreteria del CEC e poi la formazione che dicevi prima, quella ce l’ho presente, anche perché ho partecipato come discente e basta. Io, sì però sugli aspetti iniziali mi sembra che i passaggi fossero stati nascita diciamo in seno alla direzione scientifica, come progetto, passaggio in direzione per… e poi ovviamente passaggio in Comitato Etico per il progetto; cioè il percorso diciamo dei progetti di ricerca, però sull’aspetto più di formalizzazione non me lo ricordo, se ti devo dire la verità. Ho più memoria sul percorso DAT e Scegliere, perché lì era più un discorso anche di intercettazione dei vari Comuni, dei vari soggetti esterni e insomma almeno all’inizio c’era stata anche la survey sulle disposizioni e su quello ero più… Sul CEC ero più diciamo informata che partecipante, insomma che ho preso parte, quindi ricordo questo, di più non ricordo.
D:	Okay, sì, magari se c’era qualcosa che ti era rimasto di questo percorso che dicevi no, quella cosa effettivamente non andava bene così.
R:	No, no, se non devo pensare che c’è qualcosa che non è andato bene io proprio direi di no, nel senso anche col senno di poi, anzi bene, perché ha un po’ precorso i tempi, nel senso… e poi ricordo che anche sulle DAT, quando cominciammo a parlarne era passato un anno dalla legge, l’altro giorno (redacted) mi diceva sono già cinque anni.
D:	Sono già cinque.
R:	Il tempo passa, Time (? 30:47) esatto.
D:	Sì, io ho quasi finito il dottorato, mi pare ieri che però, sì… sto un attimo ricontrollando, visto che hai anche questa riunione, se c’erano delle cose… Una era anche la questione dell’integrazione, delle attività nel contesto, che però insomma da quello che mi ha raccontato un po’ attraverso la formazione, che ha dato un input, però anche attraverso questi incontri anche informativi, però anche mi ha molto colpito quell’esempio che avevi fatto prima, del caso che avevi discusso in maniera un po’ informale; cioè è un’informazione che c’è, però forse non è questa conoscenza, la percezione che è tua che non sia ancora così…
R:	A tappeto, ma ripeto, anche per esperienza mia, avendo seguito altri progetti, organizzativi o di ricerca, ed essendo andata in tutte le stanze, cioè comunque non riesci a prendere tutti, c’è sempre qualcuno che non ce l’ha, hai capito? Perché è una cosa, cioè non è una cosa in cui incappi, non so come dire, cioè è una cosa che ti devi andare a cercare, quindi come tutte le cose che ti devi andare a cercare come opportunità, tu hai per il tuo paziente, ce la devi avere, ce la devi avere lì e per avercela lì vuol dire che o l’hai già utilizzata e allora sai qual è ovviamente la potenzialità, oppure c’è qualcuno che ti…
D:	Che te la ricorda.
R:	Ti faccio un esempio differente, il progetto della Sara Paltrinieri sul supporto al rientro al lavoro dei pazienti oncologici è un’opportunità bellissima per un paziente oncologico, cioè avere uno sportello dedicato, un terapista occupazionale che ti supporta o dal punto di vista informativo o addirittura assistenziale sul rientro al lavoro in serenità. Bene, non so quanti incontri, ancora c’è qualche oncologo ematologo che non sa cos’è, oppure che sa cos’è però dice “già, è vero mi sono dimenticato di dirglielo”. Cioè quindi è proprio una fatica, io penso che…
D:	Come una cosa costitutiva, cioè proprio di questi servizi più che altro.
R:	Sì, hai capito? Cioè, almeno la mia sensazione è quella, che o è un passaggio obbligatorio, ma questo non potrà mai essere un passaggio obbligatorio, cioè il Comitato Etico è un passaggio obbligatorio, se vuoi fare approvare un progetto di ricerca ci devi andare quindi non puoi non sapere che non esiste o non ricordartelo, perché ci devi andare, così come altre cose. Invece un servizio come dire in più, cioè in più, un qualche cosa, ecco forse dovrebbe non essere più un qualcosa in più, cioè dovrebbe entrare nella testa degli operatori che è un qualche cosa che c’è e che va usato quando serve ecco.
D:	Infatti l’obiettivo anche di questo progetto, pensando anche in termini di ricerca, in letteratura, l’outcome principale è quello dell’efficacia. Che spesso viene valutato proprio in termini anche di numero di richieste, termini molto quantitativi. Adesso la letteratura si sta spostando, cioè sta spostando questo outcome dall’efficacia in questi termini quantitativi alla normalizzazione.
R:	Esatto, alla normalizzazione.
D:	Difatti anche la teoria che abbiamo utilizzato per impostare questo progetto è quella della normalization process theory, si chiama, cioè proprio un progetto strutturato per capire come questo servizio è stato normalizzato. E quindi anche rispetto ecco a questo, all’obiettivo di questa ricerca un po’ anche per concludere diciamo, se rispetto a questo magari hai qualche pensiero specifico su come è stato integrato, su come dal tuo punto di vista sono state integrate; prima hai detto che secondo te è un servizio che sta andando bene, se ti va di raccontarmi quali sono secondo te, quali sono i tuoi parametri per dire che qui si sta andando…
R:	Allora, sicuramente il feedback della soddisfazione di chi l’ha utilizzato. Nel senso che comunque il fatto di vedere degli operatori più tranquilli, più sereni rispetto a delle scelte, delle cose fatte dopo la consulenza, secondo me significa che è già un risultato; cioè poi magari non sarà il risultato sul paziente, però io vedo un risultato sull’operatore, perché ovviamente non mi interfaccio con ogni singolo paziente, ma mi interfaccio con gli operatori. È un po’ come… cioè per me bene è quando vedo che c’è maggiore serenità rispetto a una, come si dice, a delle scelte che vengono effettuate. Questo per me è bene e l’altro aspetto è il fatto che comunque le richieste sono arrivate. Adesso state facendo l’analisi del quantitativo però non mi risulta che vi siate trovati per dirvi non ci è arrivata nessuna richiesta, e lo so, raccontiamoci le cose nostre perché non abbiamo… Cioè quindi di richiesta ce n’è stata, quindi vuol dire che la domanda c’era, quando c’è stata la domanda c’è stata, è stata data una risposta e io ovviamente non è che sono andata a valutare tutto quello che è stata la percezione negli operatori, però in alcuni casi mi è arrivato un feedback molto positivo. Fuori è già arrivata anche, ecco un’altra cosa, il fatto che fuori si sappia che questo c’è, che funziona e che stimoli la voglia di esportarlo o comunque di allargarlo, per me è un altro risultato, perché vuol dire che funziona. Cioè quando metti in piedi una cosa che non funziona, non ti arrivano le richieste, hai qualche feedback, o non ce l’hai proprio oppure ce l’hai negativo e da fuori non c’è nessuno che si attiva, fuori Provincia. Quindi questo per me, la mia percezione è che stia andando bene, questo ecco, quindi non lo so, io spero che la ricerca confermi, che ti torni, dato che…
D:	Sì, sì, sì, adesso sto guardando, cioè come domande poi, ti dico, le abbiamo, anzi mi hai risposto a tutto praticamente; questo dell’integrazione, dei limiti anche lì, li abbiamo un po’… me li hai raccontati, le potenzialità direi che è molto chiaro. Guarda, io le aree che volevo esplorare direi che le abbiamo esplorate tutte. Ovviamente l’ultima domanda che ti faccio è se rispetto a tutto quello che mi ha raccontato c’è qualcosa che ci tieni ulteriormente a dirmi, che magari… una roba che hai avuto modo di vedere in questi mesi e che secondo te è importante condividere ecco, ai fini anche della ricerca, della valutazione, del proseguo di questo servizio.
R:	No, cioè tengo a sottolineare un aggettivo, che è sfidante, e come tutte le cose sfidanti è bellissima, cioè è bellissima, almeno per come sono io, le cose sfidanti sono bellissime, perché lanci sempre il cuore diciamo al di là dell’ostacolo, però è una fatica… cioè immagino anche la vostra fatica, proprio perché tutte le volte che lanci qualcosa di innovativo, in termini di ricerca e di… è molto bello, è un bellissimo sogno, secondo me, ma sogno non nel senso che non si realizzerà mai, cioè un bellissimo obiettivo è molto faticoso, però ne vale assolutamente la pena, ecco. Questo mi sento di… cioè, questo è e non a caso prima ti ho detto questo è un progetto secondo me strategico; non per dire te lo dico a te perché ci sei dentro, ce ne sono pochi così strategici, secondo me. E prendimi la parola progetto non solo come progetto di ricerca, progetto innovativo.
D:	Okay, la progettualità.
R:	Esatto. No, mi sento di dire quello, cioè che proprio è un bel progetto, infatti sono contenta di averlo visto nascere, crescere e adesso speriamo crescere ancora di più, ecco. Camminare, esatto; dai, camminare cammina, correre.
D:	Correre, okay.
R:	E così. Va bene?
D:	Va bene, perfetto. Allora, ti ringrazio.
Fine trascrizione 00:39:49
```
