# Supplementary material for: How to implement a clinical ethics committee in an oncological research hospital: Qualitative results from a process evaluation study using normalization process theory (EVACEC)
Source: PLoS One. 2025 May 6;20(5):e0318870. doi: 10.1371/journal.pone.0318870 (PMC12054913; doi:10.1371/journal.pone.0318870)
Supplement: S5 File — Interviews transcripts_raw data: this folder contains all the raw data used in this study, the interview transcripts (in original Italian). (ZIP) [file pone.0318870.s005.zip › S5_Interviews transcripts_raw data/05CM.doc]

D:	Intanto grazie mille davvero per partecipare a questa intervista, che insomma è per valutare un po’ quello che è stato questo servizio nel corso di questi mesi e quindi grazie davvero. Sarà un’intervista un pochino ripetitiva, però questo è dovuto anche un po’ al framework, alla tipologia di teoria di riferimento che stiamo adottando per portare avanti questa valutazione, quindi può essere che ci siano delle domande un po’ ripetitive, però ecco ti chiederò se magari avrai qualcosa da aggiungere, sennò sentiti libero anche di dire “no guarda, ho già esaurito”. Quindi, io direi di partire con la prima domanda, che è una domanda abbastanza generale; ecco, ci saranno delle domande un po’ più personali, su quella che è stata, che è la tua esperienza e su come invece è secondo te questo servizio. La prima domanda che ti faccio è se vuoi raccontarmi un po’ qual è la tua esperienza nell’ambito della bioetica, dell’etica clinica.

R:	La mia esperienza è un po’ legata al mio mestiere, che è quello del medico legale, nel cui insegnamento a medicina c’è anche la materia deontologica, cioè la deontologia professionale, fa parte dell’insegnamento che viene presentato, a cui noi siamo formati e in cui gli aspetti bioetici sono molto spesso intimamente collegati. La materia quindi medico legale ha una sorta di affinità - usiamo il termine di affinità - con quella deontologica e di conseguenza anche bioetica, quando parlo di deontologia parlo di deontologia professionale medica ovviamente. L’affinità fra le due discipline, io tra l’altro per un certo periodo sono anche stato… avevo anche un incarico quando lavoravo a Modena al Policlinico, avevo anche un incarico di insegnamento, facevo cinque ore di bioetica, che era rivolto alle ostetriche, al corso di laurea in ostetricia; erano cinque ore che io dividevo a metà in deontologia e l’altra metà in bioetica. Ovviamente le ostetriche hanno i problemi dell’aborto, della procreazione assistita, di tutte queste problematiche; c’era poco, anzi niente sul fine-vita, che oggi invece è uno dei primi forse più importanti, anche se quello dell’aborto tornerà, viste le recenti… Quindi la risposta è questa, cioè un corso di specializzazione che mi ha introdotto all’argomento e poi c’è anche un mio interesse particolare, oltre che per l’attività professionale. Anche perché io, come servizio, ho sempre dato la disponibilità a svolgere delle consulenze per i colleghi, per le strutture dell’azienda, per la direzione, per dei professionisti. E questa qua è un’attività che spesso ha delle questioni, non sempre, spesso ha anche delle questioni di tipo bioetico e a volte dei veri e propri dilemmi, tant’è che quando parlammo con (*redacted*) io vedevo benissimo l’introduzione di questo Comitato per l’Etica della Clinica, perché avrebbe potuto apportare conoscenze non solo medico-legali. Allora il medico legale per definizione un materialista puro, io ho imparato il mio mestiere facendo delle autopsie, per cui io vedo le cose che sono molto diciamo così materiali; per cui avere anche se diverse, anche non mediche, per me è un notevole vantaggio per definire questo tipo di problematiche, che sono poi quelle che ci vengono proposte più o meno spesso. Questa è la mia risposta.

```
D:	Infatti un’altra domanda che ti volevo fare era un po’ qual è stata, quali sono le motivazioni che ti hanno spinto a prendere parte a questo servizio?
R:	La motivazione proprio dell’interesse sulla materia; l’interesse sulla materia che, ripeto, era un po’ fuori, non è sempre di nostra immediata percezione, ma è un interesse culturale, oltre che pratico, perché poi alla fine noi facciamo tutte delle costruzioni teoriche, ma noi dobbiamo dare una risposta su degli aspetti pratici, cioè come l’altro giorno – quello dell’altro giorno non so come si possa definire […]
D:	Sì, volevo solo chiederti, il noi a cui ti stai riferendo è un noi…
R:	Di noi Comitato.
D:	Come Comitato, okay.
R:	Ripeto, perché la multidisciplinarietà e l’interdisciplinarietà sono aspetti fondamentali, perché un quesito come quello che ha posto Sogliani, l’avrebbe potuto porre direttamente a noi, a noi medicina legale, come spesso ci capita, come spesso ci capitava, talvolta ci capitava. Per cui io sarei stato in grandissima difficoltà a dare una risposta a Sogliani, lo sono anche adesso, dopo tutto questo percorso che abbiamo instaurato. Però questo è un esempio secondo me tipico, emblematico, una questione emblematica; risolvere problemi che propone la clinica comporta anche la definizione di aspetti che sono ulteriori rispetto alla mia materia, per cui ho sempre visto con grande interesse questo tipo di attività, che appunto presuppone una serie di altre, di altri punti di vista rispetto a quello che è il mio o quello che potrebbe essere quello di un clinico, o quello che potrebbe essere solo di un giurista, solo di un filosofo, solo di un… mi spiegando vero?
D:	Sì, certo.
R:	Questo è. […]
D:	Chiedo quindi a questo punto se vuoi raccontarmi un po’ qual è la tua esperienza con il CEC.
R:	In che senso?
D:	Qual è anche il livello del tipo di lavoro ecco, in che cosa consiste il tuo lavoro.
R:	Il mio lavoro soprattutto è di vedere - io credo, da come l’ho interpretato io - di vedere l’aspetto… allora noi, io faccio un mestiere, medicina legale, che è la medicina “sub specie iuris”, ovvero sotto l’ottica del diritto, cioè le questioni mediche sotto l’ottica del diritto, e io per formazione ho questo tipo di impostazione iniziale; cioè io inquadro tutti i casi medici secondo “sub specie iuris”. E il primo punto, che fa parte della metodologia che è della medicina legale, è quello del rigorismo obiettivo, ovvero di prendere i dati come sono, i fatti. Qual era la domanda che ho perso la… la mia esperienza. La mia esperienza è che questo tipo di approccio, cioè “sub specie iuris” e di analisi dei fatti, non consente di inquadrare tutto il problema, ne prende un pezzettino, una fetta, per cui è un po’ simile alla motivazione questo tipo di risposta, sta nella possibilità di confrontarsi con delle competenze diverse, non so se rispondo bene alle tue domande.
D:	No, ma non c’è mica un…
R:	Non ho un voto alla fine.
D:	Non c’è rischio di giusto o sbagliato, anzi. […] Va bene, allora ti faccio una serie di domande invece un po’ più relative al servizio e ti chiedo di dirmi qual è secondo te il ruolo che ha questo Comitato per l’Etica nella Clinica all’interno dell’azienda.
R:	Questo qua è un ruolo di tipo consultivo ovviamente, ed è consultivo io credo, l’ho inquadrata così dall’inizio, non solo sui singoli casi che vengono proposti, ma è consultivo anche rispetto a delle tematiche che esistono e sui quali si può dare un parere, con una consulenza non più sul caso che ti viene proposto ma su una questione che ritieni utile proporre tu.
D:	Ti chiedo in che modo secondo te questo servizio è diverso da altri servizi che sono proposti all’interno dell’azienda, qual è un po’ la sua specificità.
R:	Questa non l’ho capita.
D:	Secondo te…
R:	In cosa è diverso da, che so io, la consulenza chirurgica, per dire…
D:	Ma per esempio la consulenza, un pochino forse me l’hai anticipato prima, la consulenza che date voi come medicina legale oppure… cioè da altri… O se vuoi invece dirmi qual è secondo te la specificità di questo servizio.
R:	Secondo me la specificità è quella di dare delle valutazioni laddove ci siano dei conflitti, dei conflitti di valori, com’è che si chiamano, dei conflitti di valore sul tipo di attività e quindi di scelte che possono essere fatte o non fatte da un professionista. Allora, rispetto alle consulenze che faccio io, che noi facciamo praticamente una al giorno, a volte su dei casi anche veramente complicati, queste del Comitato Etico sono molto diverse, perché le mie consulenze, le consulenze che fa il mio servizio […] riguardano nella stragrande maggioranza dei casi concreti in essere e riguardano… avevamo fatto anche un lavoro io e la Strozzi che abbiamo pubblicato, riguardano per lo più questioni del consenso informato e riguardano questioni soprattutto relative al dissenso, perché il consenso non pone dei problemi, i problemi li pone il dissenso. Questioni sulle quali nella stragrande maggioranza dei casi noi siamo in grado di dare una risposta, che è quella medico legale, mica risolviamo il problema; diamo un inquadramento della questione sotto quelli che sono i profili medico legali. A volte anche in urgenza, mi chiamano di notte perché c’è un malato che ha tentato il suicidio ingerendo della candeggina e adesso rifiuta le cure; e allora che dobbiamo fare, mi chiede il chirurgo; oppure c’è il classico Testimone di Geova che non vuole essere trasfuso, adesso sono temi che tra l’altro sono stati anche un po’ superati dalla Legge 219, ma che prima della Legge 219 creavano (? 13:40) e creeranno tuttora scompiglio. Poi era tutta un’altra serie di questioni che riguardano la cartella clinica, il referto all’autorità giudiziaria, questioni molto più specifiche rispetto alla mia preparazione. Molto spesso arrivano casi, molto spesso, alcuni ve li abbiamo girati anche noi, di quelli che abbiamo esaminato al Comitato Etico, perché arrivano questioni, adesso non mi viene in mente il caso, era un medico di base che non voleva fare una terapia non convenzionale che era richiesta da un malato. Il problema che si poneva il medico non era un problema etico, era un problema di dire: ma se io non gliela faccio qualcuno mi potrà condannare per qualcosa? Cioè aveva paura delle ripercussioni giudiziarie di una sua determinata scelta; e su quel tema lì noi siamo abbastanza… noi come servizio dico siamo abbastanza tranquilli nel dare una risposta, cioè siamo in grado di inquadrare il problema senza… Però un caso di questo tipo, quello lì, proponeva anche delle altre questioni: ma è giusto rifiutare a un malato l’accesso a una terapia che non è convenzionale, eccetera? Cioè richiedeva anche un altro tipo di inquadramento, ed è proprio questo secondo me che è il valore del Comitato dell’Etica Clinica; cioè non è solo un aspetto di tipo giuridico e giudiziario, che sono quelle consulenze che chiedono a me, c’è dell’altro che riguarda l’etica proprio.
D:	Ecco, ti chiedo l’altra domanda, è un po’ qual è secondo te quindi l’obiettivo del Comitato per l’Etica nella Clinica e in che modo questo servizio agisce nel contesto locale.
```

R:	Allora, l’obiettivo secondo me prima di tutto è culturale; sì, è culturale, nel senso anche di formazione, di iniziative rivolte al personale; adesso non mi ricordo, non c’era ancora il Comitato per l’Etica Clinica, ma quando siamo andati in giro a spiegare il biotestamento, la legge sul biotestamento, noi abbiamo fatto un attività, siamo andati in giro nel senso che io sono andato ovunque, anche (*redacted*) è venuta con me, siamo andati tre o quattro a (*redacted*), uno a (*redacted*), uno a (*redacted*), siamo andati ovunque. Quella è un’attività che se ci fosse stato il Comitato per l’Etica Clinica all’epoca, sarebbe potuto essere bellissimo fatta nel contesto del Comitato dell’Etica nella Clinica. Io poi sono andato ovunque, sono andato all’ordine degli avvocati, nelle scuole, siamo andati al Comitato consultivo, ho visto dove sono i cittadini, me lo ricordo ancora quel periodo lì, perché avevamo fatto un po’ in giro delle Sette Chiese; eravamo andati… questo è un aspetto che è di tipo culturale, che credo che il Comitato per l’Etica Clinica debba avere fra i suoi principali obiettivi. Lo chiamiamo formazione per usare un termine aziendale, ma in realtà è un obiettivo culturale, cioè di accrescere le conoscenze; questo è il primo. Il secondo, cioè quello di valutazione di casi complessi è l’altro obiettivo. E questi sono i casi concreti, e questi sono i casi che ti vengono proposti dai professionisti. Il terzo è quello di iniziativa, che metto assieme a quello culturale, ma in realtà non è una formazione, quando abbiamo fatto il parere sul Covid è un parere su un tema che era sentito, ma era sentito dalla comunità sanitaria aziendale e quindi è una tematica che il Comitato di iniziativa può e deve proporlo. L’obiettivo allora lo possiamo dividere in tre: quello culturale/formativo, quello anche in questo caso culturale, però di iniziativa, e quello invece di valutazione della casistica, del caso concreto; lo possiamo dividere in tre.

```
D:	Capito. E quindi a parte quello che mi hai detto…
R:	Scusa, ma anche, io dicevo, io sono andato al Comitato consultivo misto a spiegare il biotestamento; mi sono rivolto ai cittadini, perché cioè non è solo una questione aziendal-sanitaria questo aspetto culturale, è un aspetto culturale che riguarda anche la popolazione. Adesso usiamo dei termini grossi, comunque l’opinione pubblica; adesso c’è tutta questa confusione, sedazione continua, eutanasia, suicidio e chi più ne ha più ne metta, secondo me non conoscono neanche il termine, l’unica cosa che capiscono è che a un certo punto il malato muore e tutti vissero felici e contenti; questo è un po’ quello che percepisce l’opinione pubblica. Anche dare questo tipo di informazioni con delle iniziative che non so come possono essere organizzate, potrebbe essere… potrebbe rientrare nell’obiettivo chiamiamolo culturale di cui dicevo prima.
D:	Sì, infatti era una cosa che volevo approfondire questo aspetto della cittadinanza, ma mi hai preceduto.
R:	Ti ho anticipato?
D:	Sì, in realtà volevo solamente approfondirlo perché lo trovo un aspetto molto interessante. Ti chiedo se hai qualcosa da aggiungere rispetto al modo in cui secondo te questo Comitato agisce nel contesto locale.
R:	No, io credo che dovremmo essere più visibili. Adesso che è ripresa l’attività, più visibili innanzitutto sul piano formativo; dicevo, adesso che è ripresa l’attività in presenza io credo che lì potremmo fare qualcosa di più. Potremmo fare qualcosa di più per dare maggiore visibilità, trovare degli argomenti e proporli, con delle iniziative, questo qua di tipo formativo, ma usiamo il termine formazione, ma non è solo formazione, non so, neanche accrescimento culturale, perché poi sono dei temi che magari a un diabetologo non interessano, ma a un ortopedico sì, o viceversa, è più facile che sia viceversa. Io credo che dovremmo fare qualcosa di più lì.
D:	Allora, ti chiedo invece che cosa ne pensi del valore di un servizio come il Comitato per l’Etica nella Clinica rispetto al tuo lavoro e al lavoro dei colleghi, degli operatori sanitari.
R:	Del mio servizio?
D:	In generale degli operatori sanitari, cioè dei colleghi dell’azienda.
R:	Non ho capito scusa, torna a dire.
D:	Che cosa ne pensi del valore di questo servizio rispetto a quello che è il tuo lavoro? Forse un po’ me l’hai già anticipato prima.
R:	Sì è un’aggiunta, per me, te l’ho detto anche prima esatto, per me è un valore aggiunto ecco, usiamo il termine valore aggiunto, per me è un valore aggiunto, dà di più.
D:	E rispetto invece al valore che questo servizio può avere per i professionisti dell’azienda?
R:	La stessa cosa credo, la stessa cosa. Poi è ovvio che noi facciamo un tipo di attività che a una quota di operatori sanitari interessa poco; poi a tutti dovrebbe interessare, ma ci sono di quelli che sono meno portati. Però secondo me è sempre la questione del valore aggiunto, aggiuntivo rispetto all’esistente.
D.	Ti chiedo invece adesso, ripensando un po’ a quello che è stato il percorso di questo servizio, secondo te in che modo sono state integrate le varie attività nel contesto locale, le varie attività che sono state promosse da questo servizio, pensando un po’ al contesto.
R:	Al contesto aziendale?
D:	Sì, al contesto locale, aziendale, sì.
R:	Anche insomma alla Provincia io credo, ci sono delle iniziative, adesso non mi ricordo sotto cosa è inquadrata, ma c’è quella dello sportello DAT, è inquadrata dentro il Comitato dell’Etica, quella dello sportello DAT?
D:	No.
R:	Fate parte dell’unità di bioetica; scusa ma io ragiono… sì, ma non è neanche una cosa che deve fare un Comitato per l’Etica Clinica, è vero, giustamente. Torniamo al dunque che io perdo poi il filo.
D:	Secondo te, ripensando a quello che è stato fatto in questi mesi, le attività che sono state portate avanti come sono state integrate nel contesto locale?
R:	Integrate cosa vuol dire?
D:	Come si sono diciamo così amalgamate, integrate proprio, cioè che riscontro forse c’è stato dall’altra parte.
```

R:	Questo è difficile da valutare. È difficile da valutare anche perché una volta io lavoro al (*redacted*) ed ero in mezzo alle chiacchiere, per cui sentivo… adesso sono qua al (*redacted*), ho meno occasioni, molte meno occasioni di frequentare i clinici. Però persone come, che so io, (*redacted*), anche (*redacted*), che sono sempre stati molto sensibili e che sono clinici, quindi sono gente che sì di malati, cioè di robe concrete, con loro credo che l’integrazione sia sempre stata molto efficace; mi vedo meno con altre componenti, perché non ho più un ritorno soggettivo, non ho più un riscontro, la chiacchiera, come posso dire, la…

```
D:	Informale?
R:	Ecco, brava, chiacchiera informale. Io credo che su questo punto qua, promuovendo delle iniziative varie e svariate, si crei anche un substrato; cioè io credo che dovremmo, torno a dire, una maggior visibilità all’interno dell’azienda che si ottiene così, con la pubblicità di fatto, perché è un promuovere sé stessi. Insomma, noi ci siamo… ci occupiamo di questa cosa, ma anche di quest’altra; adesso mi viene in mente che uno o due anni fa mi hanno fatto una serie di questioni sull’obiezione di coscienza nell’aborto io dicevo ma porca miseria, ma sono quarant’anni che c’è una legge, siamo ancora qua a parlare di obiezione di coscienza? E invece sono temi che ogni tanto ricorrono, ritornano. E questi potrebbero essere degli argomenti; torno a dire, avere maggiore visibilità credo che dovrebbe essere un nostro obiettivo ecco, che favorisce poi tutto il resto, integrazione. Il tema credo anche che noi quando diamo una risposta è la velocità di risposta; anche su questo occorrerà riflettere, cioè il tempo di risposta.
D:	No assolutamente è un elemento importantissimo. il tempo della risposta è…
R:	No, perché guardi, torno a dire, riportando la mia esperienza, che faccio delle consulenze che in qualche modo hanno certi aspetti così, non rispondiamo subito al telefono; cioè diamo un’indicazione, inquadriamo un problema o diamo anche un’indicazione, lo facciamo subito. A volte c’è bisogno di fare delle riunioni e gli incontri e quindi le cose vanno un po’ più per le lunghe; nella stragrande maggioranza dei casi io rispondo immediatamente, domanda A, io rispondo B, punto. Quando sono capace.
D.	Ecco, rispetto a questo, questo mi fa venire in mente la prossima domanda che ti volevo fare, è appunto se secondo te ci sono dei limiti o delle criticità che percepisci rispetto al Comitato per l’Etica nella Clinica.
R:	Questo però non credo che sia una criticità, è un dato di fatto; io credo anche che un Comitato, che è composto da quindici, dodici, quindici persone non possa dare una risposta in giornata e nemmeno in due o tre giorni dopo; per cui è nell’essere stesso un Comitato che si fa fatica a dare una risposta di questo tipo. No, io delle criticità vedo molta competenza del Comitato per l’Etica, vedo molta competenza, non vedo delle criticità. Quella della tempistica della risposta poi varia e può variare, però ripeto è nella natura stessa dell’essere un comitato che ti impedisce e non ti consente di dare dei feedback immediati o con poco tempo, è nella natura stessa, per cui non credo neanche possa essere interpretata come criticità. Non vedo altre, io vedo molta competenza.
D:	Ti chiedo questa domanda, ma più a scopo personale, quindi se secondo te, cioè questo elemento della tempistica potrebbe avere una ripercussione proprio sul servizio di consulenza che offre il Comitato, cioè magari essere percepito come limitata.
```

R:	Allora può essere… allora è ovvio che un collega che ti chiede un parere vuole una risposta in tempi brevi; ma già le facciamo anche con (*redacted*), quando ci chiedono i pareri spesso di tipo neonatologico o pediatrico, diamo una risposta nell’arco di poco, giorni, qualche giorno. Di solito ci mandano delle relazioni che sono da mettere a posto e diamo una risposta quasi subito, ma non è un tema da Comitato per l’Etica della Clinica quello lì, mi sto spiegando? Cioè è nella natura stessa di essere un Comitato multidisciplinare, l’impossibilità di dare una risposta; è un limite del Comitato stesso, ma è nella sua natura insomma.

```
D:	Invece ti chiedo se hai qualcosa da aggiungere rispetto ai punti di forza che secondo te ci sono in questo Comitato.
R:	Il punto di forza è la multidisciplinarietà e la competenza dei componenti; su questo non ho niente da dire, niente da aggiungere.
D:	Allora io ti faccio le ultime due domande, fondamentalmente una: ti chiedo come valuti rispetto appunto anche al percorso che c’è stato, pensando alle fasi iniziali e pensando invece a oggi, a distanza di venti mesi più o meno, come valuti un po’ questo servizio che è stato implementato.
R:	Allora noi abbiamo avuto la sfiga che in mezzo abbiamo avuto il Covid, che ha alterato tutte le attività compresa la nostra, perché è ovvio; questo è un dato circostanziale di certezza; dicevi come valuti…
D:	Quello che è stato un po’ il percorso anche di questo servizio, ripensando alle fasi iniziali, pensando a come è oggi, un tuo giudizio diciamo così complessivo su questo servizio che è stato implementato.
R:	Non so come risponderti a questa cosa. Un giudizio in senso favorevole/sfavorevole, positivo/non positivo, che ne so?
D:	Un tuo parere.
R:	Allora io sono soddisfatto dell’esistenza di questo servizio, uno perché è, usiamo un termine, un fiore all’occhiello dell’azienda USL di Reggio, visto che servizi di questo tipo non ce n’é in altre aziende, quindi è un fiore all’occhiello. E quindi io di questo sono molto soddisfatto. Poi non mi viene in mente altro.
D:	Va bene così, ci mancherebbe.
R:	No, perché ho detto uno e poi ho detto, il due qual era? Non mi viene in mente altro.
D:	Va bene, qualsiasi tua idea, opinione, riflessione è assolutamente giusta, nel senso che…
```

R:	Due era, scusami mi è venuta in mente il due, che è l’aspetto regionale che diceva (*redacted*); questo è un riconoscimento che non è che sia una roba da poco; adesso non so bene il termine, però è un… siccome so che è stato detto anche in Direzione, la Regione ha fatto presente alla Direzione, che quindi è vissuto anche dal punto di vista della Direzione come un fiore all’occhiello, ho usato questo termine che ripeto, è un aspetto anche questo che per me è fonte di grande soddisfazione. Era questa la domanda quali sono le tue… Quindi l’aspetto è anche questo chiamiamolo riconoscimento a livello regionale, è un aspetto di cui tenere grande conto.

```
D:	Okay. L’ultima domanda che ti volevo fare, ma mi hai veramente risposto ampiamente prima, era in che modo secondo te questo servizio può essere migliorato.
R:	Noi dobbiamo essere più visibili, io credo che sia il punto centrale; l’obiettivo di miglioramento può essere maggiore visibilità.
D:	Basta, io ho concluso le mie domande, ho concluso di disturbarti con queste domande, l’ultima cosa che ti chiedo è se rispetto a questo servizio, rispetto anche un po’ all’obiettivo che è questo progetto, che è quello di capire un po’ com’è andato questo servizio e darne una valutazione, se hai qualche altra cosa che ti va di condividere, che magari non è emersa da questa intervista.
R:	No.
D:	Okay.
R:	Non mi viene in mente niente.
D:	Va benissimo. Va bene, allora abbiamo concluso.
R:	Va bene.
Fine trascrizione 00:36:34
```
