# Supplementary material for: How to implement a clinical ethics committee in an oncological research hospital: Qualitative results from a process evaluation study using normalization process theory (EVACEC)
Source: PLoS One. 2025 May 6;20(5):e0318870. doi: 10.1371/journal.pone.0318870 (PMC12054913; doi:10.1371/journal.pone.0318870)
Supplement: S5 File — Interviews transcripts_raw data: this folder contains all the raw data used in this study, the interview transcripts (in original Italian). (ZIP) [file pone.0318870.s005.zip › S5_Interviews transcripts_raw data/07CM.doc]

```
R: 	Eccomi.
D:	Perfetto. Allora, la prima domanda che… le prime due domande che ti faccio sono domande un po’ generali e la prima riguarda un po’ quella che è diciamo così la tua esperienza rispetto se ci sono state delle formazioni precedenti rispetto al tema dell’etica.
```

R:	No, dunque, io formazione specifica non ne ho ricevuta, però sono stato componente di diversi comitati etici; sono stato componente di tre comitati etici (*redacted*)… non mi ricordo, (*redacted*) probabilmente; poi sono stato componente del comitato etico (*redacted*) solo per sei mesi, prima che venisse istituito il Comitato Etico dell’AVEN, poi ero stato nominato per il Comitato Etico dell’AVEN ma mi sono dovuto dimettere il primo giorno perché avevano fatto male i conti rispetto a esterni e interni, eccetera, mi hanno chiesto la cortesia di farmi da parte, per cui… No… quindi diciamo che la mia esperienza è soprattutto di partecipazione a Comitati per le sperimentazioni, poi naturalmente un po’ di letteratura, quindi di autoformazione, ma non tanto, nel senso che poi soprattutto nella pratica del Comitato, cercando di applicare un po’ alcuni concetti relativi così all’importanza dei conflitti di interesse negli studi di sperimentazione insomma. Ovviamente questo è un tema abbastanza sensibile e critico così come gli studi osservazionali che spesso e volentieri sono utilizzati dalle ditte per promuovere dei loro prodotti, con la scusa tra virgolette di fare ricerca, in qualche modo si cerca di indurre la prescrizione di determinati farmaci. Quindi questi temi qui, insieme naturalmente ad altri, all’importanza del consenso informato che sia informato davvero e non quelle lenzuolate di pagine con caratteri alla Zio Paperone che non ti fanno capire niente. Quindi un po’ di sensibilità su tutte queste cose in qualche modo ho cercato di svilupparla nel tempo ecco; però la mia esperienza sì, è stata questa. Poi naturalmente con la proposta vostra di far parte di questo Comitato che ha delle caratteristiche ovviamente un pochettino diverse, ma neanche tanto, ma insomma perché è sempre l’etica giustamente è la parola chiave che accomuna le due cose. Per cui insomma è un punto di vista un po’ diverso, non orientato a valutare l’eticità delle sperimentazioni e la difesa dei pazienti per quanto riguarda la loro inclusione in progetti di ricerca, ma quindi dal punto di vista un pochettino diverso, ma anche questo estremamente importante, interessante dal punto di vista intellettuale, e quindi diciamo che sono ben contento di aver accettato questo invito e direi che insomma ogni seduta e ogni tema che viene proposto è fonte di riflessione, è fonte di discussione e confronto insomma, che penso che sia importante a livello dell’azienda sanitaria e a livello appunto degli obiettivi che il Comitato ha e penso che sia veramente molto stimolante e arricchente e che può far crescere me sicuramente, ma anche penso un po’ tutti quanti i membri del Comitato.

```
D:	Infatti volevo un po’ chiederti anche qual è la tua… se mi vuoi raccontare ecco la tua esperienza proprio con il Comitato per l’Etica nella Clinica.
R:	Con il nostro, con un Comitato, nel senso che il nostro è l’unico a cui ho partecipato, anche perché mi sembra di capire che ce ne siano ben pochi ancora in Italia con queste caratteristiche qui. No, appunto, confermo quello che dicevo prima, cioè l’esperienza è un’esperienza positiva, perché mi permette di riflettere su appunto aspetti dell’etica che non sono esattamente gli stessi rispetto al Comitato per le sperimentazioni e la ricerca, ma che comunque sono appunto più vicini ai bisogni e ai problemi dei singoli pazienti, così come dei singoli clinici, quindi tutto ciò che può in qualche modo supportare e aiutare nel capire quali possono essere i risvolti etici di determinate scelte e possono essere scelte di sistema, ma possono anche essere scelte appunto di singoli professionisti e di singoli pazienti e quindi che comunque vanno gestiti anche in termini di… anche per i risvolti appunto che hanno sul fronte dell’etica ecco. Per cui esperienza positiva, assolutamente positiva insomma.
D:	E quali sono state le motivazioni che ti hanno spinto a prendere parte a questo servizio?
```

R:	È stata (*redacted*) che mi ha cercato, che mi ha convinto; dal mio punto di vista insomma sicuramente la curiosità e comunque la voglia di rendermi utile sono state le leve che mi hanno spinto ad accettare questo invito. Credo che sia una cosa arricchente, quindi questo lo avevo immaginato, e l’idea di imparare cose nuove, anche attraverso il confronto con i colleghi, è stata sicuramente una spinta importante, così come appunto è stata una spinta importante l’idea di potermi rendere utile rispetto a una richiesta che mi è venuta e che quindi… io naturalmente a (*redacted*) ho fatto subito presente che appunto la mia formazione scientifica come farmacista, che peraltro appunto non è clinico e quindi non ha il contatto con i pazienti e la responsabilità di prendere delle decisioni per i pazienti. Quindi ovviamente ho cercato subito di inquadrarmi in questo senso per dire: valuta un attimo anche se secondo te io posso essere un valore aggiunto. Poi effettivamente a pensarci, cioè mi sono sentito naturalmente di farle presente questo ecco, perché per stimolarla a essere molto convinta della nostra scelta. Effettivamente insomma comunque i temi, il tema dell’etica rispetto anche ad argomenti che possono essere più strettamente legati alle terapie farmacologiche, cioè ci può essere una stretta connessione, una necessità di approfondimento sui risvolti etici anche da questo punto di vista, dal punto di vista dell’utilizzo di terapie come insomma abbiamo ampiamente visto le ultime volte sull’utilizzo delle terapie farmacologiche, così come per quanto riguarda la politica del farmaco in generale. Risvolti etici che determinate cose che hanno a che fare, per esempio, con conflitti di interesse da parte dei produttori, che hanno dei risvolti naturalmente sui farmaci che sono messi in commercio, sulle modalità di messa in commercio dei farmaci, sulle scelte che quindi possono a valle essere fatte dalle aziende che acquistano i farmaci e dai clinici che li utilizzano. Quindi ci sono degli aspetti etici che magari ecco non sono venuti fuori fino a ora in questo modo, ma che comunque possono essere importanti anche, potrebbe essere importante analizzarli. Poi naturalmente ci sono le situazioni che riguardano i singoli casi, che hanno a che fare anche con la scelta di terapie, cioè con la scelta di terapie e anche con la scelta di fare qualcosa (? 09:59) fare.

D:	Va bene, allora adesso invece ti farò una serie di domande più specifiche proprio sul servizio e su tutto il processo che c’è stato diciamo e partirei col chiederti qual è secondo te il ruolo del Comitato per l’Etica nella Clinica all’interno dell’azienda.

R:	Come abbiamo potuto vedere soprattutto con le ultime cose, si ipotizzava addirittura che il CEC potesse essere in Emilia-Romagna l’organo di supporto decisionale alle scelte sul suicidio assistito, per cui diciamo che il ruolo che può svolgere il CEC potrebbe in parte derivare da scelte normative che non sono ancora ben definite, ben chiare. Però sicuramente, al di là di questo, al di là quindi di un ruolo normato, può essere… stiamo vedendo quanto importante possa essere avere un gruppo, un Comitato insomma a cui anche singoli professionisti si possano rivolgere per dubbi, situazioni che hanno dei risvolti in termini di etica importanti. E quindi un’azienda che mette a disposizione dei propri professionisti e anche dei propri cittadini un Comitato siffatto insomma è sicuramente un’azienda illuminata, che ha anche avuto la fortuna di evidentemente trovare persone, (*redacted*), immagino che anche la direzione scientifica del IRCCS abbia insomma avuto un ruolo nel promuovere questa cosa e quindi insomma di creare le condizioni affinché un simile Comitato potesse essere impiantato. Per cui è una scelta sicuramente illuminata, da parte di un’azienda che nell’organizzare i servizi sanitari a livello del territorio, quindi non si preoccupa soltanto degli aspetti organizzativi in senso stretto e clinici in senso stretto, ma anche appunto di tutto ciò che può aiutare a far girare la macchina al meglio e quindi anche ovviamente un Comitato per l’Etica può essere di grande aiuto e di supporto, come dicevo prima, sia ai singoli professionisti che anche i cittadini.

```
D:	La prossima domanda in realtà insomma mi è abbastanza chiara da quello che mi avevi raccontato prima, quindi ti chiedo se vuoi aggiungere magari qualcosa rispetto a questo, in che modo secondo te è diverso questo servizio da altri servizi promossi appunto dall’azienda, qual è un po’ la sua specificità, anche in termini di obiettivi, per esempio.
R:	L’unico confronto che mi può venire in mente è rispetto al Comitato per le sperimentazioni, non so se intendevi quello come altri servizi. Sì, quello che ho detto prima, che appunto i due hanno ovviamente dei punti di contatto importanti, visto che si tratta di etica nell’uno e nell’altro caso. Però il Comitato per l’Etica nella Clinica integra sicuramente in modo importante gli aspetti dell’etica che possono essere trattati anche o maggiormente dall’altro Comitato, ponendosi a supporto dei singoli professionisti e dei cittadini rispetto a tematiche generali che non riguardano uno studio e quindi l’eticità di uno studio e la difesa dei pazienti cittadini che possono appunto essere invitati a partecipare a quello studio, ma si tratta appunto di approfondire delle situazioni anche singole, anche legate appunto a singoli episodi, singole persone, come abbiamo visto insomma ampiamente nelle ultime settimane, negli ultimi mesi e comunque da quando il Comitato per l’Etica nella Clinica esiste. Ci siamo sempre occupati di situazioni particolari, di quesiti che vengono appunto dal territorio, ma singoli professionisti, e quindi un approfondimento, un ragionamento su questo può essere di supporto a loro, può essere di riflesso all’azienda, può essere di supporto anche a noi e soprattutto a quei professionisti, in particolare i clinici che fanno parte del Comitato, che possono appunto aiutare anche sé stessi ad affrontare delle situazioni simili, avendo avuto la possibilità di ragionarci su e di confrontarsi con il resto dei colleghi del Comitato. Per cui sicuramente è un’integrazione, quella del Comitato per l’Etica nella Clinica rispetto al Comitato per le sperimentazioni. Ripeto, poi secondo me il problema non so se diciamo vado un po’ fuori tema o anticipo domande che potrebbero esserci. No, ma il problema secondo me, che io mi sento molto, è chiaro che le scelte di un Comitato per l’Etica nella Clinica o i giudizi che possono essere espressi risentono molto dell’opinione dei singoli e della composizione del Comitato stesso; non è detto che un Comitato per l’Etica nella Clinica su un altro territorio, un altro Comitato sullo stesso quesito può rispondere allo stesso modo. Però il tema della variabilità, dell’assistenza, e questi sono temi comunque assistenziali in ogni caso. Perché un parere su cose di cui ci siamo occupati recentemente riguarda comunque il tipo di assistenza che è giusto e lecito e opportuno fornire; che uno che abita a Canicattì e uno che abita a Bolzano, oppure che uno che abita a Roma e uno che abita a Firenze, insomma possano essere trattati in modo diverso in base a giudizi che possono essere molto variabili, è una cosa che secondo me andrebbe affrontata in qualche modo. Cioè andrebbe affrontata, per esempio il tema del fine-vita è chiaro che non essendo adeguatamente normato lascia molto spazio alle interpretazioni, però insomma ecco, secondo me il Comitato per l’Etica nella Clinica potrebbe fungere… potrebbe avere una funzione importante nel rimarcare questo aspetto qui, cioè che l’assetto normativo per quanto riguarda questi temi qui è ampiamente carente e dà adito a delle interpretazioni che possono essere variabili all’interno dello stesso Comitato, quindi figuriamoci poi se andiamo a considerare diversi Comitati. Però sì, non è facile ovviamente e non penso a una standardizzazione in base a regole rigide di quelli che possono essere i pareri, però forse il tema dell’eccessiva variabilità che nell’assistenza sanitaria è atavico in Italia naturalmente, motivo per cui tanta gente poi viene al Nord a farsi curare. Però anche rispetto a dei pareri, delle scelte, come quelli che possono essere sul fine-vita insomma forse bisognerebbe anche da parte nostra cercare di confrontarci anche con altri, per cercare di attivare dei ragionamenti comuni che possano in qualche modo portarci a conclusioni simili e che non rischino di essere a volte possibilmente opposte insomma, perché naturalmente dipende un po’ dalle sensibilità dei membri di ciascun Comitato.
D:	Una variabilità più che altro appunto esterna, cioè è una variabilità che tu hai riscontrato rispetto all’esterno, non tanto all’interno, ho capito giusto?
R:	No, faccio un ragionamento molto speculativo, cioè non ho elementi per dire… non ho altri pareri di altri comitati per dire noi abbiamo ragionato in modo diverso, però immagino che questo è molto facile che avvenga nel momento in cui all’interno del nostro stesso Comitato ci possono essere dei ragionamenti un po’ eterogenei su queste cose, per cui a maggior ragione immagino che in altri Comitati, che magari potrebbero essere anche espressione di altre sensibilità, che poi possono essere anche riflesso di diversi assetti oserei dire anche politici, perché naturalmente poi può dipendere anche dal tipo di assetto politico all’interno di una Regione quindi che già porta. può facilitare o non facilitare il fatto che si faciliti, si abbia un Comitato per l’Etica nella Clinica. E che poi quel Comitato per l’Etica nella Clinica ha naturalmente dei componenti, la nomina di questi componenti può in qualche modo riflettere anche determinati orientamenti e quindi di conseguenza anche le decisioni prese possono essere figlie di tutto ciò.
D:	Chiaro. Ecco, rispetto a questa domanda, appunto una delle domande che ti volevo fare era appunto se ci sono stati dei limiti o se tu percepisci dei limiti rispetto a questo servizio a livello per esempio di organizzazione, se invece ci sono stati dei limiti interni, esterni; se hai qualcosa da aggiungere rispetto a questo.
R:	No, limiti no, direi forse solo i limiti normativi di cui dicevo prima rispetto a determinate cose, in particolare rispetto al fine-vita c’è una situazione ancora troppo vaga, che accentua molto diciamo la soggettività delle interpretazioni. Quindi il limite ma rispetto a un tema ben definito, poi per il resto è anche vero che forse una certa non standardizzabilità e non rigidità dei giudizi può essere anche insita in un Comitato per l’Etica nella Clinica ecco. L’importante è che non diventi un qualcosa che vi porti… possa portare a delle interpretazioni molto diverse, magari per una situazione simile ci possono essere delle opinioni molto diverse all’interno del Comitato e nel confronto tra decisioni. Per cui secondo me il confronto anche con altri Comitato per l’Etica nella Clinica per attraverso proprio… il confronto è sempre arricchente, per cui attraverso il confronto si possa ragionare sui punti che possono essere più o meno condivisibili da tutti, i punti più controversi e magari essendo più controversi quelli che potrebbero meritare un approfondimento anche con gruppi di lavoro e anche poi financo utilizzare tutto questo lavoro per sensibilizzare chi a livello normativo potrebbe insomma aiutare a chiarire meglio determinate situazioni, anche attraverso audizioni parlamentari. Insomma, più si è e più si ha forza anche per promuovere determinati temi a livello politico proprio.
D:	Chiaro. Invece dei punti di forza che ti vengono in mente di questo servizio?
R:	Un po’ quello che dicevo prima, cioè fatto che esistendo un Comitato per l’Etica nella Clinica è un qualcosa che permette di cercare delle risposte per situazioni fino alle scelte dei singoli clinici o dei singoli cittadini o comunque apporre dei problemi che sono anche più generali e più comuni. È un qualcosa che almeno da noi prima non c’era, ma mi sembra di capire che un po’ in tutta Italia sia poco presente, e lo era a maggior ragione poco presente prima, quindi è un valore aggiunto che integra le cose che possono essere discusse all’interno del Comitato per le sperimentazioni, aggiunge quel qualcosa che ovviamente nel Comitato per le sperimentazioni che ha il focus sugli studi, sulla ricerca e sulle sperimentazioni e sulla difesa dei pazienti, cittadini che potrebbero essere invitati appunto a partecipare a queste sperimentazioni. Qui invece il focus è diverso, l’etica è sicuramente il denominatore comune, però andiamo appunto su un approfondimento dei temi etici che può riguardare anche delle situazioni, di singole situazioni particolari, che hanno naturalmente anche dei riflessi a livello generale, cioè non sono necessariamente situazioni che riguardano necessariamente solo una o poche persone, possono riguardare tanti ma che comunque insomma esempi portati alla conoscenza possono insomma essere utili per affrontare i problemi che possono poi essere più generali.
D:	Okay, allora l’ultimissima domanda che ti faccio è una domanda un po’ conclusiva rispetto a una tua valutazione diciamo generale su tutto un po’ il processo, su quello che è stato fatto un po’ dall’inizio di questo Comitato fino a oggi e appunto una tua valutazione su come è stato un po’ questo processo, questo sviluppo di questo servizio; se ci sono magari anche delle cose da migliorare, quindi una tua opinione proprio conclusiva su questo servizio.
```

R:	L’opinione è molto favorevole, molto positiva, non saprei cosa consigliare di più; a parte forse - ma mi sembra che voi lo facciate già abbastanza - quello di anche essere un po’ in rete con altri Comitati, sentire anche altri. Chiaro che i membri del Comitato per l’Etica nella Clinica - penso a me ma naturalmente a tutti quanti gli altri colleghi - non necessariamente hanno anche il tempo per approfondire tante cose e per insomma attivare dei gruppi di lavoro sia interni che anche di confronto con l’esterno. Idealmente sarebbe una gran bella cosa se si potessero attivare i gruppi di lavoro in collaborazione con altri Comitato per l’Etica nella Clinica per valutare come affrontare determinate situazioni, avendo un approccio comune per quello che si può naturalmente. Quindi questa sarebbe una… potrebbe essere una bella cosa, non facile però, perché tutti quanti naturalmente siamo pieni di cose da fare, per cui non è semplice. È chiaro che poi c’è qualcuno che ti mette su un piatto d’argento e che fa un grosso lavoro di background, di organizzazione per facilitare questo tipo di cose, il tempo magari si riesce a trovare più facilmente. Però mi rendo conto che è complicato e comunque è già un grosso risultato quello di avere un Comitato per l’Etica nella Clinica che funziona bene intanto nella realtà, che è un punto di supporto per clinici, cittadini e pazienti insomma che abbiano requisiti specifici e quindi considerando anche che siamo in una fase piuttosto iniziale, non è che abbiamo una storia decennale alle spalle, piano piano insomma si potrebbe anche considerare di estendere il raggio di azione, nei limiti del possibile, attraverso l’attivazione anche di gruppi collaborativi e inter comitati per avere… affrontare un po’ determinate cose. Però insomma mi sembra che questa cosa stia già nelle corde e sia già stata avviata. Capisco che (*redacted*) insomma, voi in particolare siete molto in contatto, in connessione con altri; un convegno l’avete fatto che quindi aveva effettivamente questo tipo di idea e quindi non sto a dire effettivamente nulla di nuovo.

```
D:	Bene, okay. Allora questa era appunto l’ultima domanda, ti ringrazio davvero per insomma questa condivisione.
R:	Spero che sia stata utile e di non aver detto cose troppo banali insomma.
D:	Assolutamente, ma ci mancherebbe, no, anzi queste interviste vengono fatte proprio per capire il pensiero di chi ci lavora, che è un pensiero utile e necessario per capire appunto cosa è stato fatto, come è stato fatto e per avere dati per insomma andare avanti su questo servizio, migliorarlo, modificarlo, mantenere le cose che funzionano bene. Quindi no, assolutamente utilissimo. Quindi niente, ti chiedo se come ultimissima cosa c’è qualcos’altro che ci tieni a condividere, rispetto a questo servizio, rispetto alla tua esperienza, prima di chiudere l’intervista, qualcosa magari che non è emerso durante questa intervista?
R:	No, no direi che insomma ho anche fatto abbastanza voli pindarici all’interno dell’intervista, proprio per dire al di là adesso delle domande che mi hai rivolto insomma alcune cose, poche ma che comunque possono essere anche utili da condividere. No non vi viene in mente nient’altro.
D:	Benissimo, va bene. Allora ti ringrazio ancora tanto per il tempo dedicato.
R:	Grazie a voi.
D:	Ci sentiamo prossimamente, ci vedremo prossimamente.
Fine trascrizione 00:32:11
```
