# Supplementary material for: How to implement a clinical ethics committee in an oncological research hospital: Qualitative results from a process evaluation study using normalization process theory (EVACEC)
Source: PLoS One. 2025 May 6;20(5):e0318870. doi: 10.1371/journal.pone.0318870 (PMC12054913; doi:10.1371/journal.pone.0318870)
Supplement: S5 File — Interviews transcripts_raw data: this folder contains all the raw data used in this study, the interview transcripts (in original Italian). (ZIP) [file pone.0318870.s005.zip › S5_Interviews transcripts_raw data/08CM.doc]

```
D: 	Allora, intanto ti ringrazio per la disponibilità a partecipare all’intervista. Intanto ti chiedo se per caso ci sono dubbi sullo studio, domande, è tutto chiaro?
R:	Tutto chiaro.
D:	Tutto chiarissimo, bene. Allora, intanto ti chiederei come domanda preliminare se mi racconti qualcosa della tua esperienza con il lavoro del CEC.
```

R:	Allora, la mia esperienza con il CEC è contemporaneamente alla sua stessa formazione; quindi sono stato contattato direttamente dall’attuale Presidente, quindi da (*redacted*), per sondare la mia disponibilità, oramai a diciotto mesi fa quasi, di essere un membro di questo nuovo Comitato, e ho aderito diciamo subito con grande entusiasmo, perché mi interessano molto i problemi di etica clinica e non c’è posto migliore per vedere i problemi di etica clinica che dentro le strutture che si occupano appunto di assistenza o comunque che prestano appunto dei servizi a dei pazienti o alla cittadinanza. Quindi ho aderito subito con grande entusiasmo e dopodiché sono stato coinvolto in diversi tipi di attività che hanno riguardato più in generale la missione delle CEC. Direi tre attività in particolare: la prima è la partecipazione ovviamente alle riunioni, che sono sempre state molto stimolanti; la seconda sono stati i feedback ai vari documenti, che soprattutto sono stati realizzati nella prima fase e che ora io credo non realizziamo più per motivi, secondo me, anche ragionevoli, nel senso che il tempo e le risorse sono forse meglio investiti in un momento come questo su altri fronti. E tre, sul lato della didattica perché ho partecipato alla prima edizione, come docente relatore, del corso che è stato fatto proprio per cercare di promuovere l’attività e la conoscenza del CEC agli operatori sanitari di Modena e Reggio Emilia.

```
D:	Benissimo. Secondo te, qual è l’esperienza del ruolo del CEC? Scusami, qual è il ruolo del CEC all’interno dell’azienda, quindi dell’ospedale di Reggio Emilia?
R:	A mio avviso e per dire cioè quella che è la mia opinione […]. Allora, secondo me il CEC rappresenta uno strumento molto importante all’interno di una struttura come quella appunto insomma dell’azienda sanitaria di Modena e Reggio Emilia, perché permette di affiancare un indispensabile servizio, che è quello già svolto dall’unità di bioetica in forma più puntuale, ma di integrare questo stesso servizio di supporto su questioni che hanno ovviamente delle implicazioni di etica, bioetica, in parte forse anche di biodiritto, a un livello differente, a un livello interdisciplinare e a un livello che consente di astrarre dal coinvolgimento personale che invece necessariamente chi fa parte dell’unità di bioetica sempre ha quando viene poi chiamato e chiamata a seguire i vari casi. Quindi offre una sorta di spazio di riflessione multidisciplinare e interdisciplinare, che consente ai professionisti sanitari che lo conoscono e ne hanno capito la soluzione di poter accedere per avere, come dire, uno spazio di approfondimento su questioni di due tipi, cioè essenzialmente sempre problematiche con risvolti di etica, ma questioni che magari sono di due tipi, perché sono o nuove o semplicemente non c’hanno mai pensato. Io sono dell’idea che il 70-80%, forse di più, dei problemi di etica clinica non è mai nuovo, sono semplicemente problemi che si presentano in qualche aspetto nuovo che li rende non più risolvibili all’interno dei quadri o delle prassi che c’erano fino a prima. Questo non vuol dire che le prassi di prima fossero giuste, la maggior parte delle cose veniva risolta in maniera abitudinaria; il CEC è invece l’opportunità di creare uno spazio istituzionalizzato di riflessione, che consente di trattare questi problemi non solo in maniera abitudinaria, perché il primario di quel reparto ha sempre fatto così, ma appunto di offrire un tipo di spazio che consente anche di fare un’altra cosa secondo me importante, cioè di interagire con degli esperti che non fanno parte necessariamente della stessa struttura di appartenenza, quindi in maniera libera da quei possibili conflitti di interesse, di gerarchia che invece necessariamente ci sono quando ci si appoggia a servizi che sono strutturati all’interno della stessa gerarchia.
D:	Questo aspetto secondo te emerge particolarmente nel CEC, cioè si riesce a eliminare effettivamente questo tipo di dinamica?
R:	Allora, io posso semplicemente dare la mia opinione dall’interno del CEC; secondo me all’interno del CEC sì, e questo un ottimo risultato che è stato compiuto e diciamo così anche per serendipità. Ma dipende anche dalla selezione dei partecipanti a monte. Cioè io credo che all’interno dei lavori del CEC, malgrado le differenti esperienze, malgrado il differente peso professionale che ricoprono le diverse persone, perché naturalmente, voglio dire, né i dottorati in filosofia e bioetica, rispetto all’esperienze di una persona che ha fatto quarant’anni in reparto di cure palliative hanno un peso differente. Ma hanno un peso, questo lo dico perché ne sono convinto, per alcuni aspetti, per risolvere alcuni problemi non necessariamente meno importanti, ha un peso diverso. Per cui il successo di un’iniziativa come questa sta nel superare queste che sono delle gerarchie, che magari sono comunque evidenti dal punto di vista vis a vis insomma, nel rapporto professionale, anche per quanto riguarda l’età, ci sono persone che sono alla fine della loro carriera lavorativa, io sono diciamo verso la metà, mettiamola così. E poi anche questi fattori comunque pesano a volte nelle interazioni, invece secondo me nel CEC questo siamo riusciti a superarlo molto bene, c’è un confronto molto rispettoso, molto aperto e molto disponibile da parte di tutti i membri, a prescindere da questi fattori. Secondo me all’interno della discussione sì, come questo viene poi recepito e percepito dall’esterno e da chi ci si rivolge non lo so.
D:	Va bene, grazie. E secondo te, anche se in parte mi hai già risposto a questa domanda, però ti chiederei se in caso se senti di dover aggiungere qualcosa, secondo te in che modo il CEC, cioè il servizio che offre il CEC è diverso da altri servizi promossi e qual è la sua specificità?
R:	Allora, qui dovrei ricordarmi le slide che ho fatto, il discorso se era su questo magari dico una cosa diversa, ma comunque… in parte sì, ho già risposto e secondo me sono questi i profili; da una parte c’è un rapporto personale, quindi l’unità di bioetica, dall’altro qui c’è un rapporto istituzionalizzato. Da una parte c’è una consulenza e un tipo di interazione puntuale, immediata e continuativa, dall’altro lato invece c’è una modalità mediata, temporalizzata, perché ci sono delle scadenze, e collettiva. Quindi secondo me ecco, questi sono i profili principali; cambia proprio la modalità di affrontare, interpretare i problemi, di dare una visione risolutiva sui problemi. E poi naturalmente c’è una questione che riguarda la definizione stessa dell’utilità dei due servizi; mentre da una parte servono per risolvere il problema mentre il problema esiste, il CEC non necessariamente può essere uno spazio per capire quello che è successo o anticipare cose nel futuro o nel passato; mentre invece l’unità di bioetica, da quanto è la mia percezione, è più ancorata alla dimensione del presente, mentre il CEC si rivolge più al passato e al futuro. Quindi sono servizi, a mio modo di vedere, complementari.
D:	Secondo te qual è l’obiettivo del CEC e in che modo agisce il CEC nel contesto locale? Anche qua qualche cosa hai già detto, ti chiedo se hai altri elementi da aggiungere.
R:	Allora, sì, direi che gli obiettivi del CEC sono quelli che abbiamo appena detto, cioè quelli di offrire uno spazio di riflessione e la possibilità di un confronto mediato, interdisciplinare su questioni inerenti l’etica, la bioetica, in parte il biodiritto, a cui di solito i professionisti sanitari non sono formati. Quindi il CEC consente di on demand accedere a un’expertise e a un confronto tra diverse expertise che altrimenti non sarebbe disponibile per professionisti sanitari. E secondo è questo che deve fare, cioè è lì che (? 09:10) hanno raggiunto, nel fatto che io non devo per forza fare io tutta la mia vita i corsi di bioetica o di cure palliative, perché è impossibile, ma esiste un organismo di professionisti già formati, con una certa expertise, che si relazionano al mio caso particolare, perché io ho fatto una richiesta e quindi comunque ho un ritorno nel mio linguaggio e nelle mie modalità, perché poi ricevo io la risposta, ma questa risposta è creata da più menti assieme e l’expertise che crea questo tipo di risposta, di consulenze, di interazione, a seconda dei casi, è qualche cosa che non è recuperabile da ogni… da un singolo, sarà una cosa necessariamente collettiva. Secondo me lì è il valore aggiunto del CEC e più riesce a fare la differenza su questo profilo, più mostra di essere utile.
D:	Certo. Invece, rispetto a te quali sono le motivazioni che ti hanno spinto a partecipare alle attività del CEC, quindi diventare un membro del CEC?
```

R:	Allora, la prima motivazione è proprio perché mi interessa l’equipe clinica; mi interessa molto da professionista in bioetica, perché credo che sia una delle dimensioni fondamentali dell’expertise bioetica contemporanea. Non necessariamente tutti devono avere questo tipo di esperienza, per quanto riguarda la mia formazione invece è un profilo molto importante. E quindi questa sicuramente è un’esigenza di espressione professionale e di formazione allo stesso tempo, da parte mia. Il secondo motivo è per la qualità degli altri membri, in primis della Presidente, che conoscono da poco tempo, più nella mia frequentazione CEC, ma che ritengo essere – e questo lo dico non perché stiamo facendo l’intervista qui – ma una professionista giovane, molto determinata, molto brava e molto preparata e io credo che bisogna – lei lo sa benissimo, ne abbiamo parlato più volte – fare rete tra professionisti di questo tipo; cioè nella nostra fascia di età, dopo la devastazione che hanno creato le generazioni precedenti a livello accademico e per posizioni di ricerca, in particolare per quanto riguarda la bioetica, che secondo me oggi versa in uno stato molto precario, dove le scuole di formazione e i dottorati, come sai bene anche tu, del (? 11:26) hanno ridotto la portata della formazione, pur partendo da secondo me modelli anche di eccellenza che c’erano nel nostro Paese e raramente altrove, oggi questo lo assistiamo insomma, oggi secondo me ha un grande impoverimento, per cui bisogna fare rete tra questi servizi. E terzo motivo, perché credo che di per sé l’iniziativa sia coraggiosa, cioè non solo bisogna fare rete tra professionisti, ma tra istituzioni di questo tipo, cioè simili al CEC; e questo è uno dei grandi obiettivi, che so che (*redacted*) sta cercando di costruire e che mi vede assolutamente allineato, cioè io sono membro, come revisore dei progetti, della (*redacted*), per cui io rivedo tutti i progetti di etica, quindi faccio le (? 12:09) mission anche a tutti gli altri comitati etici per la sperimentazione clinica; lì vedo l’altro lato, come dire, dal punto di vista della ricerca clinica universitaria e degli istituti di eccellenza in Italia, mi sembra molto chiaro che i comitati per la sperimentazione clinica sono del tutto inadeguati nella misura massima a poter offrire un supporto concreto a professionisti sanitari su questioni di etica clinica. E quindi era anche una modalità per cercare di, come dire, creare e sostenere, vedere se può funzionare un servizio di questo tipo.

```
D:	Grazie. Mi spiegheresti un po’ meglio in cosa consiste il tuo lavoro in questo servizio? Il tuo ruolo in particolare.
R:	Allora, in quello del CEC consiste nell’offrire rispetto alle altre prospettive, almeno io lo descriverei così, un tipo di expertise che è di tipo accademico e che si rifà ai temi della bioetica, dell’etica e della filosofia morale. Quindi un conto è la bioetica, un conto è la filosofia morale; sono due cose che sono secondo me sovrapponibili in larga parte, ma non è detto che una persona che abbia una formazione clinica abbia una formazione sufficientemente approfondita di filosofia morale, cosa che secondo me invece in molti casi è un valore aggiunto. Come tante altre expertise, oggi le identità in bioetica sono multi faceted, come si direbbe in inglese, non mi viene la parola, ma insomma sfaccettate, in italiano. Quindi si può essere bioeticisti o bioeticiste in tanti modi diversi, avendo delle expertise su profili molto diversi. Ecco, siccome il mio profilo include quello dell’etica clinica e della filosofia della scienza e della filosofia morale, il mio ruolo secondo me è aggiungere alle altre expertise che ci sono disponibili il punto di vista di chi ha questo tipo di expertise e poi, che è una cosa separata da questo che è, come dire, oggettivo, offrire il mio punto di vista. Ė una cosa difficile da fare, molto difficile, soprattutto sui problemi delicati che stiamo trattando oggi. Cioè, un conto è offrire gli strumenti di analisi, un conto è avere anche il coraggio, se vogliamo, di esprimere un’opinione che può essere anche contro corrente rispetto a quella degli altri membri. Però io credo che questo sia il valore aggiunto, cioè se non facciamo questo non serve a niente. Quindi sono questi due tipi di contributi che io immagino siano di maggior valore, poi si traducono nelle altre attività, come dicevamo prima, la revisione era didattica, quello che compete queste aree io volentieri, se posso, contribuisco.
D:	Va bene. E invece cosa pensi del valore di un servizio come quello che offre il CEC per quanto riguarda invece il lavoro degli operatori sanitari? Cioè, secondo te dal loro punto di vista qual è il valore aggiunto? Anche qua, in realtà abbiamo già parlato di questo, però se vuoi insomma aggiungere qualcosa su un punto di vista del clinico.
R:	Sì il punto che aggiungerei è questo: cioè secondo me il valore, però questo in parte il CEC io trovo che a parte gli elementi che abbiamo detto prima distintivi condivida con l’unità di bioetica questo valore aggiunto e che è quello di fare vedere agli operatori sanitari che c’è una legittima dimensione di incertezza e di expertise su questioni morali, che riguardano la medicina. E questo è per molti una realizzazione e uno shock, molto spesso; cioè non riescono in molti a comprendere che molte delle cose che fanno le fanno per abitudine, come dicevo prima, e non perché ci hanno pensato bene; e che se ci pensano bene non sanno rispondere loro stessi, malgrado (? 16:03) delle expertise a questioni che in realtà poi vedono in maniera quotidiana. Per cui secondo me il valore veramente aggiunto del CEC e dell’unità di bioetica consiste nel far emergere, o per meglio dire – a me piace anche questa dicitura – nel rendere esplicita questa dimensione, che altrimenti è sottostante alla pratica clinica e nel renderla esplicita si crea l’opportunità di essere più consapevoli. Il CEC non fornisce molto spesso come unità di bioetica, o la bioetica stessa, delle soluzioni pienamente soddisfacenti, ma quello che può fare sempre è aumentare la consapevolezza delle persone coinvolte, capire quali ragioni… e in alcuni casi, secondo me però, questo (? 16:52) è quello che si dice, ma in alcuni casi invece il problema li risolve. Cioè secondo me ci sono delle visioni sbagliate a livello concettuale etico, che un approfondimento in molti casi serve invece a togliere dal tavolo. E quindi secondo me ha questo tipo di valore aggiunto, di lavorare su proprio queste dimensioni. C’è anche una dimensione che faccio sempre fatica e distinguere, ma che per me va distinta in maniera totale, che è appunto quella del (? 17:20), abbiamo visto come negli ultimi casi il CEC è presente anche su questo tipo di expertise, dov’è la mia expertise, quindi, come dire, io mi limito nel mio commento a questo profilo che riguarda appunto più il mio punto di vista, però io credo che il CEC, anche sulle questioni di biodiritto, possa fornire altrettanto un tipo di parere, di consulenza che può essere molto utile, perché molto spesso i due problemi vanno assieme; sono due dimensioni distinte che vanno distinte, però per chi si trova a fare l’operatore sanitario oggi, è giusto conoscere i problemi morali, è giusto conoscere cosa dice anche la legge, il diritto rispetto a quello che si deve o non si deve fare ecco.
D:	Anzi, forse da un certo punto di vista è ancora più importante. Ascolta, invece pensando alle attività che svolge il CEC, in che modo secondo te sono state integrate nel contesto locale?
R:	Questo è difficile da dire, io immagino che siano state integrate e soprattutto grazie alle relazioni personali. Cioè fino a adesso secondo me il check è arrivato laddove sono arrivate le relazioni personali, delle persone che lo compongono, delle persone che lo sostengono, delle persone che l’hanno voluto e delle persone che conoscono persone che l’hanno voluto, ci partecipano e ci lavorano. Quindi secondo me il grado di integrazione per adesso è tutto sommato relativamente basso e limitato a questo tipo di interazione. Che cosa fare per migliorarlo? Forse se hai un’altra domanda su questo, se ce l’hai te lo dico dopo.
D:	Va bene. Allora, ti chiederei… no, la prossima domanda è sui limiti che secondo te ci sono rispetto al CEC; quindi se ci sono dei limiti, che siano organizzativi, che siano interni al CEC, che siano esterni al servizio, che siano economici, secondo te appunto esistono degli ostacoli al lavoro del CEC attualmente?
R:	Allora, secondo me l’ostacolo è proprio nella sua integrazione all’interno della struttura sanitaria. Bisognerebbe trovare un modo di aumentare questo tipo di integrazione; dall’altro lato è una valutazione che lascio anche alla Presidente, cioè il carico di lavoro di unità di bioetica e del CEC non può essere infinito, cioè c’è anche una scala all’interno della quale, qualora il check per esempio dovesse prendere veramente in carico la valutazione delle richieste di morte medicalmente assistita, improvvisamente il carico di lavoro sarebbe molto più alto per il CEC, faremmo credo solo quello o prevalentemente solo quello, vista la delicatezza dei temi e dei casi che si presentano e si presenteranno. Quindi ecco, quindi anche molto dal periodo contingente, secondo me un limite è a parte quello di integrazione è quello legislativo. Cioè al momento non è chiaro che cosa fa il CEC, che cosa dovrebbe fare il CEC per proprio lo Stato Italiano, semplicemente; cioè quali sono i tipi di casi che dovrebbe trattare, qual è (? 20:30) di Statuti e futuro incerto, che rende difficile strutturare una programmazione. Io sono dell’idea che questo limite vada superato costituendo più CEC possibili, quindi sono dell’opinione che il CEC sia non sono utile ma assolutamente necessario e vada creato in ogni struttura sanitaria complessa, di una certa entità e pertanto forse ecco, il limite maggiore è questo, vedendo la vostra esperienza, eravamo già pronti a passare, a processare questo tipo di caso, prepararci con la formazione, poi arrivano dei decreti, delle leggi ministeriali, delle circolari che scombussolano le cose, per cui è difficile capire quale sarà poi il futuro che viene richiesto. Da questo, a cascata, altre cose, perché naturalmente se prendiamo questo compito, poi ci servono, come abbiamo detto, altri membri, con altre expertise, quindi insomma è molto contingente a questo. Ultimo profilo, più venale, è il fatto che la partecipazione è completamente gratuita al CEC. Io non so se questo limita la partecipazione di altre persone o se disincentiva sul lungo periodo, so che c’era stata una valutazione dopo - lo dico per trasparenza - dopo il primo anno; di questo aspetto, cioè il primo anno era sicuramente pro bono, dopodiché si è proseguiti così. Per quanto mi riguarda non è un problema, io per vari motivi avrei addirittura difficoltà a dire la verità, accettare dei soldi da un’altra struttura pubblica, proprio la mia collaborazione con altre strutture, per cui però ecco, questo forse è anche una questione che vale la pena di valutare a intervalli regolari, perché soprattutto se poi l’impegno, scattando questi meccanismi diventasse molto più grande di così, già così sono svariate ore, quindi non tutti, come dire, considerano solo formazione accademica la propria partecipazione; magari, non ho nessuno in particolare in mente, però viste le partecipazioni di altri organismi mi chiedo se qualcuno la pensi così.
D:	Certo, una domanda legittima. Allora, invece ti chiedo se ci sono oltre ai limiti, delle potenzialità che vedi rispetto al CEC, che è stato implementato, e se sì quali. Anche qua ne abbiamo già elencate diverse.
R:	Sì, tantissime di potenzialità, ce ne sono tantissime; dipende appunto da questo futuro incerto, in particolare sulla morte medicalmente assistita. È questo il grande tema, inutile nasconderselo, a seconda di come riusciremo, come il CEC sarà integrato nei processi decisionali, tutto quello che abbiamo detto prima potrebbe cambiare drammaticamente. Cioè, qualora diventasse un punto di riferimento imprescindibile per le strutture di Modena e Reggio Emilia nella valutazione di questi casi, di per sé diventerebbe immediatamente integrato nei processo decisionale, sarebbe molto chiaro a tutti che cosa fa e credo che la voce si spargerebbe molto velocemente, riguardo alla sua esistenza, ai pareri che rilascia e le altre cose. Quindi questa secondo me è una grandissima potenza e potenzialità; un’altra potenzialità è secondo me quella di creare - però questa è un’idea mia wide, che prende spunto da quello che ho visto quando io sono stato alla Medical School di Harvard - e cioè che loro avevano un seminario mensile tra tutti i dipartimenti, nei quali si presentava un caso che era già chiuso nel passato e lo si discuteva assieme, non era una cosa chiusa, ma una cosa aperta, quindi bastava sottomettere il caso e questa cosa nel corso degli anni ha permesso di creare un gruppo costantemente presente all’interno di ospedali dell’area di Cambridge e Boston, di discussioni di questi problemi, molto conosciuto, guidato da persone con un’expertise più in bioetica o comunque nel seguire problemi di questo tipo, cioè che potrebbe avere una funzione di coordinamento e questo ha un po’ fatto da volano, cioè ha… Non so bene come questo possa essere implementato, possa avere una forma declinata all’interno della nostra realtà locale, però ecco, forse al posto che la formazione solo interna, che ha avuto secondo me risultati modesti perché abbiamo fatto quattro lezioni. Però di fatto insomma molti erano, alcuni lo sapevano già cosa facevamo, tanti secondo me malgrado gli avvisi non hanno partecipato, non hanno saputo cosa facevamo, forse questa è un’altra maniera di attirare l’interesse, di far vedere che cosa si fa. Però potrebbe anche essere una cattiva idea, non lo so, però là aveva funzionato.
D:	Okay. Scusami, ti faccio una domanda in più sulla questione del suicidio assistito invece; non ho capito se il tuo è sostanzialmente un augurio, cioè nel senso se dal tuo punto di vista il CEC è la struttura, il CEC o una struttura simile al CEC è diciamo l’operatore ideale per occuparsi di casi di questo genere, quindi ti auguri che a un certo punto arrivi una decisione dall’alto, che tutti i casi di questo genere vengano messi in mano ai CEC, posto che insomma, come dicevi prima, non esistono in tutte le realtà a oggi, o se la vivi, o se sei ambivalente, cioè la vivi anche con preoccupazione, come a dire se arriva questo compito poi ci dovremo occupare solo di questo, non rimarrà spazio per altro. Quindi volevo magari chiederti un approfondimento su questo.
R:	Sì, allora, tutti e due gli atteggiamenti sono più dal primo, cioè sono più da… allora innanzitutto sono molto favorevole, credo che i CEC siano le strutture corrette per farlo, credo che una maniera intelligente di processare questa cosa sia di dire là dove non esistono i comitati per l’etica nella pratica clinica, la funzione viene fino a quando non vengono stabiliti ricoperta dai comitati per la sperimentazione clinica, nella loro lista ridotta come (? 26:48) gli ultimi sviluppi legislativi, ma che là dove ci sono i CEC invece spetterebbe a loro; posto il fatto che in entrambe le istituzioni debbano essere presenti alcune expertise di fondo, senza le quali è impossibile poi di fatto valutare in maniera corretta queste domande. Sul secondo profilo, come dire, se non lo facciamo noi, chi? Cioè è chiaro che si può stabilire una Commissione Tecnica per anche queste cose, lasciando… però forse questo è proprio il caso - nel caso è utile uscire dal tecnicismo - allargare la prospettiva a qualche cosa di più immediata e più interdisciplinare, con più punti di vista, mentre invece fare una Commissione da quattro esperti, che si basa a ratificare i parametri della Corte Costituzionale, secondo me rischia di rendere eccessivamente burocratica la cosa. E tra l’altro, aggiungo a questo, come dimostrano i casi di cui non possiamo parlare in maniera dettagliata, ma di cui discutiamo domani insomma, tu li conoscerai e li avrai sentiti; in molti casi non è che una questione di ratifica di questi profili, cioè molti casi sono spinosi, molto spinosi, proprio perché non si capisce che cosa bisogna fare, qual è la cosa giusta da fare dal punto di vista legislativo, clinico e anche morale. E per cui io credo che vista l’esistenza di questi casi, un organismo come il CEC sa decisamente tra le altre alternative, cioè il Comitato per la Sperimentazione Clinica da una parte e dall’altra il selezionato gruppo di quattro esperti in Commissione Tecnica, la soluzione intermedia migliore.
D:	Okay, grazie.
R:	Se arrivano tanti casi, cercheremo di fare fronte.
D:	Alla richiesta. Allora, ti faccio le ultime domande; allora intanto ti chiedo come valuti in generale le attività di questi sedici mesi, da appunto l’implementazione del CEC.
```

R:	Secondo me il risultato è stato molto positivo, la conduzione della nostra valente Presidente della Segreteria Scientifica è stata ottima, lo dico perché sono coordinatore del (*redacted*), ho grossi problemi a coordinare le cose e quindi so perfettamente che non è per niente facile condurre un gruppo di questo tipo su temi così spinosi. Per cui secondo me è stato fatto un ottimo lavoro fino a adesso, da questo punto di vista. Forse, ecco mi piacerebbe vedere, così, per risultato futuro, abbiamo in progetto alcune pubblicazioni, per esempio mi piacerebbe partecipare; questa è una cosa dal mio punto di vista, mi spiego meglio: cioè, posto il fatto che è un’esigenza per me di formazione non retributiva, quella della partecipazione al CEC, se riuscissimo a sfruttare in maniera accademica il nostro lavoro, rendendolo visibile, credo che sarebbe una cosa molto bella. In parte ci stiamo provando, in parte è anche colpa mia, che dovevo rivedere un articolo, adesso gliel’ho rimandato, ma sono molto in ritardo. Però, detto questo, credo ecco che questo sia un profilo interessante da esplorare sulle nostre attività, la produzione accademica è legata alle attività del CEC.

```
D:	Quindi per esempio pubblicazione di case report o…
R:	Sì, io credo che sia interessante esplorare anche questo lato e credo che ormai c’è un po’ di materiale, per cui si può partire a farlo.
D:	Indubbiamente. E poi invece arriva la domanda sui miglioramenti; cioè, secondo te in che modo il CEC potrebbe essere migliorato?
R:	Una domanda molto difficile per le cose che ho detto prima, cioè distinguerei sempre i due scenari: se rimaniamo nello stesso legislativo, semplicemente facendo girare di più la macchina del check all’interno della struttura di appartenenza, cioè quindi facendo sempre più eventi di… cioè creare occasioni di contatto con i professionisti sanitari, perché il CEC è molto bello, ma è utile solo nella misura in cui riceve richieste.
D:	Certo.
```

R:	Se non riceve troppe richieste non serve a granché. Poi io credo che possa essere utile per le sue caratteristiche; una volta, parlato in passato, non abbiamo mai finalizzato, credo, per creare – ma questa è una visione sempre molto accademica – anche dei gruppi volendo di studio, insieme a delle unità su questioni particolari che sono aperte; abbiamo parlato della rianimazione per esempio dei feti prematuri, dei nascituri e quindi anche una questione molto interessante, con il quale potrebbe valere la pena di costruire delle relazioni con le strutture, proprio con i clinici per approfondire e scambiare del materiale. Quindi secondo me anche questo potrebbe essere una (? 31:52) con maggiore integrazione. E poi per il resto a livello di rete nazionale, credo che bisognerà proseguire, in quanto (*redacted*) ha già iniziato in maniera ammirevole, quindi la costruzione di rapporti tra organismi che già esistono di questo tipo, proprio alla luce degli obiettivi che dicevamo prima.

```
D:	E fare rete come dicevi.
R:	Sì.
D:	Invece prima, giusto per chiarire se ho capito, ti riferivi più alla questione appunto delle policy, delle linee guida.
R:	Sì, ecco, io credo, sì, esatto, io credo… sulla questione delle policy ho  una visione molto ambivalente, cioè non sono molto sicuro che sia utile, francamente. Credo che su alcuni casi sia utile, in alcuni casi specifici, (? 32:41), ma la produzione di documenti di questo tipo - ne abbiamo lasciati in sospeso credo due, per esempio – richiede una quantità di tempo incredibile, per essere… Difficilmente vengono letti e rispettati, difficilmente (? 32:56), secondo me, devo essere sincero, la scelta che è stata compiuta negli ultimi mesi, volenti o nolenti o per fattori esterni, oppure deliberatamente, di lasciare perdere questo fattore, che era quello più presenta all’inizio, quindi la struttura di documenti di linee guida e di indirizzi comuni, per sforzarsi un po’ di più o alla formazione o al trattamento dei casi, credo che sia la mossa vincente, proprio perché il primo di solito ci sono… è pieno di Commissioni, di organismi, di società scientifiche, che rilasciano una quantità di documenti tra loro inconsistenti incredibile; hanno problemi su tutti i profili questi documenti, quindi a meno di non fare su richiesta esplicita una policy o uno statement, una dichiarazione su una questione molto ben definita, sulla quale sappiamo che possiamo dare una visione così, sono scettico che sia veramente un compito utile, in molti casi.
D:	Okay. Grazie per il tuo punto di vista. Allora, le domande sono finite, rispetto a quello che è emerso hai altri pensieri, qualche ulteriore commento da condividere? Secondo te ci sono elementi importanti che non sono venuti fuori?
R:	Ci penso un secondo. No, credo che ci siamo veramente detti tutto.
D:	Allora, direi di concludere l’intervista, ti ringrazio per la disponibilità e ti chiedo anche eventualmente di darmi disponibilità se puoi, se rileggendo le trascrizioni dell’intervista mi accorgo che ci sono magari dei punti da approfondire.
R:	Naturalmente sì.
D:	Va bene, allora ti ringrazio. Allora chiudo la registrazione.
```
