# Supplementary material for: How to implement a clinical ethics committee in an oncological research hospital: Qualitative results from a process evaluation study using normalization process theory (EVACEC)
Source: PLoS One. 2025 May 6;20(5):e0318870. doi: 10.1371/journal.pone.0318870 (PMC12054913; doi:10.1371/journal.pone.0318870)
Supplement: S5 File — Interviews transcripts_raw data: this folder contains all the raw data used in this study, the interview transcripts (in original Italian). (ZIP) [file pone.0318870.s005.zip › S5_Interviews transcripts_raw data/09CM.doc]

```
D: 	Eccoci qua. Dunque, le stavo spiegando un po’ il progetto dello studio, se è tutto chiaro procedo con le domande.
R:	Sì, è chiarissimo, pensavo di aver perso qualche pezzo, no.
D:	Invece no, è tutto chiaro, era già tutto chiaro. Benissimo, allora come prima domanda le chiedo di raccontarmi la sua esperienza diciamo con l’etica, la bioetica nella sua attività professionale.
R:	Dunque, senza specifico riferimento al CEC?
D:	Sì, ci interessa in generale il suo background e poi le farò domande ovviamente anche sul suo lavoro nel CEC.
R:	Io sono un pediatra e mi occupo - come vede scritto qua - di cure palliative pediatriche; sono il responsabile di questo Centro Regionale di riferimento. Io ho conosciuto la bioetica a Reggio, perché lavoravo… ero il primario della pediatria a Reggio quando (redacted)arrivò e quindi ho un pregiudizio, ho un bias, nel senso che a me la bioetica me l’ha introdotta (redacted), per quello che non posso dire che tutta la bioetica si riassume nella (redacted), però insomma le porte me l’ha aperte lei. Dopodiché mi sono trovato a partire da… (redacted) è arrivata nel 2016, abbiamo collaborato fin da subito, io ho cominciato a collaborare con questo Centro nel (redacted), dal (redacted)sono a tempo pieno e devo dire che negli ultimi due anni e mezzo abbondanti praticamente la bioetica mi accompagna tutti i giorni perché trattandosi di pediatria, di soggetti in crescita con… diciamo se posso chiamarli dei “lungo sopravviventi” di malattie gravi, croniche, con scelte dei caregiver costanti, la bioetica è diventata una sorta di compagno quotidiano. Per cui quando l’anno scorso - giusto? Cos’è, da un anno e mezzo che lavoriamo al CEC? - (redacted) mi ha coinvolto l’ho sentita come una grande opportunità, anche se anche ora mi sento spesso, come posso dire, non fuori luogo perché ho imparato tantissimo, ma insomma come pediatra mi rendo conto che abbiamo una casistica molto diversa rispetto a quella dell’adulto, che è molto particolare l’approccio, ma non semplicemente perché sono bambini che, come dire, ti spezzano il cuore, ma perché proprio sono diverse le situazioni. La bioetica, nel senso proprio di non fare danno, di non dare maleficenze insomma, di pensare alla qualità di vita, di coinvolgere i genitori, nel mio caso è talmente costante che mi sento spesso diverso, in una diversa posizione rispetto ai medici dell’adulto, che curano gli adulti che insomma hanno a che fare con soggetti totalmente responsabili della loro azione. Non so se è un privilegio quella, comunque è una caratteristica che io non ho mai… per cui è un po’ così, è una piccola ambivalenza.
D:	Quindi dice che, nel suo caso, per il tipo di lavoro che svolge, la problematicità diciamo alla base di tutte le altre problematiche che poi incontra è proprio il fatto che il bambino di per sé è un soggetto che non… chiaramente può dare il consenso fino a un certo punto, per cui decide qualcun altro comunque.
R:	Sì, non è titolare, non è responsabile di tutto e per di più, siccome mi occupo… mentre quando ero in pediatria a Reggio mi occupavo di tutti i casi e adesso ci occupiamo di casi, come è logico che sia, i più gravi, moltissimi di questi casi sono, come posso dire, dei fine-vita fin dalla nascita. E quindi la bioetica, nel senso soprattutto di analisi dei valori dei genitori e dell’effettiva ricaduta di questo e del facilitare diciamo la ricaduta della volontà e dei valori dei genitori, è per quello che dico che è quasi una costante insomma. La pianificazione condivisa delle cure è diventata il cuore centrale del nostro lavoro e, come (redacted) sa insomma, la bioetica la sentiamo come l’esigenza di avere il pane quotidiano. Quando fino a poco tempo fa pensavo fosse filosofia, onestamente.
D:	Sì chiaro, poi quando uno invece comincia a prendere un po’ la mano si accorge di tutte le problematiche che invece sono molto concrete, nella pratica clinica. Ascolti, mi parla un po’ invece della sua esperienza con il CEC?
R:	Dunque, come ho detto all’inizio mi sono avvicinato a questa esperienza con anche un po’ di timori perché ero convinto che dovevo riuscire a dare un apporto fattivo insomma, di essere più spettatore che partecipe; in realtà, per come è stato poi strutturato, adesso non so questa ricerca cosa porterà, però sulla mia pelle ho proprio assistito alla crescita di una creatura. Quando ho iniziato le prime riunioni ero davvero convinto che come pediatra sarei potuto intervenire poche volte su casi specifici, che non ce l’avrei fatta ad avere un parere, chiamiamolo, originale su molti casi perché pensavo fossero troppo lontani dalla mia competenza, cultura o sensibilità. Invece mi sono accorto che per come il lavoro è stato organizzato, per come il gruppo si è formato, per come si è relazionato generalmente parlando con la trasparenza e una forza, come posso dire, di volontà notevole, poi mi sono trovato un po’ nel gruppo senza sentirmi a latere, senza sentirmi lontano. Ha aiutato anche un po’ il fatto che il primo caso presentato era pediatrico, per cui mi sono potuto spendere con delle competenze personali e poi ho capito insomma che si trattava di un metodo e non di una competenza specifica, di un approccio diciamo.
D:	Certo. Ascolti, forse mi ha già risposto alla prossima domanda, ma in caso le chiedo di aggiungere qualche commento; volevo chiederle che cosa l’ha spinta in particolare a dire sì a (redacted) e ad accettare insomma di diventare membro del CEC.
R:	L’anno scorso, quando mi è stata fatta… chiaramente un po’ prima dell’inizio dei lavori, dunque sì, io ero proprio agli inizi del mio lavoro a tempo pieno e stavamo creando un’equipe neonata proprio e ho immediatamente pensato, un po’ istintivamente, che mi sarebbe servito molto come crescita personale e così è stato. Devo anche dire, prima parlavo di bias non a caso, che non me la sarei mai sentita di dire di no a (redacted) che mi aveva… mi considero quasi, cioè a tutti gli effetti sono un allievo suo, perché sono nato, non sapevo nulla prima del suo ingresso nel mio reparto vecchio di pediatria, per cui gli ho chiesto se lei pensava che io fossi utile e non mi sono neanche posto il problema francamente. In seconda battuta, subito dopo ho pensato che – e non mi sbagliavo – che sarebbe stata per me una grande opportunità formativa, avevo pensato sono troppo vecchio probabilmente per farlo, di iscrivermi a un master di bioetica, quello di Torino, poi non lo so se sbaglio ma insomma mi sembra che questa esperienza sia abbastanza arricchente, anzi decisamente.
D:	Grazie per il suo riscontro. Adesso le faccio invece qualche domanda sul CEC in sé e per sé, quindi esuliamo anche dal suo ruolo. Secondo lei, qual è il ruolo del CEC all’interno dell’ospedale, dell’azienda USL di Reggio?
R:	Adesso cerco di rispondere per quello che appunto è la mia visuale, che come le ho detto…
D:	Sì, certo, questo è quello che ci interessa, la sua opinione.
R:	Io penso che fondamentalmente, per usare delle parole un po’ troppo semplici diciamo, il cosiddetto “plain language” dovrebbe aiutare i professionisti nelle scelte difficili, nelle scelte anche molto difficili diciamo, perché tante scelte in campo medico sono sicuramente difficili, ma quelle molto difficili, che riguardano soprattutto la sofferenza, il rispettare il volere dei pazienti, dei caregiver, dovrebbe essere un po’ al fianco dei clinici nei momenti difficili. Quello che abbiamo registrato, soprattutto direi nell’ultimo caso che è stato presentato, ma anche in altri, che siamo arrivati tardi; cioè siamo arrivati quando le cose si erano già svolte e siamo non dico che siamo serviti a leccare le ferite diciamo, ma un pochino sì, a lenire le ferite, di percorsi molto difficili, quando invece probabilmente il CEC dovrebbe essere pensato come una cassetta degli attrezzi diciamo molto tempestiva. Quando abbiamo iniziato, a parole e anche scritte, se ricordo bene, c’era questo concetto, che dovevamo – ed era anche un po’ la cosa che mi preoccupava – che dovevamo essere disponibili a dare delle risposte anche in urgenza. E penso che sia un po’ il pezzo più difficile, ma anche più gratificante di questo Comitato, che dovrebbe integrarsi con le… diciamo riuscire a intercettare i filoni, le aree o i gruppi di persone più esposti a rischi diciamo ambientali, legati alla bioetica, per poterli affrontare assieme a loro, mentre succedono.
D:	Certo. Sì, chiaramente questo non è detto che sia sempre possibile, perché i tempi della clinica purtroppo sono molto veloci.
R:	Esatto. In qualche caso presentato ci è andata vicina questa cosa; nel primo pediatrico, che tra l’altro conoscevo bene il deceduto, mentre noi chiedevamo un secondo round di pareri, quindi in effetti è successo così. Però devo dire che mi ha stupito, di nuovo appunto io ho questa compromissione personale, perché fino a poco tempo fa lavoravo lì, conoscevo tutti, adesso invece sono un componente esterno, però chiaramente conosco ancora un sacco di persone e mi ha stupito. Tantissime persone hanno partecipato alle formazioni che abbiamo fatto, io ero anche relatore tra l’altro, e quindi è un dato positivo. Però dall’altra parte c’è una marea di persone che non sapevano che esiste, che non sanno che esiste quest’opportunità e sono operatori a mio avviso molto esposti a questo, che rischiano di affrontare da soli delle decisioni piuttosto laceranti.
D:	Quando dice così ha in mente reparti in particolare, cioè per esempio lei pensa alla pediatria o pensa anche ad altre strutture?
R:	No, sicuramente non è un giudizio il mio.
D:	No, chiaro, è una sua visione, no, certo, è giusto per…
R:	Non è una pagella.
D:	No, certo.
R:	La rianimazione, dove finiscono anche i bambini, dove recentemente ho avuto occasione di contribuire a gestire un decesso, ha pochissimo, cioè non ha intercettato il CEC come enorme opportunità (? 14:00) e penso, per quello che capisco io, per la visione, non so se sbaglio, nel bene e nel male la rianimazione è la quintessenza delle nostre tecnologie più sofisticate e, come posso dire, un po’ ipocritamente dimentichiamo che lì pazienti anche molto complicati possono essere mantenuti in vita a lungo contro leggi di buon senso, dal mio punto di vista, e comunque per dire che c’è il tempo, non è vero che non c’è il tempo, non è vero che bisogna decidere tutto in un quarto d’ora; per l’amor di Dio, esistono quelle situazioni. Ma in realtà io penso agli ambienti intensivi, ho visto la neonatologia, la terapia intensiva neonatale molto più coinvolta, anche perché ci sono… il direttore è all’interno del CEC e la rianimazione dell’adulto un pochino più scoperta e per quello che non conosco io bene, perché appunto la medicina dell’adulto, secondo me le medicine, le medicine interne, che hanno quantità industriali di grandi vecchi, di demenze, di malattie non oncologiche sono molto poco esposte a questo. Mentre la parte, come posso dire, oncologica, hospice, il reparto di cure palliative all’interno dell’ospedale mi sembra che stia facendo un ottimo lavoro. Però ci sono zone dell’ospedale sicuramente… non è che sono… non è che non reagiscono o non aderiscono, proprio manca nel loro menu quella voce qua, nello spartito non ci sono queste note, così come sul territorio è molto verosimile che le cure primarie, soprattutto i medici di medicina generale, molti pediatri di libera scelta non conoscono questo, sul territorio trovo un pochino più, anzi decisamente più attenta all’argomento le infermiere dell’assistenza domiciliare, i servizi domiciliari. Però insomma penso che questi siano gli argomenti.
D:	Va bene, grazie. Torniamo un attimino al lavoro del CEC; le volevo chiedere quale… in che modo secondo lei il servizio che offre il CEC è diverso rispetto ad altri servizi promossi in ospedale e qual è la sua specificità.
R:	Ė totale, cioè non c’è niente di paragonabile. Non abbiamo affrontato una marea di casi, ma quello che ho imparato da (redacted) e altri esperti, siamo abbastanza in linea con quello che capita a CEC presenti da tanto tempo in altri luoghi. Il tipo di argomenti, anche le modalità di risposta, la collegialità estrema, perché noi siamo in quindici, mettendo anche solo metà di noi, perché oggettivamente è andata quasi sempre così, (? 17:44) un contributo, componenti interni, componenti esterni; cioè il metodo è molto… innovativo è una parola un po’ stucchevole diciamo ecco, sicuramente nuovo, cioè penso che parte del problema e delle motivazioni dei discorsi che facevo prima, cioè del fatto che molti non lo conoscono sia anche questo. Che molto nuovo non è un servizio di consulenza di uno specialista d’organo, ovviamente, non è il servizio di consulenza che neanche in un PDTA allargato può dare, è qualcosa di molto diverso che può uscire dall’ospedale o dalla sanità, perché questa è l’altra caratteristica, penso che sia uno sguardo, come è opportuno che sia, rivolto anche al di fuori degli aspetti sanitari e questa forse in assoluto è la specificità più grande, perché un organo istituzionale di un’azienda sanitaria locale solitamente si limita agli aspetti medico-sanitari e con il CEC ci sono opportunità sicuramente di spingersi verso gli aspetti spirituali, religiosi, sociali, relazionali, culturali, che è un po’… cioè io mi ci sono ritrovato molto perché è un po’ la materia prima delle cure palliative.
D:	Sì, certo, di guardare oltre le specificità della patologia, se posso rielaborare con parole mie.
R:	Sì, è così; cioè a noi non interesserà mai, suppongo che non sia neanche lecito mettere in discussione il parere dello specialista d’organo o di funzione o l’indicazione a fare cose, a procedure, a terapie di quello specialista. Il perché e il se farle può diventare oggetto di dibattito etico.
D:	Certo, chiarissimo. Anche se anche qua, alla prossima domanda forse mi ha già risposto, ma gliela faccio ugualmente; l’obiettivo generale del CEC secondo lei qual è e in che modo agisce nel contesto locale?
R:	Penso che dovrebbe essere, anche qua tendo a confondere il mestiere che faccio tutti i giorni con quello; cioè dovrebbe essere quello di introdurre in un modo se vuole anche un po’ pedagogico l’approccio etico nelle scelte mediche. Cioè non decidere che si fa una cosa perché si può fare, ma decidere che si fa una cosa non solo perché si può fare, che ovviamente è la prima domanda, ma perché ha un’assoluta utilità e non dà nessun danno, perché quel paziente è d’accordo di farla, perché ha un senso farla, perché… cioè, bisogna dare un perché, non solo perché… il perché non può essere solo tecnico.
D:	Sì, su questo punto sono totalmente d’accordo con lei. Ascolti, parliamo invece un po’ di lei, anche se anche qua già mi ha detto molto, però le chiederei magari di esplicitarlo; il suo ruolo specifico, il suo lavoro nel CEC, ha voglia di parlarmene un po’?
R:	Il mio contributo (? 21:33)?
D:	Sì, esattamente. Cioè in cosa consiste il suo lavoro all’interno del servizio.
R:	Come ho detto prima io sono un pediatra, ho sempre fatto il pediatra, al di là che ho anche una specializzazione in gastroenterologia, insomma un pochino ho lavorato anche con gli adulti, e poi faccio il palliativista. Quindi da un lato ho alcune competenze, la pediatria è una delle ultime branche della medicina non iper specialistica e quindi diciamo che ho delle competenze anche tecniche, ma di comprensione di quella fascia di età caratterizzata dalla crescita, dall’evoluzione, ed è sempre tutto relativo, perché i bambini malati crescono poco, ma crescono. Quindi quelle sono competenze utili che posso - ed è successo oggettivamente - mettere in palio, mettere in ballo coi colleghi; però devo dire che la parte che mi sta più arricchendo è che vedo che l’esperienza che mi sto facendo nelle cure palliative, lasciamo perdere che sono pediatriche, ma nelle cure palliative come metodo nell’approccio soprattutto ai caregiver, perché io ho ripeto principalmente bambini con disabilità gravissima, quindi ho il caregiver come interfaccia e quel tipo di dinamica, quel tipo di, come posso dire, quella certezza che ormai ho raccolto, che fa sì che l’analisi dei bisogni la fanno il paziente e il caregiver, la vera analisi dei bisogni la fanno loro, non la facciamo noi operatori, ed è per quello che va rispettata, non è una posizione ideologica. Sono fermamente convinto che l’analisi dei bisogni, cioè cos’è più importante per quel paziente o caregiver, la fa il caregiver; non la può fare il professionista, anche se è il super specialista di quella malattia o è il medico o l’infermiere o l’operatore che pensa di aver capito. Per cui il mio ruolo è stato spesso quello, perché di sicuro sono consapevole, certo che leggo la letteratura, mi informo, ma non… sarebbe abbastanza sciocco insomma che elevassi il mio livello di competenza sulla bioetica come conoscenza di ciò che è pubblicato a quello di (redacted); cioè, il mio compito non è quello di una super esperta in bioetica, di portare anche gli ultimi articoli, letterature o quello del medico legale, che mi dice dove l’etica non confligge ma entra nella legislazione. Io penso che il mio ruolo sia quello di portare la mia esperienza della clinica quotidiana, che è quella di sono un testimone dell’enorme fatica che fanno molti caregiver, nel mio caso, apparentemente una fatica ai miei occhi spesso apparentemente insopportabile, che però con chiarezza porta all’identificazione dei bisogni; se li seguiamo, i caregiver di solito ci danno la strada.
D:	Ascolti, le faccio una domanda di approfondimento su questo, quasi più per interesse mio che a scopo dell’intervista; nella comunicazione con il caregiver per appunto capire quali sono i bisogni e i desideri del paziente, quindi come veicolati attraverso il caregiver, ci sono sempre… diciamo così, la comunicazione riesce sempre e funziona, per sua esperienza, perché giustamente lei diceva l’analisi dei bisogni la fa il caregiver o la fa il paziente, se è in grado. È facile riuscire, cioè facile è l’aggettivo sbagliato, ma si riesce a cogliere, come clinico, quest’analisi dei bisogni? Cioè il risultato dell’analisi dei bisogni? Non so se è chiara la domanda.
R:	La domanda è chiarissima; non è facile, questa è la prima risposta, non è lineare, è un percorso, non è un’anamnesi in cui uno chiede quand’è l’ultima volta che hai avuto mal di pancia.
D:	No, certo.
R:	Ė un - adesso ho semplificato un po’ troppo - è un percorso a cui tutti i professionisti si devono prestare e quello che succede, sempre della seria che decidono i caregiver, è che sono loro a decidere anche con chi aprirsi. Se si lavora in modo interdisciplinare, cioè con una forte sovrapposizione, è un eufemismo, con una forte compenetrazione dei saperi e dei ruoli, sono i caregiver che identificano, quello invece sì. La comunicazione in genere è la cosa più difficile della cura, è la cosa in assoluto più difficile della medicina e della chirurgia anche e della cura, primo; diventa un pochino, non tanto, un pochino più facile o comunque quelle sono le regole che io ho scoperto, le regole di natura, se uno comprende, accetta soprattutto e non si crea dei problemi che i caregiver decidono a chi aprirsi. Non è inusuale che decidano di dire la cosa più importante dal punto di vista comunicativo con l’OSS, con l’ultima in catena gerarchica delle operatrici, purché il gruppo sappia incorporare le orecchie, soprattutto anche la lingua di questa OSS nel proprio… se non si fa così diventa o difficile o addirittura impossibile. I caregiver e i pazienti, con i pazienti che comunicano è un pochino più facile, anzi decisamente più facile; i bambini che comunicano, è banale a dire i bambini non raccontano mai bugie, quindi dicono solo delle verità, quelli che comunicano te lo spiaccicano in faccia, i loro genitori no. Quindi insomma io ho imparato questo, però ripeto, diventa fattibile, percorribile se un gruppo si muove unito, compatto, mostrando abbiamo tutti nei nostri team tante radici che sono diverse, specializzazioni, ruoli; se accettiamo di unirci in un tronco unico in cui poi ci sono dei frutti, la famiglia o il caregiver prende il frutto che trova più aperto. Di solito no, io sono medico ma so, sono consapevole che passo meno tempo (? 29:01) i genitori, quindi tipicamente più spesso sono gli infermieri, chi sta lì, anche per obbligo di servizio, ha più tempo a disposizione perché per parlare ci vuole tempo.
D:	Certo, sì, questo si capisce. Sì, si capisce. Grazie per questa risposta. Ascolti, allora a questo punto le faccio un po’ di altre domande sul CEC, torniamo un po’ all’attività del CEC. Visto che lei è medico, le chiedo, che cosa ne pensa del valore di un servizio come quello che offre il CEC, per il lavoro appunto dei clinici, degli operatori sanitari in generale?
R:	Come ho detto prima, do una risposta complementare a quello che ho detto prima; molti non lo conoscono, altri non l’hanno compreso, altri ne sottovalutano la portata, altri la fraintendono perché c’è anche una categoria piuttosto difficile da gestire di colleghi che sono convinti che consegnano al CEC un problema e che il CEC lo deve risolvere, quando tutti, perlomeno il buon senso sa che non è così, il Comitato Etico non si può né vorrà mai sostituire la responsabilità dei singoli professionisti, è un supporto, è una guida, un’indicazione. Per cui è quello in realtà il nucleo centrale, cioè far capire alle persone che hanno… lasciamo stare che è l’unico in Regione e che sono abbastanza rari in giro per la nazione come situazione, quindi insomma secondo me è un piccolo gioiello che bisognerebbe saper valorizzare meglio, ma nel modo più giusto. Molti colleghi, io lo considero un privilegio, quello di dire con molta tranquillità e serenità, per ora non mi è mai capitato situazioni così un po’ burrascose di spiegare ai colleghi guarda che non è che se hai un problema gigantesco che ti rode l’anima a te, al caregiver, chiami il CEC ed entro domani tutto è risolto, loro sanno cosa dirti, ti danno uno spartito e… guarda che ti aiuteranno, ma la decisione è tua o curante, dell’equipe curante. Questo ruolo bi-direzionale a lungo andare spero che diventi la forza trainante del setting, adesso chiaramente non è che è un ostacolo, è una fatica un pochino da superare, come tutte le realtà nuove che entrano in una medicina che ha ancora purtroppo, inutile negarlo completamente, ancora molto paternalistica insomma.
D:	Certo. Quindi dice in realtà a oggi il CEC viene percepito in modo comunque molto variabile, a seconda del clinico.
R:	Sì, penso che non è colpa dei clinici, cioè che siamo agli inizi e qualcuno ne nega l’utilità senza dirlo, qualcun altro appunto affida a dei poteri taumaturgici impossibili da concepire.
D:	La bacchetta magica.
R:	Sì, e nel mezzo ci stanno le scale di grigio tra i perplessi e gli entusiasti insomma.
D:	Certo. Ascolti, anche sulla prossima domanda mi ha già detto molto, ma le chiedo se vuole aggiungere qualcos’altro sull’integrazione del CEC nel contesto locale. In realtà mi ha già risposto.
R:	Le ho detto tutto. Il CEC si è inserito, è arrivato qualche anno dopo, c’è stata anche la fusione aziendale, anche nella nostra azienda, con grandi trasformazioni anche molto recenti; io nasco ospedaliero e forse tradisco diciamo questa mia provenienza, è chiaro che si è inserito appunto in una realtà in grande trasformazione, con dei piccoli o grandi trambusti, con dei grandi cambiamenti, ci sono molti settori, se posso permettermi, dell’azienda stressati, non solo da ciò che affrontano ma dai cambiamenti organizzativi quindi. Potrebbero vivere, non essersi accorti anche del CEC per quello, o viverlo in modo un po’ anomalo; l’azienda ha dato secondo me un endorsement importante al Comitato Etico, non posso assolutamente lamentare una mancanza di attenzione, è evidente che tutte le cose nuove, come una pianta appena nata, vanno coltivate, vanno nutrite, altrimenti se ne perde o la memoria o il concetto di utilità.
D:	Sì, certo, comunque è un servizio nuovo, quindi è ragionevole pensare che la sua integrazione insomma richieda ancora qualche sforzo. Parliamo invece di limiti del CEC; lei ha percepito rispetto ai servizi o all’attività del CEC qualche limite? Che sia limite organizzativo, limiti interni, limiti esterni al servizio? Anche limiti economici per esempio, so che a oggi il CEC non prevede un gettone per i membri, per esempio.
R:	Sì, non avrei mai anteposto questo; devo dire che questo chiaramente è la cosa più facile da commentare, io stesso sono stato interpellato con questa premessa e l’intervista che stiamo facendo è parte integrante del carattere sperimentale del CEC. A regime, come posso dire, è un mestiere, non è un hobby, per questo che dico mi sembrava filosofia, adesso ho capito che non lo è; cioè non è una cosa teorica, che uno fa a tempo perso, deve diventare, quando sarà spero nel tempo un pochino più diffuso, e tra l’altro prima ho citato spesso gli operatori, ma ci sono anche i cittadini, nel senso che l’attività di bioetica, non dico del CEC, ha dato degli esiti importantissimi sullo sportello o per le dichiarazioni anticipate, voglio dire ce ne sono. A mio avviso il lavoro dell’unità di bioetica di Reggio, io abito a Reggio, quindi sono anche un cittadino reggiano, ho la famiglia qua, ha avuto degli effetti anche questi molto poco conosciuti, ma molto civili, molto civilizzanti. Quindi a lungo andare, nel senso che auguro al CEC lunga vita, deve diventare un mestiere; un po’ l’unico difetto è quello, cioè in questo momento siamo… non è che siamo una banda di scalmanati, però insomma siamo un po’… siamo a tutti gli effetti dei volontari, con tutti gli annessi e connessi, cioè abbiamo dei pregiudizi spero positivi, ma partecipiamo tutti con un background; chi partecipa più assiduamente porta all’interno del CEC delle convinzioni personali che insomma bisognerà un pochino, ripeto, è sperimentale, ci saranno dei risultati che spero diano esito a non dico a regole rigide, ma insomma a delle regole di ingaggio, di reclutamento, di funzionamento. Perché ad esempio è chiaro che senza nessun tipo di compenso o di remunerazione, anche minima, la tempestività di lavoro è un po’ difficile da… la reattività tempestiva è difficile da immaginare.
D:	Questo si capisce. Le vengono in mente altri limiti rispetto alle attività del CEC?
R:	Uno l’ho detto più volte, cioè è veramente poco conosciuto. Cioè il limite è quello, è una materia molto delicata, perché la sanità è una roba strana, cioè se domani c’è un qualcuno di noi, un medico chirurgo che fa la pubblicità alla terapia medico chirurgica più innovativa nata due ore prima, non c’è nessun problema a sbandierarlo ovunque, anche se questa terapia ha delle aspettative, dei risultati molto bassi. Quando invece si tratta di parlare appunto di argomenti che sono difficili, sono pesanti tra virgolette, potenzialmente portano un po’ di tristezza con sé, perché parliamo appunto di scelte difficili, si fa fatica tra virgolette e fare pubblicità, è difficile da diffondere.
D:	Certo. Invece rispetto alle potenzialità, cioè cosa vede, diciamo così, che seme vede potenzialmente fruttuoso all’interno del servizio del CEC, dove lei vedrebbe una possibilità di crescita del CEC?
R:	Sicuramente, adesso andando per stadi, per contrappasso a quello che ho detto prima, un’enorme potenzialità in quegli ambienti appunto come la rianimazione, le terapie semi-intensive; adesso c’è stato anche il tema del Covid, che non è detto che ci abbandoni. Quindi ci sono dei… adesso io mi occupo di bambini, ma sono consapevole insomma, ho toccato con mano che i due estremi, i pochi per fortuna, ma gravissimi bambini molto malati e i tantissimi grandi vecchi malati sono un bacino di utenza enorme. Se l’etica riguarda - perché riguarda quello - la qualità di vita, insomma penso che bisognerebbe portare… devo dire che qua confessare che rischio, anzi mentre parlo confondo le due cose e spesso lo devo ricordare a me stesso, che sono una cosa il servizio di bioetica o l’attività dell’unità di bioetica e il Comitato di Etica Clinica; che sono due attività che si toccano, si potenziano l’un l’altra, per cui io come futuro vedo la crescita di entrambe, non penso che possa, non sarebbe mai nato il Comitato di Etica Clinica senza l’unità di bioetica. Io penso che l’attività di bioetica clinica trascini, trascinerà il CEC, non a caso le consulenze che (redacted) e le sue colleghe fanno nei reparti hanno portato a quesiti del Comitato di Etica. Sono due cose molto diverse, me ne rendo conto, che a volte manco si intersecano, però una spinge l’altra, va tutto nella direzione bioetica verso il Comitato.
D:	Cioè sono complementari, secondo lei, in qualche modo?
R:	Sono complementari e un buon servizio di bioetica, sempre più diffuso, sempre più a disposizione, perché è questa poi la caratteristica del servizio che c’è a Reggio, non è la bioetica lontana e filosofica, è una bioetica praticamente al letto del paziente, a fianco del letto del paziente, a fianco del professionista, il futuro secondo me è quello.
D:	Va bene, grazie. Parliamo invece adesso… concludiamo con le ultime domande sulla sua valutazione del servizio; alla luce di quanto fatto in questi mesi di attività, come valuta lei il servizio implementato?
R:	Adesso darsi una pagella è abbastanza difficile, però come autovalutazione… cioè un po’ autoreferenziale, me ne rendo conto.
D:	No, ma non le farei questa domanda se non mi interessasse.
R:	No, rischio sempre di confondere, come ho detto prima, la mia esperienza col giudizio. La presenza nel gruppo di una giurista, la (redacted), molto brava, cioè, voglio dire, la madre della Legge 219, bioeticista molto preparata; cioè un nucleo coltissimo ha fatto sì che secondo me i lavori, non è ripeto una lista immensa, ma i commenti, le risposte prodotte siano state di buon livello; adesso non so dire se ottimo, eccezionale, sicuramente buono e come ho detto prima può essere, deve essere migliorato sulla tempestività, però questo dipende anche dal momento della segnalazione. Cioè in estrema sintesi, con le forze esistenti che sono state, non direi mai raccattate su, ma insomma si è costruito qualcosa con quello che si aveva a disposizione e che si trovava nell’ambito di una non… non c’era tradizione, non c’era storia alle spalle, quindi un foglio bianco da scrivere, secondo me si è fatto un buon lavoro.
D:	Oltre alla tempestività, le vengono in mente altri aspetti, altri profili sotto cui il CEC potrebbe essere migliorato o insomma lavorare per una crescita?
R:	Non lo so, è chiaro che abbiamo davvero affrontato pochi casi; dalla mia esperienza nelle cure palliative, una cosa che spesso sento come mio limite e che mi potrebbe portare a consultare un CEC, nonostante quello che qualcuno si ostina a negare, è questa multiculturalità, unicità in cui viviamo e che, come dire, non siamo preparati ad affrontare, soprattutto quando ci vengono posti temi etici che riguardano la spiritualità, noi bene o male, fatto salvo, ed è uno… ecco, se dovevo prima esprimere un giudizio positivo di cui mi sono dimenticato, che questo CEC secondo me è un CEC laico. Per laico intendo non antireligioso è, per me laico è un Comitato che ha come faro le regole dello Stato in cui lavora, questa per me è la laicità; mi è venuto in mente perché questo è un lato positivo e dall’altro lato devo però dire che io personalmente, mi è già successo con qualche caso, non sono in grado di entrare, cioè ho… la spiritualità che ci appartiene è quasi sempre quella dominante, quindi cattolico-cristiana; nel mio mestiere io vedo il 50% secco, ma proprio almeno il 50% di pazienti da Paesi dove si va dall’animismo al buddismo, all’induismo o l’islam non se ne parla neanche. Noi non siamo preparati per quello, i Comitati Etici, anche devo dire qua che ho avuto il privilegio di fare un pezzo di lavoro con (redacted), quelli già esistenti sono inevitabilmente - non è colpa di nessuno - legati a doppia mandata con un’unica spiritualità, che è quella appunto cattolica, mentre invece moltissimi dei nostri pazienti e dei quesiti che riceveremo non vengono da famiglie, pazienti o caregiver di quel settore. Quindi lì bisogna fare tanto, non è che la butto lì, l’ho pensato varie volte, secondo me su quel pezzo i comitati dovrebbero avere una componente variabile in cui ad esempio il consigliere spirituale chiamiamolo, il consulente, scusa.
D:	Sì, anche assistente spirituale.
R:	Il consulente spirituale dovresti chiamarlo in modo appropriato alla spiritualità del paziente che incontri; e di nuovo te lo dice il paziente, perché è chiaro che posso avere un paziente dello Sri Lanka che è sikh, buddista, induista o… cioè sono mondi che io non conosco.
D:	No, certo.
R:	Quello è un tema molto importante, che in questo momento francamente non sarei neanche come affrontare in modo pratico.
D:	Comunque se se ne parla è perché è un bisogno che emerge evidentemente, è la cura, la richiesta di assistenza spirituale. Va bene. Invece, fra le attività che svolgete, quindi a partire appunto dalla valutazione, al supporto nei casi complessi, quindi l’espressione di pareri o so che invece all’inizio della vostra attività vi siete dedicati molto alla stesura dei documenti; quale attività secondo lei ha il maggiore impatto o viene percepita come più utile, fra virgolette, dagli operatori?
R:	Noi abbiamo fatto essenzialmente due tipi di attività, abbiamo prodotto dei documenti di consenso, prevalentemente legati alla pandemia, quindi mi sento di dire che sono entrambi utili, perché in quella situazione c’era una massa di persone caratterizzata da problematiche simili ed era molto importante analizzare un tema comune. L’analisi dei casi clinici più complicati, che sono uno, la stessa patologia con gli stessi argomenti clinici, in questo paziente o famiglia è una cosa, nell’altro un’altra; e forse è il pezzo più pregiato del Comitato Etico, però secondo me il Comitato Etico deve fare anche formazione, deve assolutamente fare informazione, altrimenti tutti i punti che ho detto prima, la mancata conoscenza, il disuso, l’uso non funzionerebbero; deve fare diffusione di questi temi e sensibilizzare la popolazione e deve anche, secondo me questo è un passaggio che non abbiamo ancora affrontato ma è inevitabile, in un sistema basato sull’uso solidale delle risorse deve entrare su questo tema, sull’uso solidale di una risorsa comune. Quindi quando, non so, in pediatria siamo particolarmente sensibilizzati, ma insomma terapie semi sperimentali costosissime, enzimatiche, genetiche, talmente costose e con relativo, scarso beneficio, è logico, dal mio punto di vista etico, che vengano sottoposte a un quesito quanto meno.
D:	Certo. Vi trovate molto spesso ad affrontare queste questioni diciamo di micro allocazione delle risorse?
R:	Micro ma anche macro; il primo caso, quello pediatrico, che è quello che conosco di più, era tale, erano terapie da centinaia di migliaia di euro l’anno, alcune centinaia di migliaia in un bambino che poi è deceduto, quindi… e qualcuno potrebbe dire che abbiamo buttato via quelle risorse che potevano essere usate per bambini con maggiore probabilità di sopravvivenza.
D:	Sì, sono temi difficilissimi.
R:	Molto.
D:	Sono temi proprio difficili. Ascolti, le domande sono finite, io la ringrazio ancora per la sua partecipazione, la sua disponibilità a partecipare a questo progetto di ricerca, le chiedo se per caso ha qualche ulteriore pensiero o commento da condividere, magari se le viene in mente qualche argomento che le sta a cuore, che invece non è uscito con le domande che le ho fatto.
R:	Direi che è uscito quasi tutto; finisco nel dire che quello che ho spero capito è che questi Comitati dovrebbero essere diffusi non dico ovunque, ma insomma un po’ più capillari, anche perché assieme al concetto di tempestività ci sta la prossimità, perché uno può… e lasciando perdere, si fa per dire lasciando perdere, lo dico di nuovo da pediatra, un tema che non so, come pediatra non penso che affronterò mai, ma che negli ultimi tempi è soverchiante, che è quello del suicidio medicalmente assistito, che è un tema enorme, dove il Comitato Etico deve, non bene o male, cioè gli tocca diciamo, perché sono… è un tema molto, molto pesante per gli operatori e per le famiglie. Quello è uno spazio, se aumenteranno quel tipo di richieste, non oso immaginare, al di là del carico di lavoro, della gravità di quel lavoro.
D:	Certo. Ascolti, basta, abbiamo finito con le domande, le chiedo un’ultima cosa, cioè se posso eventualmente risentirla se leggendo le trascrizioni mi rendo che ci sono elementi magari che mi sono poco chiari, ho bisogno di qualche approfondimento.
R:	(? 52:22) accordarci, io sono in ferie dopo Ferragosto, da Ferragosto.
D:	No, tanto le trascrizioni le avremo da settembre, quindi di sicuro non la disturbo in ferie.
R:	Sono in ferie fino al 12 settembre.
D:	Va bene, va bene, non c’è nessun problema.
Fine trascrizione 00:52:35
```
