# Supplementary material for: How to implement a clinical ethics committee in an oncological research hospital: Qualitative results from a process evaluation study using normalization process theory (EVACEC)
Source: PLoS One. 2025 May 6;20(5):e0318870. doi: 10.1371/journal.pone.0318870 (PMC12054913; doi:10.1371/journal.pone.0318870)
Supplement: S5 File — Interviews transcripts_raw data: this folder contains all the raw data used in this study, the interview transcripts (in original Italian). (ZIP) [file pone.0318870.s005.zip › S5_Interviews transcripts_raw data/10CM.doc]

```
Inizio trascrizione 00:00:00
D: 	Se ha voglia di raccontarmi la sua esperienza in ambito di etica/bioetica.
R:	Allora, io sono laureata in Giurisprudenza, vengo dal terzo settore, quindi dalla situazione di volontariato; mi è capitato spesso di dover approfondire questioni che adesso consideriamo etiche, magari nel momento in cui le vivi ne hai meno consapevolezza. Poi da parlamentare soprattutto, dovendo seguire in Commissione Affari Sociali e Sanità diversi temi che riguardano il tema dell’inizio e della fine della vita, uno si chiama un po’ ad approfondire; ho studiato, ho fatto un corso insomma, ho continuato a occuparmene ecco. Nasce un po’ così, dall’intervento del caso concreto e delle esperienze istituzionali.
D:	Benissimo. Invece la sua esperienza con il CEC, ha voglia di raccontarmi qualcosa?
R:	Allora, mi conoscevano in diversi nell’azienda IRCCS di Reggio Emilia, in particolare Ludovica De Panfilis, quindi mi hanno chiesto se volevo stare in questa esperienza sperimentale della Regione sul CEC, ho detto di sì e mi sembra che l’esperienza sia molto interessante, perché intreccia proprio il caso concreto, clinico, al letto del paziente, quindi una persona con determinate caratteristiche e aspettative, richieste e altro, con i principi di carattere generale. Perché quando stai molto coi principi perdi di vista la concretezza e quando sei a seguire il singolo caso spesso entrano in gioco fattori, tipo quelli psicologici o clinici, che distolgono l’attenzione dal tema etico, o non viene colto dall’operatore. Quindi è un’esperienza dove si cerca di tenere insieme i due aspetti; non è facile, non è facile anche per gli operatori comprenderlo, perché significa fermarsi un attimo a riflettere, però è molto interessante.
D:	Benissimo. Volevo chiederle anche un po’ quali sono le motivazioni che l’hanno spinta a far parte del CEC.
R:	Ma sicuramente appunto il desiderio di misurarsi con i singoli casi, non le nascondo che paradossalmente la pandemia con l’uso maggiore del ricorso a strumenti come questi, quindi in collegamento e che non richiedono la presenza, ha aiutato, perché io vivo fuori Bologna, dovrei prendere diversi treni per andare a Reggio Emilia e questo mi aveva forse un po’ scoraggiato, anche se si tratta di una riunione al mese, gli impegni sono tanti. Però sì, per il motivo principale, quello di misurarsi con il singolo caso in una dimensione multiprofessionale. Anche questo è importante, non sono solo medici, non sono solo specialisti di bioetica, non sono solo giuristi e il confronto è tra professioni diverse.
D:	Certo. Va bene, allora visto che abbiamo visto un po’ la sua esperienza, il suo punto di vista sul CEC, le chiedo, le faccio un po’ di domande invece che hanno proprio il CEC come argomento. Secondo lei qual è il suo ruolo, il ruolo del CEC all’interno dell’azienda?
R:	Dunque, le faccio una premessa: io considero molto negativamente la decisione del Governo, dal Ministro Speranza giustificata da motivazioni pratiche, di dare la valutazione sui casi di suicidio assistito ai Comitati per la ricerca e la (? 04:51) alla sperimentazione dei farmaci dei dispositivi. Perché il taglio è completamente diverso e l’incoraggiare il ricorso all’etica clinica sarebbe stato sicuramente meglio, perché qui non si tratta di dare applicazioni a norme, è chiaro che le norme e i principi di riferimento ci sono, però la complessità della vita e delle singole casistiche è tale che ti implica, ti richiede proprio un calarsi nella situazione e soprattutto quando i casi riguardano bambini, ma non solo bambini, sei costretto a prendere in considerazione una serie di elementi e non ti puoi accontentare del rispetto (? 05:44) le procedure. E quindi da questo punto di vista penso che un comitato di etica nella clinica sia più utile. Naturalmente questo richiederebbe una nostra messa a disposizione oraria più ampia, perché un medico non può aspettare, spesso le decisioni vanno prese nell’arco di una settimana, dieci giorni, ecco, che è già un tempo, dal punto di vista del malato, non breve. E quindi un consolidamento dei Comitati etici con una loro strutturazione, perché se devi lavorare molto hai bisogno di una struttura più forte. Però ci vuole una (? 06:32) lavoro di affiancamento dei professionisti che fa loro comprendere che il comitato non è una sede di giudizio, ma è un luogo dove si aiuta a prendere delle decisioni che attengono anche alla dimensione etica.
D:	Corretto.
R:	Questa è la cosa più importante.
D:	Certo. E invece stando sempre più aderenti al CEC, a quello che a oggi fa nell’azienda, secondo lei in che cosa è diverso da altri servizi promessi, sempre all’interno dell’azienda e qual è invece la sua specificità?
R:	Allora, intanto bisogna dire che l’IRCCS di Reggio Emilia ha un’attitudine, un approfondimento sui temi di bioetica che sono rari in Italia e va merito di chi le ha guidate, di chi la guida. Perché l’esistenza stessa di un’unità operativa di bioetica è una rarità, la presenza di uno sportello rivolto al pubblico aperto una volta alla settimana per la compilazione delle DAT è una cosa di grandissima utilità e la possibilità che la responsabile dell’unità operativa possa affiancare i professionisti nelle sedi di esame dei singoli casi è anche questa una rarità molto importante. Il CEC subentra quando la questione è più complessa, non si tratta di portare, essendoci il parare del bioeticista, ma richiede la costruzione di una risposta, per ottenere la quale ci vogliono professionalità diverse e non basta (? 08:26). Noi pensiamo che la bioetica sia una cosa ferma, stabile, un po’ religiosa magari. Che la religione ha i suoi principi, li studiamo, eccetera. Invece i tempi di oggi, tempi laici, in un regime democratico, la bioetica richiede uno scopo continuo di mediazione di ricerca di soluzioni. Non sempre quindi è possibile farlo in solitudine, è necessario invece rivolgersi alla sede o al luogo di approfondimento. Noi abbiamo un po’ l’abitudine a pensare che appunto ci fosse una fede unica ed è un punto di riferimento etico per tutti; poi al limite si poteva dissentire, ma solo per dire di no, ecco. Invece noi siamo chiamati a costruirci una dimensione bioetica, di volta in volta, e questo è molto difficile.
D:	Sono d’accordo, sì anche perché in una società come la nostra è pur vero che anche ammettendo che i principi rimangano invariati, quello che non rimane invariato sono in ambito sanitario le possibilità terapeutiche, eccetera, che comunque aprono indubbiamente nuove sfide. Ascolti, e secondo lei invece qual è l’obiettivo del CEC, in che modo agisce nel contesto locale? Qualcosa me l’ha già detto su questo, ma se vuole aggiungere altro.
R:	L’obiettivo è quello di supportare i professionisti in questa fase, di contribuire nei momenti in cui si elaborano documenti rivolti a tutti e non il singolo caso ad appunto cercare e definire le posizioni bioetiche nel tempo di oggi, coi problemi di oggi e quindi è sede e luogo di approfondimento; il terzo che non abbiamo ancora esplorato a fondo, e secondo me invece dovremmo nel futuro approfondire, è quello di parlare alla cittadinanza, perché c’è una grandissima confusione, legata a messaggi troppo veloci, oppure a volte più forti. Quindi aiutare le persone a comprendere e dare loro una consapevolezza.
D:	Sì, sono d’accordissimo, sul fatto che ci sia molta confusione, assolutamente. Ascolti, mi parla un po’ del suo lavoro in particolare, all’interno dei servizi che offre il CEC. Cioè, di cosa si occupa lei in prima persona?
R:	Ma non c’è una mia specializzazione specifica, non rappresento né la voce degli utenti, né quella dei professionisti, ci sono figure che hanno invece più chiaramente questo taglio. Quindi se vuole un po’ dell’esperienza di chi ha girato molto per l’Italia, visto situazioni molto diverse e quindi ha una visione non tutta legata alla dimensione locale e aziendale. E anche questo è importante, perché problemi di questo tipo sono comuni, quindi da un certo punto di vista, da un lato questa esperienza mi è utile proprio quando insegno o parlo in altre sedi, in altri luoghi, dall’altra aver visto e vissuto in altre sedi, in altri luoghi, mi permette di portare questa competenza nel progetto. Poi è chiaro che io ho una base giuridica, con la costruzione di leggi, quindi il mio taglio è giuridico. Questa è una delle cose che abbiamo ragionato da poco; cioè la valutazione etica non può fare a meno, nonostante è democratico, della conoscenza delle norme, non è che noi possiamo dire a un medico “vai contro la Legge”. Non possiamo, non sarebbe accettabile. Nello stesso tempo è più libera, più ampia, non può esserne schiava, noi non siamo né gli avvocati né i magistrati, il nostro compito non è spiegare quali sono le responsabilità professionali al medico, la nostra dimensione è quella di aiutarlo a ragionare che cosa sia bene, che cosa sia male per il paziente in quel momento, in quel dato contesto. Quindi la mia competenza, che è più giuridica che altro, però viene continuamente rimessa in discussione da un confronto multiprofessionale così ampio.
D:	Invece cosa pensa del valore che ha un servizio come quello che offre il CEC per il lavoro dei professionisti sanitari? Cioè secondo lei i professionisti sanitari come lo vivono, che valore pensano che abbia?
R:	Ma questo bisogna ovviamente chiederlo a loro e lei (? 13:46)
D:	Indubbiamente.
R:	Certamente. Ecco, direi che dobbiamo avere un ruolo più di affiancamento che di giudici e nello stesso tempo esprimere delle posizioni, con cui il professionista si misura. Farsi domande che magari non si è fatto, per esempio anche i più legati, affezionati ai pazienti non sempre riescono a fare lo sforzo di capire che cosa vuole il paziente, come sia importante questo aspetto. Ė una forma di empatia, di volontà pure, non è negativo in sé questa preoccupazione, sono in genere in medici più tra virgolette umani, quelli considerati più attenti ai bisogni di una persona, che hanno questo atteggiamento. Però è utile invece richiamarli a una presa in carico complessiva; a me piacere dire non stai curando una patologia, non stai curando una gamba malata o una persona operata di tumore, stai curando una persona che ha una patologia, non puoi guardare solo a quella malattia che cosa fare, devi guardare a tutta la dimensione personale.
D:	Certo, sono d’accordo. Ascolti, pensando alle attività che il CEC svolge, in che modo, da quello che ha potuto vedere, sono state integrate nel contesto locale? Pur da esterna appunto, perché non essendo lei interna all’ospedale.
R:	Mi sembra che siano state integrate bene, soprattutto la figura della bioeticista, dell’unità operativa mi sembra che sia entrata abbastanza. Naturalmente non bisogna pensare che sia fatta una volta per sempre; siccome gli operatori cambiano, ruotano, si trasferiscono, vanno in pensione, eccetera, questo lavoro di sensibilizzazione, di costruzione di relazioni è quotidiano, non può essere archiviato. L’ho fatto due anni fa, no, va fatto ogni anno.
D:	Io non so, perché appunto non facendo parte del CEC un po’ mi chiedo questo cosa, se voi come membri, soprattutto membri esterni all’ospedale, abbiate un po’ a che fare attraverso il CEC con i professionisti sanitari, se li vedete in audizione o alle formazioni?
R:	Di audizione pochi. Ne abbiamo fatte poche. Sicuramente una delle cose su cui adesso ci siamo resi conto che farne di più, lei tenga per conto che più viene richiesta velocità nella decisione, più difficile è una procedura che preveda anche molte audizioni. Dal punto di vista esterno invece è in corso quest’anno, l’attività di formativa che è stata fatta è stata molto interessante. E ci sono molti professionisti, quindi vuol dire che aveva inciso.
D:	Va bene. Ci sono stati dei limiti secondo lei, in questo processo di integrazioni, che possono essere limiti organizzativi interni oppure esterni al servizio, oppure ancora limiti economici, che lei percepisce rispetto al CEC?
R:	Ci sono sicuramente tutti questi limiti, compreso quello economico, perché è chiaro che se noi lavoriamo una volta ogni tre settimane è una cosa, ma se ci viene chiesto di più sarà problematico far tutto gratuitamente. Detto ciò, secondo me quello che manca non dipende dall’azienda ma dalla normativa. Cioè se la Regione almeno, non dico a livello nazionale perché adesso è in piena crisi, temo che non ce se ne occuperà per un po’; ma nel livello regionale li regolamentasse, gli desse un riconoscimento istituzionale, questo renderebbe i rapporti un po’ più facili.
D:	Certo, lo si può capire. Parliamo un po’ delle potenzialità invece che ha il CEC. Quindi possibilità che lei vede che ci possano essere, magari strade da implementare nel futuro.
R:	Allora due le vedo abbastanza chiaramente; una è la costruzione a cui abbiamo appena iniziato di una rete e delle esperienze che ci sono in questo campo in Italia, perché ci permette di migliorare e di diffondere lo strumento, questo per quello che riguarda l’esterno. L’interno io penso che possiamo fare di più e vedo ancora qualche difficoltà più dentro l’ospedale. Abbiamo avuto forse un numero pari di richieste dalla medicina del territorio rispetto a quella ospedaliera, mi aspetterei un po’ di più di richiesta dal punto di vista dell’ospedale. Non sono così sicura che vada tutto liscio e non si pongano mai dubbi ecco.
D:	E secondo lei mancano… mancano no, però insomma le richieste sono meno di quelle che potrebbero essere, per quali ragioni?
R:	Non saprei dirle esattamente. Probabilmente i ritmi sono maggiori, forse anche il lavoro d’equipe aiuta di più, chi è sul territorio, soprattutto il medico di famiglia, si sente più solo. Però il rischio è che non ci si faccia neanche la domanda, cioè un po’ si evade l’emergenza.
D:	Certo, si segue un po’ la prassi.
R:	Si segue la prassi e l’emergenza uccide l’intelligenza, dice il proverbio, quindi. Se viviamo sempre sotto risorse scarse c’è poco tempo per fare considerazioni più approfondite.
D:	Certo, questo senza dubbio. Passiamo un po’ invece alla valutazione del servizio. Secondo lei alla luce di questi circa sedici mesi di attività, come si è comportato il CEC, come valuta il servizio che è stato implementato?
R:	La mia valutazione è estremamente positiva. Non mi sembra che ci siano, oltre alle cose che ho già detto, dei limiti intrinsechi (? 21:12) significativi ecco. Dipendono più dalla condizione esterna, come dicevamo prima ecco, e poi ci vorrà sicuramente del tempo per diffondere questa esperienza.
D:	Rispetto quindi alle attività che magari svolgevate di più all’inizio, è una cosa che mi è stata detta, che soprattutto nei primi periodi lavoravate molto sulle policy, sulla stesura di documenti, poi invece il lavoro si è più focalizzato su un’analisi dei casi, il supporto etico in casi specifici. Mi corregga se sbaglio, queste sono cose che mi hanno…
R:	No, è corretto, secondo me ci aiuterà in futuro scrivere documenti migliori. Lei tenga però conto che quando si è lavorato condizionati dal Covid è tutto molto (? 21:58). Per forza (? 22:01) in generale insomma, proprio ha reso più difficile i rapporti in tutti i casi.
D:	Certo, da un lato, come diceva prima…
R:	Ecco, proprio il contesto che c’è stato è particolare.
D:	Certo, da un lato, come diceva lei prima, forse ha facilitato fra virgolette la partecipazione dei membri soprattutto esterni, dall’altro però chiaramente rallenta un po’.
R:	Esatto, perché non puoi interloquire direttamente.
D:	Certo, no, si capisce, chiaro. E secondo lei fra diciamo le varie attività che avete svolto, che cosa fa particolarmente presa, cosa funziona di più per gli operatori sanitari? Cioè com’è che siete richiesti di più?
R:	Ma anche su questo preferirei che si esprimessero loro. Potremmo essere visti più di aiuto nel lavoro di equipe, che tende a essere un po’ più autoreferenziale e quindi insomma questo potrebbe aiutare. Lei tenga conto che una parte dei membri sono interni, quindi quello che andiamo a costruire è un parere che in qualche modo all’interno dell’azienda c’è già ecco, che viene valorizzato e questo è positivo. A volte potrebbero esserci problemi, ma lo dico più in prospettiva, se noi venissimo a dire cose che incidono sull’organizzazione. Le faccio un esempio: il tema dell’apertura dei reparti anche ai parenti, anche in epoca Covid o post Covid come quella che stiamo vivendo, secondo me è un tema molto importante e sul giornale di oggi c’è scritto (? 24:01) di Bologna è uscito su questo e la Regione dice sì, ha ragione, bisognerà stare più attenti. Noi è due anni che lo poniamo e probabilmente qui si scontra appunto con le difficoltà organizzate a gestirlo. Però il tema c’è.
D:	Indubbiamente, sì.
R:	(? 24:24). Quindi magari forse bisognerà trovare, non so, strumenti diversi per far conoscere anche la nostra opinione in merito a queste questioni.
D:	Certo. Invece anche se in realtà ha già almeno parzialmente coperto questa risposta, ma la domanda gliela faccio ugualmente; in che cosa secondo lei il CEC potrebbe essere migliorato? Se ha altro da aggiungere, perché tante cose me le ha già dette.
R:	Non so, una presenza maggiore e con una valutazione di riconoscimento anche esterno (? 25:09-25:13) all’interno. Quindi questo aiuterebbe, secondo me. E credo che finisci a dover essere molto più sotto osservazione quando dai le risposte, eccetera, quando è istituzionale, però allo stesso tempo è importante.
D:	Sì, dà sicuramente fra virgolette un’autorità diversa all’ente proprio, certamente. Ascolti, le domande sono finite, rispetto a quello che è emerso dall’intervista le chiedo se ha ulteriori pensieri, commenti da condividere o se sente che ci sono magari argomenti che non abbiamo toccato che invece sono importanti da affrontare.
R:	No, mi sembra che le domande fossero ampie, a 360 gradi e avete quindi insomma guardato un po’ tutti i punti di vista. Quindi no, non sento la necessità di integrare, al momento.
D:	Va benissimo.
R:	Credo che un lavoro come il suo potrebbe aiutarci proprio a far conoscere di più quella che è l’esperienza del CEC, pubblicare, eccetera, che bisogna anche fare quel lavoro lì, è importante.
D:	Certo. Ascolti, io intanto la ringrazio ancora per la sua disponibilità, le chiedo anche se possibile, disponibilità a risentirla eventualmente se rileggendo la trascrizione dell’intervista ci dovessimo accorgere che magari c’è qualche punto non chiaro. Okay, allora interrompo l’intervista, poi le faccio due domande due.
Fine trascrizione 00:27:03
```
