# Supplementary material for: How to implement a clinical ethics committee in an oncological research hospital: Qualitative results from a process evaluation study using normalization process theory (EVACEC)
Source: PLoS One. 2025 May 6;20(5):e0318870. doi: 10.1371/journal.pone.0318870 (PMC12054913; doi:10.1371/journal.pone.0318870)
Supplement: S5 File — Interviews transcripts_raw data: this folder contains all the raw data used in this study, the interview transcripts (in original Italian). (ZIP) [file pone.0318870.s005.zip › S5_Interviews transcripts_raw data/11CM.doc]

```
D: 	…intervista un po’ particolare, anche per il tipo di domande, perché abbiamo impostato questo progetto seguendo un framework particolare, che è quello della Normalization Process Theory, che quindi anche nel corso delle interviste…
R:	Normalization…?
D:	Normalization Process Theory.
R:	Okay.
D:	Ė un framework di ricerca che anche nel corso di queste interviste prevede una serie di temi da affrontare, che riguardano proprio l’implementazione di un servizio; quindi anche come domande magari ce ne sono alcune un po’ più specifiche e forse anche un pochino ripetitive su quella che è stata, su cosa ne pensi tu di questo servizio, e ci tenevo ecco a specificarti questa cosa qui, che forse alcune sono un po’ ripetitive, ma sono proprio in linea con la teoria che abbiamo adottato. Quindi partirei col chiederti, col farti una domanda invece molto generale e ti chiedo di raccontarmi un po’ la tua esperienza con il Comitato per l’Etica nella Clinica.
R:	Un’esperienza recente, relativamente breve, interessante per me che ho invece una lunghissima esperienza di Comitati etici per la sperimentazione dei medicinali, anche perché sono abbastanza vecchio in questo settore da aver visto l’evoluzione di questi Comitati etici, che all’inizio erano comitati che non avevano solo questa ristretta specificità, ma si occupavano anche delle questioni etiche nella clinica, quale più e quale meno. Poi progressivamente, per effetto soprattutto delle normative nazionali e poi anche europee, si è molto diciamo così ristretto l’ambito di azione dei Comitati per la sperimentazione dei medicinali a questo campo poi esteso. Adesso siamo in una fase di nuova e rapida evoluzione con l’applicazione del nuovo Regolamento europeo; per cui insomma da tempo e in cui si è visto una cosa sostanzialmente, che sempre più e ancora più nel prossimo futuro, i Comitati etici per la sperimentazione dei medicinali, dei dispositivi, eccetera, sono, ma addirittura per la normativa devono essere lontani, molto lontani, addirittura estranei rispetto al luogo dove si fa la sperimentazione. Questo è l’opposto di quello che era lo spirito con cui erano nati i Comitati etici tra la fine degli anni Settanta e i primi anni Ottanta; invece di essere a fianco dei medici e dei malati, nel momento in cui si ponevano problemi etici, compresi quelli della sperimentazione. Quindi io ho visto con molto favore la nascita di questo Comitato, come seguo con attenzione anche quelli che ci sono, ce n’è uno solo in Emilia-Romagna ma ce ne sono diversi in Toscana e sono molto interessato a vedere come l’attività di questo comitato complementare è gran parte sostitutiva di quelli che abbiamo conosciuto riesca a svilupparsi. Purtroppo si intrecciano tantissime questioni e abbiamo interlocutori, compreso il Ministero, l’AIFA, cioè che non sempre hanno le idee chiare e questo però è una costante. E quindi per esempio la questione posta dalla Corte Costituzionale sul suicidio assistito, anche lì si è visto come la Corte abbia indicato i Comitati etici genericamente, se proprio veramente non è il loro mestiere e poi su questo si pongono, si sostengono posizioni che sono comprensibili dal punto di vista così puramente astratto, ma assurde quando si pensa di affidare questo tipo di compito a un Comitato per la sperimentazione dei medicinali, che come abbiamo detto è addirittura istituzionalmente lontano da dove viene. Quindi l’esperienza finora è stata quella di una discussione tra diverse competenze, sempre interessante e sempre di alto livello. Particolare e interessante anche nell’ultima occasione la difficoltà, ma anche la chiarezza di distinguere il livello normativo o giuridico da quello etico. Sono due approcci, entrambi fondamentali, che hanno però punti di vista, punti di partenza e punti di arrivo che possono essere molto diversi, e che vanno… perché in questa cosa si deve essere insieme consapevoli e avere molta chiarezza. Se mi dilungo troppo fermami.
D:	No, anzi. Allora, adesso ti faccio qualche domanda più specifica invece sul CEC e ti chiedo secondo te qual è il ruolo del Comitato per l’Etica nella Clinica all’interno dell’azienda.
R:	Dunque ovviamente quello che sta già svolgendo, cioè di ricevere le richieste di pareri oppure direttamente da persone coinvolte da vario titolo, operatori sanitari, anche teoricamente potrebbero essere pazienti, anche se credo che questo non sia ancora avvenuto. Ma anche diciamo così pareri richiesti a singoli responsabili come per esempio il caso del… come si chiama l’ufficio della (redacted)?
D:	L’unità di bioetica?
R:	L’unità di bioetica, ma anche l’unità di cure palliative; cioè che ricevano delle richieste che giustamente sentono il bisogno di coinvolgere il Comitato per l’Etica nella Clinica perché è multidisciplinare e quindi sentono la necessità di avere, multidisciplinare in maniera molto intelligente, e sentono la necessità di questo coinvolgimento. Per cui questo è il modo con cui le cose si stanno svolgendo e mi sembra che le cose stiano da questo punto di vista andando bene, si stanno svolgendo bene, mentre è molto difficile l’interlocuzione con le varie istituzioni, nazionale, regionale, con gli altri Comitati etici, per esempio. Un caso particolare, che è un po’ un mio pallino, è che io ritengo che una delle applicazioni più immediate e dirette dei Comitato per l’Etica nella Clinica è quello dei cosiddetti compassionevoli. Compassionevoli non sono sperimentazioni e non lo devo essere, anche se a volte sono sperimentazioni mascherate; sono situazioni in cui un medico sente la necessità dell’uso di un farmaco non ancora disponibile per la prescrizione, e secondo una normativa che è anche questa molto farraginosa e sostanzialmente sbagliata, ha bisogno per questo di avere l’approvazione del Comitato etico. In realtà non dovrebbe essere così, dovrebbe essere non un’approvazione ma dovrebbe essere un parere consultivo, che il clinico sente il bisogno - il clinico, l’equipe dei curanti, perché dovrebbe essere non un singolo ma l’equipe dei curanti - sente il bisogno di avere altre competenze per decidere se in quel caso specifico - e il caso specifico quindi vuol dire vicinanza, prossimità, non lontananza, perché è un caso specifico - coinvolgere il Comitato per l’Etica. E questo dovrebbe essere l’iter, che purtroppo confligge con quello che invece avviene… Addirittura attualmente, per alcune terapie che non sono terapie, che non sono farmaci, l’AIFA chiede di coinvolgere il Comitato per la sperimentazione dei farmaci, ma non pone… con un parere che non è vincolante, è puramente consultivo. Questo è un caso in cui è evidente che non è quella la sede giusta e che mancano le competenze. Ma appunto come sempre è difficile che tutti gli interlocutori e tutti i livelli istituzionali riescano ad avere una sufficiente chiarezza su chi fa cosa, perché lo fa, con quali competenze, eccetera.
D:	Ecco, infatti, rispetto a questo volevo chiederti se e in che modo, secondo te, questo Comitato per l’Etica nella Clinica è diverso da altri servizi presenti in azienda e qual è un po’ la sua specificità.
R:	Per esempio quali altri servizi?
D:	Altri servizi, ma un po’ mi pare che tu me l’abbia anche un po’ anticipato, per esempio il Comitato etico per la sperimentazione, poi c’è anche l’unità di bioetica, però questi servizi in generale, ecco.
R:	Sì, appunto qui rientriamo in quello che mi avevi anticipato della ripetitività sì. Diciamo che le cose che ho detto penso che siano già quelle che.
D:	Okay. Ecco, invece ti chiedo quali sono le motivazioni tue personali che ti hanno spinto a prendere parte a questo servizio.
R:	Soprattutto il fatto che quando (redacted) ancora me l’ha proposto insomma, io ho preso parte fin dall’inizio alla messa a fuoco e all’ideazione della necessità di un Comitato di questo genere, quindi mi sembrava doveroso, anche se avevo già allora e ho tuttora altri due incarichi in Comitati etici e quindi un terzo poteva anche essere, per me che sono un pensionato e non ho voglia di fare il pensionato, poca, gravoso, però mi sembrava che fosse incoerente non partecipare. Cioè incoerente rispetto al percorso fatto fino a quel momento e continuo a pensare così, per cui ho accettato volentieri e sono contento di averlo fatto.
D:	Okay. Poi un’altra domanda che ti volevo fare, se hai voglia di aggiungere qualcosa rispetto a quanto detto prima, è un po’ in che cosa consiste il tuo lavoro in questo servizio; se ti va di aggiungere qualcosa.
R:	Sì, il mio lavoro in questo servizio cosa consiste: se devo dire la verità soprattutto nel capire. Non è facile capire, ed è sempre… che vuol dire ascoltare, ascoltare anche punti di vista che possono sembrare divaricati, rispetto a quello che è il mio spontaneo, ascoltare e cercare di arrivare a una composizione interna di questa divaricazione. Per esempio nel caso che discuteremo nella prossima – sarà domani – sarà importante, è stato importante ascoltare le varie posizioni per capire dove eventualmente poteva nascondersi il rischio di una scarsa chiarezza. Quello che è fondamentale nella discussione etica, che per me è una discussione che teoricamente dovrebbe sempre arrivare a una posizione, a un parere, a una decisione all’unanimità, perché il punto di vista non è… cioè perché il valore in gioco non è un’ideologia o un pensiero, una legge, un qualcosa, ma è il bene del paziente e sul bene del paziente in genere. Questa è la mia esperienza e quella che mi ha appassionato di più nella mia storia, io ho cominciato a stare nei Comitati etici nel (redacted), quindi sono (redacted) anni; quello che mi ha appassionato di più è proprio questo, di vedere come anche posizioni che partono apparentemente inconciliabili, poi quando si tratta di discutere nel concreto, nel caso concreto di qual è il bene del paziente si trovano d’accordo, se ne discutono tra virgolette onestamente dal punto di vista intellettuale. Quindi il mio compito, quello che sento il mio compito è quello di ascoltare e capire e poi contribuire con quello che ho creduto di capire.
D:	E adesso invece ti chiedo che cosa ne pensi del valore di un servizio come quello del Comitato per l’Etica nella Clinica per un po’ al tuo lavoro e quello dei tuoi colleghi.
R:	Il mio lavoro oramai è questo, nel senso che… per il lavoro dei colleghi credo che possa essere importantissimo; quando finalmente si scoprirà la disponibilità di qualcosa come questa e verrà inteso come un valore aggiunto anche dal punto di vista delle decisioni cliniche rispetto a quello che avviene oggi, potrebbe essere veramente un grande sollievo per tutti gli operatori e anche per i pazienti. Purtroppo la strada è lunga e difficile e non è affatto detto che ci si riesca o che ci si riesca dappertutto; per cui c’è un lungo percorso ancora da fare, credo.
D:	Ecco, rispetto a questo, adesso ti faccio delle domande un po’ più specifiche proprio sul processo insomma che c’è stato e che c’è in questi mesi di implementazione di questo servizio e ti chiedo quindi secondo te, dal tuo punto di vista, pensando alle attività svolte dal Comitato per l’Etica nella Clinica, come sono state integrate nel contesto locale, se sono state integrate?
R:	Per esempio, faccio un esempio banale, di due situazioni in cui sono dentro in entrambe, io sono nel Comitato etico di Area Vasta Nord Est e non è stato integrato per niente, proprio non è vissuto neanche né come un’opportunità, né come una risorsa, né come uno strumento, addirittura è vissuto, alcuni membri di quel Comitato quasi come un’entità priva di base giuridica. Questo è stato detto, è un’affermazione che ho ascoltato, con il mio (? 18:37), per cui insomma per questo dico che è ancora tutto molto difficile. In altre Regioni le cose vanno molto meglio, qui noi purtroppo c’è solo questo Comitato in tutta la Regione, viene pensato come l’unico da usare a livello regionale, ma è sbagliato, perché sono Comitati che devono essere istituzionali. Istitutional Review Board era il nome americano negli anni Settanta, ma è così, perché devono vivere, crescere, svilupparsi nel rapporto, non dico quasi quotidiano di chi lavora in quel posto.
D:	Ti faccio una domanda perché non ho capito molto bene prima, ed è il Comitato Etico AVEN che hanno detto che questo Comitato per l’Etica nella Clinica è privo di una base giuridica? Ho capito esatto, è così?
R:	Okay. Questo veramente forse sto violando un vincolo da riservatezza ma senza… però lo dico così diciamo.
D:	No, è indicativo sicuramente di come viene percepito, sì. Quindi ti chiedo, se…
R:	Ma perché io proponevo per esempio “però certe cose di cui stavano discutendo, forse sarebbe stata più opportunamente discussa dal Comitato per l’Etica nella Clinica”, qualcuno ha detto “quello è una base più (? 20:16)”
D:	Quello è…?
R:	Ma forse semplicemente dicendo il fatto che il Ministero o l’AIFA non riconosce questa funzione e quindi quella confusione di cui parlavo prima poi ovviamente crea dei problemi appunto anche di integrazione di rapporti istituzionali anche locali.
D:	Okay. E ci sono secondo te degli altri limiti di tipo magari organizzativo, interno, anche di tipo economico magari, che percepisci rispetto a questo diciamo processo di integrazione del Comitato per l’Etica nella Clinica?
R:	Sì, ci sono; per quanto voi facciate un lavoro eccellente, ovviamente ci sono, chiaramente problemi organizzativi e anche economici, cioè avere… diciamo che il nostro Comitato per l’Etica nella Clinica si sta muovendo in una fase che appunto è sperimentale e quindi questo è già un limite, perché appunto forse anche questo contribuisce a dire che non c’è una base giuridica per affidare determinate discussioni. Però non credo che… questi limiti in questo momento non sono il problema principale, sono un problema assolutamente secondario. Il problema principale è la non chiarezza istituzionale a tutti livelli rispetto a problemi importanti.
D:	E invece a livello di potenzialità, rispetto a questo servizio?
R:	Questo l’ho detto prima, la potenzialità è enorme, sì, una potenzialità enorme. Ma alla fine assolutamente il mio punto di vista è che chi ne ha le forze vada avanti su questa strada.
D:	Okay, adesso ti faccio… queste sono le ultime due domande invece, che sono un po’ più conclusive e ti chiedo, rispetto a quanto fatto in questi ormai venti mesi di attività, come valuti in generale il servizio che è stato implementato?
R:	Molto bene, molto bene per quello che è la mia esperienza; con tutti i limiti che ho già detto e che sono in gran parte esterni all’attività del Comitato; con tutti i limiti delle occasioni perse per utilizzare al meglio una risorsa che è ancora sperimentale, però non vengono spalancate le porte, ma semmai tenuta in sordina.
D:	E quindi pensando a delle modalità con cui il CEC potrebbe essere migliorato, ti viene in mente qualcosa?
R:	Avendo finora detto che i problemi sono soprattutto esterni, penso che si debba lavorare su quelli, compreso il fatto che mentre nel CEC ci sono persone che sono anche influenti nella loro attività e competenze, possono continuare a spingere nella direzione giusta.
D:	Okay, perfetto. Allora noi abbiamo concluso questa intervista. Ti ringrazio davvero tanto per il contributo e prima di concludere ti chiedo se magari ci sono degli aspetti che ti farebbe piacere condividere rispetto alla tua esperienza, rispetto a quello di cui abbiamo parlato oggi, che magari non sono emerse durante questa intervista.
R:	No, direi di no. Io niente, ti ringrazio per questa intervista, se senti la necessità puoi fare un editing, cioè puoi accorciare alcune mie chiacchierate troppo lunghe.
D:	Ma assolutamente no, sono state interessantissime, anzi è un gran punto di vista, adesso questo andrà al di fuori del… però anche insomma vedere questo confronto anche con la tua esperienza nel Comitato etico, l’altro, è veramente prezioso, anche ai fini proprio di questa ricerca. Sarà veramente interessantissimo mettere a confronto tutte queste interviste, perché sono tutte esperienze diverse, quindi penso che mi divertirò in fase di analisi. No dai, scherzi a parte… […] Va bene, ancora grazie. […]
Fine trascrizione 00:27:56
```
