# Supplementary material for: How to implement a clinical ethics committee in an oncological research hospital: Qualitative results from a process evaluation study using normalization process theory (EVACEC)
Source: PLoS One. 2025 May 6;20(5):e0318870. doi: 10.1371/journal.pone.0318870 (PMC12054913; doi:10.1371/journal.pone.0318870)
Supplement: S5 File — Interviews transcripts_raw data: this folder contains all the raw data used in this study, the interview transcripts (in original Italian). (ZIP) [file pone.0318870.s005.zip › S5_Interviews transcripts_raw data/13CM.doc]

```
D: 	Partiamo, sperando che non ci siano altre interruzioni. Ti stavo dicendo, è un’intervista che può essere che sia anche un po’ ripetitiva, ma questo è dovuto al fatto che per rimanere aderenti a questa traccia ci sono una serie di domande che possono essere appunto un po’ ripetitive. Quindi io comunque partirei dal farti questa prima domanda, che è una domanda generale, e ti chiedo di raccontarmi un po’ qual è la tua esperienza con il Comitato per l’Etica nella Clinica.
R:	Okay, più o meno quanti minuti vuoi che prenda? Cioè una risposta breve o…
D:	Quello che vuoi condividere.
R:	Come voglio.
D:	Io non ho problemi di tempo, ho visto che in generale dura più o meno una mezz’oretta, poi dipende quanto una persona si vuole dilungare.
R:	No, però era per questa domanda dicevo. Va bene, guarda, la mia esperienza è sicuramente molto positiva; ovviamente bisogna considerare che io non avevo esperienze precedenti all’interno di Comitati di Etica e quindi sicuramente, col fatto che sia tutto nuovo, contribuisce molto al mio entusiasmo ecco, ho cercato di partecipare il più possibile. Ma al di là appunto di questo fatto qui vedo che, riunione dopo riunione, personalmente imparo moltissime cose, non solo proprio a livello di contenuti o di tematiche di cui nella maggior parte dei casi non mi ero mai occupato, ma anche imparo, credo che sto imparando questo modo di relazionarsi, questa dinamica di gruppo, che è molto interessante, che sta portando a condividere ecco delle esperienze, delle visioni completamente diverse, perché siamo così tanto eterogenei, siamo venuti, tanto veniamo da… abbiamo storie completamente diverse. Quindi sono molto contento in generale, ecco sì non ho oltre a questa soddisfazione non ho molto altro da aggiungere ecco.
D:	Okay. Allora io ti faccio adesso una serie di domande un po’ anche più specifiche sul servizio e ti chiedo qual è secondo te il ruolo del Comitato per l’Etica nella Clinica all’interno dell’azienda.
R:	Questa è la domanda? Scusa. Bene. Allora io penso che ecco il ruolo del Comitato per l’Etica sia cresciuto; all’inizio appunto siamo in modalità sperimentale, però almeno dall’esterno mi sembra di vedere che tutti o sempre più persone si siano rese conto dell’utilità di questo strumento e quindi è stata una grossa rivincita, anche proprio da un punto… da una prospettiva di bioetica, perché la vita del CEC in questo primo anno, giusto, anno e qualcosa?
D:	Siamo a venti mesi, più o meno.
R:	Dimostra che appunto l’etica era magari all’inizio prima un interesse di qualcuno, un qualcosa in più, e adesso invece sta diventando, anche tramite il CEC nell’azienda, un qualcosa di cui tutti possono avere bisogno. È un qualcosa che può sbrigliare molti nodi che per tanto tempo sono stati lasciati lì, quindi io penso che appunto il CEC inevitabilmente sarà sempre più forte, sempre più radicato e sempre più in una direzione sempre più professionalizzante ecco.
D:	E secondo te, in che modo questo tipo di servizio si differenzia da altri servizi che sono promossi dall’azienda? Qual è la sua specificità?
R:	La sua specificità è il fatto di riunire delle persone che hanno una formazione completamente differente e qui ovviamente ci sono anche all’interno dell’azienda equipe multidisciplinari, però così tanto multidisciplinari come il CEC non credo, anche perché ci sono io che sono un giurista e altri e soprattutto ci sono persone dall’esterno. Questo penso che sia importante, perché comunque contribuisce anche un po’ a rompere la visione aziendalistica, che spesso può influire in alcune decisioni, in alcune visioni. Quindi il valore aggiunto è appunto sicuramente la ricchezza di esperienze, di prospettive che i membri portano e che poi si riversa sulle decisioni prese, sulle attività, sui pareri adottati, eccetera.
D:	Poi ti chiedo, qual è secondo te l’obiettivo di questo Comitato per l’Etica nella Clinica e in che modo agisce, diciamo così, nel contesto locale?
R:	Da membro esterno l’obiettivo per cui è stato creato non saprei molto dire, quello che posso dire appunto come membro è che sicuramente adesso siamo in un periodo particolare, per tutta una serie di questioni che stanno affiorando, e il Comitato per l’Etica nella Clinica si trova al posto giusto nel momento giusto, perché può fungere da pungolo per tutta una serie di questioni, se non direttamente essere investito, vediamo poi cosa succede, di alcune competenze di tipo nuovo. Quindi credo che ci sia un gran bisogno di questo tipo di competenza, quello che offre il Comitato per l’Etica Clinica e il CEC è lì, al posto giusto per offrirla.
D:	Ti chiedo a che cosa ti riferisci nello specifico, cioè se ti va di approfondire un attimo a che cosa ti riferisci nello specifico rispetto a queste questioni nuove di cui hai accennato e per cui da quello che hai detto il CEC potrebbe, cioè secondo te, è al posto giusto nel momento giusto.
R:	Mi riferisco alle ultime vicende che emergono tra storie vere e anche sviluppi legislativi nel tema del fine-vita, che intreccia molto quello delle cure palliative. Stiamo vivendo un periodo molto complesso, in cui il nostro Paese, la nostra società da una parte sta cercando di avviare una riflessione un po’ più matura finalmente, rispetto al passato, anche dettata da una serie di eventi, da una serie di persone che hanno un po’ sfidato il nostro panorama, le nostre restrizioni, il nostro panorama giuridico. E quindi il CEC, anche in virtù della sentenza della Corte Costituzionale sul caso Antoniani-Cappato, può comunque aiutare ecco, se non direttamente forse indirettamente, se non proprio decidendo su alcune situazioni magari anche in maniera amministrativa, fungendo da supporto. Quindi penso che la nostra esperienza può davvero diventare utile per qualcuno.
D:	Okay, quindi come supporto dicevi te, okay. In che modo secondo te questo Comitato agisce proprio nel contesto locale? Forse non mi era molto chiaro che cosa ne pensavi te, cioè che cosa ne pensi, qual è stata la tua percezione?
R:	Ma questo devo dire, da esterno non saprei rispondere molto bene, perché so che in qualche modo il territorio è stato… è rappresentato… ci sono diversi… alcuni membri comunque si fanno carico di questa cosa qui. Però soprattutto essendo in modalità online è molto difficile dire questo.
D:	Okay. E adesso ti chiedo invece quali sono le motivazioni che ti hanno spinto personalmente a partecipare, a prendere parte a questo servizio.
R:	Devo essere sincero che ho sempre voluto fare questo tipo di esperienza; prima cosa perché sono una persona molto curiosa e mi sono sempre chiesto come avvengono questi processi decisionali, come funziona la soluzione di un conflitto, come avviene in concreto ecco, quando si tratta di interessi vitali, di salute o di cose davvero molto importanti. E questo appunto è, posso dire che così, nel piccolo ho realizzato un sogno, grazie al Comitato per l’Etica, perché davvero mi sento appunto di essere riuscito a toccare una materia grigia che prima mi sembrava totalmente inaccessibile ecco.
D:	Ecco, allora ti chiedo, un po’ l’hai anticipato, ma se vuoi aggiungere qualcosa, ti chiedo in che cosa consiste di preciso il tuo lavoro all’interno del Comitato per l’Etica nella Clinica.
R:	Il mio lavoro è quello di tutti gli altri, sono un membro, sono esterno, che lavoro a (redacted); io sono di formazione giurista e bioeticista, quindi cerco di dare il mio contributo anche e non solo ovviamente per le questioni giuridiche, perché poi a fare bioetica, come tu ben sai, vuol dire doversi e volersi occupare un po’ di tutto. Sono specializzato in nuove tecnologie, ancora questo tema non è molto venuto fuori, solo in un caso se non mi sbaglio, e un’altra grande area di mia competenza è la salute interculturale insomma, le immigrazioni. Penso che la pandemia ha molto bloccato questo tema, perché il Covid ha stravolto tutte le gerarchie, però in una città come Reggio Emilia, comunque in generale, in una Regione come l’Emilia-Romagna penso che anche questa questione del rapporto tra trattamenti, nuovi trattamenti e le opzioni culturali diciamo così, può essere in futuro molto sentite ecco, può portare a nuovi conflitti; quindi magari potrei, spero di poter essere di aiuto in qualche modo.
D:	L’altra domanda che ti faccio è che cosa ne pensi del valore di un servizio come quello offerto dal Comitato per l’Etica Clinica per il tuo lavoro.
R:	Per me è molto importante, molto importante perché posso restituire tutto quello che sto imparando, ed è una cosa che sto facendo proprio quest’anno, ho fatto, con un corso di bioetica, in cui cerco di trasmettere ai ragazzi anche facendo qualche esempio di conflitti, di questioni, di dilemmi ecco, etico-giuridici, quindi posso portare ecco questa esperienza, posso trasformarla in una maniera, in qualcosa di didattica, in qualcosa che possa essere da insegnamento per i miei studenti, che sono giuristi e quindi hanno moltissimo bisogno di sporcarsi le mani con la bioetica.
D:	E invece per i tuoi colleghi? Intesi anche per esempio nel CEC oppure in generale?
R:	Devo dire purtroppo che dove lavoro io sono l’unico, e ripeto di nuovo purtroppo, sono l’unico che fa bioetica; sicuramente se avessi dei colleghi che lavorano in quest’area sarebbe molto interessante anche per loro, però ecco non ho questa possibilità.
D:	Ti chiedo invece magari pensando ai colleghi del CEC, ti viene in mente qualcosa rispetto al valore che può avere questo servizio?
R:	Sicuramente come io sono molto entusiasta e credo, sono convinto che sto imparando tantissimo, anche per gli altri lo vedo anche un po’ sembra questa una cosa evidente, siamo tutti molto, come posso dire, partecipi di questa esperienza. Ovviamente c’è chi ha avuto o ha già tantissime esperienze in quest’ambito, avendo partecipato a diversi comitati, eccetera, o a processi anche più complicati, come chi ha fatto parte di poteri legislativi, eccetera, o assemblee. Però questa è un’esperienza specifica secondo me, perché appunto ci siamo noi, ed è adesso, in questa fase così delicata, quindi io vedo che comunque dalle persone con meno esperienza, come posso essere io, a quelle che ne hanno molte di più vedo una grande partecipazione, quindi è una cosa sicuramente positiva.
D:	Adesso la prossima domanda ricalca un po’ quella che facevo prima, quindi ti chiedo semplicemente se vuoi aggiungere… è riferita un po’ al contesto locale, e cioè se secondo te pensando alle attività che in questi mesi sono state svolte dal Comitato per l’Etica Clinica hai avuto modo di pensare e di capire, di cogliere la modalità diciamo con cui queste attività sono state integrate nel contesto locale.
R:	No, ripeto un po’, nel senso che veniamo da due anni in cui la nostra vita è stata digitalizzata completamente, quindi sicuramente questo ha avuto un ruolo, non solo per me, anche per l’integrazione nel senso del servizio. Immagino che in un periodo normale potrebbe essere totalmente differente, ci potrebbe essere molto più una conoscenza ecco mediata dal rapporto personale, piuttosto che appunto l’invio di un link al sito del Comitato, con un invito a vedere come funziona, eccetera. Cioè io penso che sia molto importante per un contesto locale ristabilire ecco il valore della relazione personale, perché alla fine quella che media sempre l’esperienza può portare almeno nella prima fase di affermazione del servizio a farlo crescere. Dopodiché io immagino che di qui a qualche anno il servizio, il CEC camminerà sulle proprie gambe; però ancora in questa prima fase, secondo me abbiamo perso l’opportunità di… si è persa un po’ l’opportunità di lavorare davvero nel contesto locale.
D:	Ti chiedo per capire, cioè tu ti riferisci a un’attività tra voi componenti del CEC o tra componenti del CEC e professionisti dell’azienda? Questa è cosa che non mi è molto chiara.
R:	La seconda che hai detto, tra il CEC e l’azienda; ovviamente anche tra di noi il fatto di stare sempre online, per carità ha un vantaggio assoluto. Penso che qualche occasione per incontrarsi di persona sarebbe comunque forse anche importante, ma semplicemente per il contatto personale non è mai come… e anche le relazioni tra di noi possono essere… possono portare ecco a fortificare alcune cose o a rivelarne delle altre. È semplicemente un altro tipo di esperienza; però quello a cui mi riferivo è soprattutto il rapporto tra il CEC come istituzione e l’azienda, l’ospedale, in cui sicuramente il fatto di essere quasi sempre o per quasi tutto online un po’ ha messo i bastoni tra le ruote secondo me. Poteva essere un po’ più semplice portare il CEC in tutta l’azienda, in tutte le ramificazioni, se non ci fosse stata la pandemia.
D:	Okay. Infatti l’altra domanda che si ricollega un po’ a questo, quindi ti chiedo semplicemente se hai voglia di aggiungere qualcosa e se ci sono stati, ci sono, ci sono stati dei limiti all’implementazione di questo servizio.
R:	Dei limiti non lo so, nel senso che a livello organizzativo, se ci sono dei limiti magari io da membro difficile che li possa cogliere. Non so se poi c’è una domanda su questo, magari se c’è un aspetto che magari io penso che si possa migliorare.
D:	Sì, c’è anche su questo ovviamente.
R:	C’è già una domanda?
D:	Sì, ma sentiti libero quello che desideri.
R:	No dico, ma la dico adesso o aspetto la domanda?
D:	No, dillo pure adesso.
R:	Guarda, secondo me è una cosa che, non so, sicuramente voi c’avete già pensato, eccetera, però quello che io ho percepito nel momento in cui le richieste aumentano e tra l’altro aumentano anche mi è sembrato a volte anche un po’ di complessità, secondo me è lavorare meglio sulle richieste, in maniera un po’ più… cioè le richieste dovrebbero essere un po’ più filtrate, un po’ più elaborate, non filtrate, devono essere maggiormente elaborate. Dovrebbero venire già un po’ più strutturate, secondo me, per questo facilita il vostro… non so se qualcuno ha mai pensato magari a predisporre un format, in cui uno già molto strutturato in cui questo facilita chi fa la richiesta, perché deve solamente compilare, poi alla fine magari c’è uno spazio più descrittivo, però questo potrebbe anche servire a noi a orientarci meglio, perché a volte, quello che ho notato io, almeno per me, sono dovuto andare a cercare le informazioni; se invece ci fosse un formulario in cui dici chi fa richiesta, qual è il soggetto, qual è il tema, qual è il contesto, che ne so, (? 22:11) un po’ più strutturato secondo me sarebbe un passo in avanti, anche per uscire insomma quando si vorrà uscire dalla modalità sperimentale.
D:	Okay, questo più che altro è un processo di informazione e comunicazione nel passaggio delle richieste.
R:	Sì, di riorganizzazione di quella fase lì, perché può… è successo sicuramente una o due volte, che qualcuno non aveva capito bene di che cosa si trattava, ed è successo magari anche a me di non essere totalmente sicuro, che magari avrei voluto chiedere qualcosa in più. Per questo dico sono due possibili problemi (? 23:01-23:08) si accavallano, uno è (? 23:10-23:13), mi senti?
D:	Sono arrivata a due possibili problemi che si accavallano.
R:	Okay, te lo ripeto. Uno è che chi fa la richiesta non spieghi benissimo quello che… perché anche spiegare un caso, soprattutto se è complesso e ha una storia, ci sono diverse problematiche, può non essere semplicissimo; in fin dei conti se te lo spiega un’infermiera o un dottore loro non hanno mica fatto scienze della comunicazione, quindi non è detto che siano preparati per fare questo. E poi il problema è portare questo messaggio nella forma in cui l’hanno dato a te o a chicchessia ad altre persone; quindi ci sono due mediazioni e il rischio secondo me è che qualcosa vada perso in questi due passaggi, quindi adottare una struttura un po’ più ferma ci potrebbe consentire di avere un po’ più il quadro chiaro a volte, ma a volte non ce n’è bisogno perché sono molto semplici.
D:	Questa te la chiedo ma a scopo più che altro personale diciamo; per esempio un altro elemento che viene molto dibattuto proprio su questo punto qui è anche la presenza della persona stessa che fa la richiesta all’interno delle riunioni del Comitato, quindi mi veniva in mente…
R:	Cioè qualcuno vorrebbe includere il richiedente?
D:	Sì, per esempio in letteratura è uno degli argomenti che proprio sui Comitato per l’Etica nella Clinica sta emergendo. Quindi niente, mi era venuta in mente questa riflessione rispetto appunto al discorso che facevi te.
R:	Io sono un po’ scettico su queste cose qui, nel senso che sicuramente compromette molto la dinamica discorsiva, perché poi si può parlare di qualcosa in cui magari il richiedente ha sbagliato o insomma non ha fatto benissimo o poteva fare diversamente; quindi questo secondo me toglie molta libertà di fatto alla discussione. Mi piace invece molto la dinamica che stiamo adottando noi, ovvero il fatto di discutere e poi casomai invitare il richiedente a una seconda discussione. Quindi separare le due cose mi sembrava più… ovviamente è oneroso, per un Comitato che abbia un certo numero di richieste non è facilmente attuabile, perché moltiplica di fatto le riunioni, ma questo insomma non c’entra molto; un conto è cos’è l’optimum, l’ideale, e un conto è che cosa ti serve per realizzarlo. Quindi io insomma spero che il CEC sia finanziato e che riceva il supporto massimo da parte dell’azienda, anche per aiutare voi a fare quel salto di qualità che è richiesto.
D:	Ti chiedo invece se secondo te ci sono state, ci sono delle potenzialità o quali sono delle potenzialità di questo servizio.
R:	Di questo in particolare o in generale…
D:	No, di questo.
R:	Mi sembra appunto che il CEC sia un po’, da quello che ho capito, sta facendo un po’ da traino per tutta la Regione probabilmente, si sta ponendo come un esempio virtuoso. Quindi da una parte ecco, potrebbero arrivare richieste anche che non sono strettamente intese così del territorio di Reggio Emilia e dall’altro, cosa che mi sembra sia già accaduta, se non all’interno della Regione però fuori Regione, il CEC si sta ponendo come un modello, questa è una cosa, è già un grandissimo risultato secondo me.
D:	Adesso ti faccio le ultime due domande, poi abbiamo concluso. Ti chiedo se alla luce di quanto fatto in questi mesi, come valuti il servizio che è stato implementato.
R:	Numericamente intendi o in generale?
D:	Una valutazione in generale.
R:	Io lo valuto molto positivamente ecco, la mia valutazione è assolutamente positiva e molto positiva ecco.
D:	L’ultima domanda che ti volevo fare è se secondo te ci sono delle modalità con cui può essere migliorato, che è la domanda che abbiamo visto prima.
R:	Sì, era questo secondo me, il rafforzare il passaggio dalla richiesta alla formulazione del caso, in modo da rendere più semplice appunto il compito di cui poi ci deve lavorare. Questo qui, di renderlo un po’ più strutturato. E poi secondo me questa attività, soprattutto pensando al lavoro che stai facendo tu, potrebbe diventare facilmente a parte rendicontabile, ma anche proprio trasformabile in un altro risultato di ricerca; perché se tu hai fatto tutto tramite in una maniera più strutturata, dopo trasformarlo in un report, in un qualcosa, per esempio fare ricerca sulle richieste stesse diventa possibile. Invece tenendole un po’ così si perde un po’ qualcosa, secondo me; però ripeto, ovviamente siamo al primo anno e qualcosa, io sono sempre, ho questa forma mentis, non appena raggiungo un obiettivo già sto pensando all’altro, quindi prendilo, prendetelo come una mia ossessione, il fatto di voler migliorare sempre.
D:	No, ma ci mancherebbe, anzi, è un pallino di molti, un pallino che spinge poi ad andare a fare le cose sempre meglio alla fine.
R:	Sì, infatti.
D:	Ma difatti anche queste domande ci servono sia per capire come è andata, però servono proprio anche per capire in maniera molto concreta, come il suggerimento che ci hai dato tu adesso, però anche in generale quale deve essere, qual è lo sviluppo per migliorare sto servizio.
R:	Ma l’azienda che intenzioni ha a proposito, cioè ci vuole mettere, ci vuole investire o…? Penso di sì. O c’è qualche…
D:	Con l’azienda adesso cioè c'è ancora in ballo sto discorso qua del tema sul suicidio medicalmente assistito, che è ancora altamente in discussione. Io anche parlando – questo sarà fuori dall’intervista, ma parlando anche con (redacted) le cose non si sono mosse in maniera ufficiale rispetto agli ultimi aggiornamenti che ci siamo dati, quindi sicuramente avere questi dati, questa valutazione saranno dei dati importanti da dare alla Direzione per capire qual è, che cosa ha fatto, che cosa ha fatto effettivamente.
R:	Però ancora non si sa; voglio dire, usciti dalla fase sperimentale cosa succederà? O sarà prorogata la fase sperimentale.
D:	Sì, in questo sì, anche perché ha una durata di tre anni, da Regolamento, ha una durata di tre anni, quindi il primo anno…
R:	Ma tre anni in modalità sperimentale?
D:	No, era tre anni da Regolamento, il primo anno era sperimentale; poi ecco, per tutta una serie di discorsi, mi viene in mente anche di sostegno dal punto di vista economico, eccetera, lì ci sono degli altri discorsi da fare; nel senso che con il supporto della… cioè se c’è un supporto della Regione per quel discorso per esempio del suicidio medicalmente assistito si aprono delle altre partite.
R:	Sì, se passiamo a doverci occupare di quelle case lì c’è bisogno di un cambio molto significativo.
D:	Sì, anche a livello di assicurazione, come si diceva in una delle ultime riunioni, perché poi ci sono delle decisioni non indifferenti da prendere. Però al momento la situazione è ancora in stallo.
R:	Va bene.
D:	Comunque una parte di queste interviste è fatta anche tipo ai dirigenti, cioè alla Direzione, quindi dovrò intervistare anche il Direttore Generale, se ci riesco, se non mi manda a quel paese, il Direttore Sanitario, quindi…
R:	Serve un po’ da stimolo, da pungolo.
D:	Sarà un modo per testare il terreno in maniera più solida. Vediamo, secondo me manco me la legge la mail, comunque ci proviamo dai, ci proviamo.
R:	Va bene. Questo è concluso, sì?
D:	Ė conclusa, l’ultima domanda che ti faccio è se rispetto a quello di cui abbiamo parlato in questa intervista vuoi aggiungere qualcos’altro su questo tema, sulla tua esperienza, sul servizio, se ti è venuto in mente qualcos’altro che vuoi condividere.
R:	No, l’unica cosa che vorrei condividere è questa; in realtà la volevo dire a (redacted), però adesso butto la pietra. No, non gliel’ho detto perché poi ci sono stati periodi davvero complicati, sia per me che per lei. A me piacerebbe tanto far partecipare a una riunione, magari a una riunione un po’ più tranquilla, qualche studente; perché penso che gli studenti miei per esempio, che io ho una clinica legale in bioetica, non so se conosci questo modello, è un insegnamento insomma, si chiama clinico perché viene appunto dall’ambito medico, ma in realtà non c’è niente di clinico, in senso medico. Portiamo gli studenti insomma appunto a cercare di sporcarsi le mani il più possibile, quindi gli diamo dei casi da risolvere, gli diamo un po’… cerchiamo di farli immergere nella pratica; e per me, che la mia clinica appunto è in bioetica, sarebbe bello per esempio se gli studenti potessero assistere a una riunione, così potrebbero capire come l’etica davvero interseca il diritto, cioè come… in che ruolo si pongono queste due aree, eccetera. Sarebbe molto interessante per loro e contribuirebbe secondo me anche che il CEC possa perseguire una funzione così, didattica in un certo senso. Cioè sarebbe non solo uno strumento pratico, ma anche formativo ecco, per qualcuno, visto che comunque la formazione è nella mission statement dell’unità più che del CEC, però ma ne parlerò con (redacted), non so se, non so come… te che ne pensi di questa cosa qui?
D:	Potrebbe essere sicuramente utile, per avere uno sguardo a 360 gradi su quello che effettivamente fa un Comitato, su cosa significa occuparsi di etica in maniera pratica, questo sì.
R:	Per loro sarebbe un’esperienza fantastica; magari ecco, in una delle riunioni in cui già si sa che non ci sono cose molto personali, che ne so; ma tanto poi alla fine sono tutte, parliamo sempre in maniera anonima, non è che… siamo già abituati ecco, non è che parliamo di Tizio e Caio, parliamo più in astratto, noi. Quindi non sarebbe un problema sotto questo punto di vista secondo me, però mi rendo conto che anche spiegarlo agli altri che non sono accademici può essere complicato.
D:	Ci riflettiamo, intanto grazie per la…
R:	Ci riflettiamo.
D:	No, vediamo se è possibile farlo, perché no? Anche secondo me è un’ottima idea per coinvolgere, per far arrivare il più possibile questi messaggi, perché alla fine poi l’obiettivo è quello, cioè avere le lampadine accese, le lampadine che si accendono su questi temi.
R:	Vediamo, se piace sarebbe…
Fine trascrizione 00:37:40
```
