# Supplementary material for: How to implement a clinical ethics committee in an oncological research hospital: Qualitative results from a process evaluation study using normalization process theory (EVACEC)
Source: PLoS One. 2025 May 6;20(5):e0318870. doi: 10.1371/journal.pone.0318870 (PMC12054913; doi:10.1371/journal.pone.0318870)
Supplement: S5 File — Interviews transcripts_raw data: this folder contains all the raw data used in this study, the interview transcripts (in original Italian). (ZIP) [file pone.0318870.s005.zip › S5_Interviews transcripts_raw data/14CM.doc]

```
D: 	Allora, questa sarà un’intervista magari anche un pochino ripetitiva, può essere appunto che ci siano delle domande ecco che si ripetono un po’, però questo è dovuto anche al tipo di framework, tipo di teoria che stiamo seguendo proprio per questo progetto. Quindi può essere appunto che ci siano dei temi su cui… che magari sono già emersi nella risposta di una domanda, quindi semplicemente ti chiederò se ti va magari di aggiungere qualcosa o se c’è qualcosa che…
R:	Perfetto.
D:	Okay. Allora, la prima domanda che ti faccio… ecco, un’altra cosa, ci saranno domande un po’ più personali, rispetto a quella che è la tua esperienza, la tua esperienza col servizio e domande invece più specifiche, proprio su come valuti tu questo servizio. Quindi la prima domanda che ti pongo è se mi vuoi raccontare qual è la tua esperienza nell’ambito della bioetica.
R:	In generale dici?
D:	Sì, sì, in generale, in questo momento.
R:	La mia esperienza in ambito della bioetica è zero.
D:	Okay.
R:	Nel senso che prima di questo Comitato sapevo che esisteva, cioè so che è un campo insomma, un’area, sapevo più o meno che cos’era e per formazione mia scolastica, cioè nel senso per il mio curriculum di studi, io sono laureata in filosofia, in particolare in filosofia morale, quindi comunque diciamo che non sono… non che io abbia studiato la bioetica per niente, ma insomma comunque riesco a comprendere che cos’è, però è proprio un riuscire a comprendere di cosa stiamo parlando. Quindi è un’area che magari mi poteva… cioè anzi mi interessa e per me è interessante, ma perché è una formazione appunto molto umanistica e quindi ovviamente… poi lavoro nel sociale praticamente da sempre, quindi comunque sono declinazioni che in qualche modo in particolare l’etica ecco, però l’etica comunque è in generale un tema per cui insomma io in tanti momenti ne ho parlato e ho approfondito questi temi, legati però ad altre cose, quindi ho approfondito un sacco di volte i temi etici, non bioetici ovviamente. Però quando ho detto di sì a questo Comitato l’ho fatto sia per un grande interesse mio personale e sia perché ritenevo che la mia esperienza potesse arricchirsi diciamo con un (? 03:04) di percorso di questo tipo, ecco. Però no, io di bioetica anzi non ne so nulla e quello che ha fatto questo Comitato a me personalmente, al di là degli esiti vari, eccetera, a me personalmente mi ha fatto venire molta voglia di approfondire queste tematiche e più che approfondire proprio formare, perché poi adesso non so se ci saranno altre domande simili, però comunque lo condividevo anche l’altro giorno con (redacted), in una telefonata che le ho fatto, e penso che al di là dell’andare di pancia, ci sia proprio anche una necessità mia sicuramente ecco di formarsi per centrare bene che cosa sia e che cosa non sia soprattutto; perché… in maniera anche da essere più utili in questo Comitato ecco.
D:	Scusa, ti chiedo per mettere…
R:	Io di bioetica però non ne sapevo niente.
D:	Okay, ti chiedo rispetto a quest’ultimo punto se mi vuoi… se puoi precisarmi che cosa intendi per capire meglio che cosa sia e che cosa non sia, che è quello che hai appena detto; cioè la bioetica o il Comitato per l’Etica Clinica o… non ho ben capito. […]
R:	Allora, cosa sia e cosa non sia, nel senso che cioè non è tanto che cosa sia o non sia la bioetica, secondo me c’è un po’ la necessità di approfondire meglio quali compiti ha questo Comitato, sicuramente e cosa sia, cosa non sia, non so; adesso vorrei spiegarmi bene, ma non so se…
D:	No, no, vai pure.
R:	Ti faccio, mi provo a spiegare con un esempio, che ho fatto già a (redacted); l’altro giorno, cioè negli ultimi incontri, tutto il tema del (redacted), eccetera eccetera, è stata forse la prima volta in cui io ho recepito veramente una grande concretezza rispetto al ruolo del Comitato. E in tutto l’iter precedente, poi non è successo nell’ultimo incontro, io ne sono stata molto contenta, ma in tutto l’iter precedente si è fatto tantissimo riferimento alle leggi che normano e quindi… io a un certo punto, non l’ho detto perché avevo un po’ un senso di inadeguatezza, a un certo punto mi veniva da dire “ma va bene le leggi che peraltro io non conosco neanche approfonditamente e che magari mi andrò anche a studiare”, ma a un certo punto mi sono detta “ma noi qua stiamo continuando a dire cosa dice la legge, ma io credo che cosa dica…” cioè, che non siano, cioè che il nostro problema non sia andare a guardare cosa dice la legge. Cioè mi sembrava che fosse più legato a temi più di tipo esistenziale, a temi di scelte, a temi più legati alla coscienza. Per quanto ne so io l’etica non è un qualcosa di totalmente oggettivo e quindi la legge è una cosa oggettiva; cioè al di là magari delle interpretazioni, però è già più oggettiva, la legge mi sembra di capire che nel gruppo, a parte me, la conoscano più o meno tutti. Ma non mi sembrava, cioè mi sembra che a volte si stia molto sulla legge e meno sui discorsi che invece magari si sono riusciti a centrare di più appunto con la presenza della persona interessata e lì in quel caso, ad esempio, insomma, le domande erano anche abbastanza centrate e un po’ anche per la disponibilità di questa persona si è stato anche su un discorso che secondo me è stato molto più utile ecco di quelli magari fatti prima da noi, che erano “la legge dice questo” e quindi insomma… in questo senso ecco, mi piacerebbe approfondire davvero quanto… approfondire proprio questo tema, per capire esattamente che cos’è, che cos’è la bioetica, da quali principi si può partire, quali tipi di visione ci sono. Io credo che sia una cosa per me utile, per il gruppo non lo so ecco, sembrano tutti abbastanza ferrati ecco, sul campo, però a me sembra che ci sia molto tecnicismo… […] ci sia molto tecnicismo e sia… […]
D:	Se mi vuoi raccontare quali sono, qual è la tua esperienza quindi con il Comitato per l’Etica nella Clinica.
R:	Allora, la mia esperienza, dunque… diciamo che se devo pensare a una parola in questo momento ecco mi viene in mente un po’ il termine inadeguatezza, una parola che mi rappresenta dentro questo gruppo, mi viene in mente inadeguatezza, nel senso che io mi sento un po’ in quelle situazioni di non parità, perché c’è mi sembra di vedere anche un utilizzo di terminologie di non so se sia alta o comunque appunto molto tecnica, molto… così e io invece mi sento un po’ inadeguata in questo senso, perché gli interventi che faccio sono sempre… intanto ne faccio pochissimi, perché proprio dico adesso dico una roba così questi qua pensano che io sia scema. Però insomma, sì è una sensazione ecco, per me la complicazione è proprio quella di sentirmi un po’ inadeguata e che le competenze non siano pari. Fatico un po’ a portare le mie di esperienze e di competenze e quindi cioè è un’esperienza un po’ difficoltosa per me; continuo a trovarci comunque una cosa… cioè continuo a trovare una cosa molto interessante, ci credo molto, perché il fatto che io mi senta inadeguata non vuol dire che io ritenga che questo gruppo non sia utile, anzi, io credo che sia utilissimo e soprattutto forse in questi tempi insomma. Anche solo per… anche perché i casi si stanno moltiplicando, anche perché la cultura sta cambiando, anche perché ci sono proprio dei cambiamenti davvero appunto culturali, che secondo me vanno seguiti, accompagnati, monitorati e quindi io credo che questo sia un lavoro assolutamente utile. Quindi l’utilità per me è altissima, mi chiedo, mi dico chissà quanti casi non arrivano lì perché magari non si conosce che c’è il Comitato, non si sa che… penso che possa essere anche sfruttato ancora di più, magari dalla classe medica, perché credo che a volte in realtà noi che non siamo medici e magari non ce ne accorgiamo ma i medici vivano comunque dei grossi dissidi. Cioè facendo questo tipo di lavoro mi metto nei loro panni e dico chissà quali dissidi, chissà quali domande ecco, si fanno queste persone che per noi sono quelli che devono dare le risposte. Cioè per il cittadino il medico è quello che dà risposte. Lo diceva proprio l’altro giorno Sogliano, giustamente, diceva “da me uno da me si attende una risposta”; per il cittadino il medico è uno che non ti dice “ma potrebbe essere così, ma potrebbe essere cosà o potrebbe essere così”; cioè io cittadino non me ne faccio niente di quella roba lì. Io stessa per la mia esperienza personale, che cinque anni fa mi hanno trovato un tumore nei polmoni ho avuto un medico che mi ha detto “ma potrebbe essere così, però potrebbe essere cosà, però potrebbe essere così”, io l’avrei ammazzato. E d’altra parte, voglio dire, i medici sono quelli che danno le risposte e però io suppongo che a livello personale, ma forse anche di competenze tecniche insomma, voglio dire, perché poi la medicina è una scienza esatta fino a un certo punto e quindi mi dico chissà quali dissidi loro appunto vivono. Ne ho avuto la prova l’altro giorno appunto, con il caso che abbiamo trattato. Questa intervista, se fosse stata prima di questo caso qua, probabilmente sarebbe andata un po’ diversamente. Fatta in questo momento mi dà un po’ più spunti di riflessione insomma e quindi… ma tanto che ti dico ho chiamato Ludovica l’altro giorno proprio perché a fronte di questa roba a me sono venute in mente un sacco di cose che… però perché siamo entrati molto nel caso, perché c’erano le parti, non c’era la persona ovviamente, ma c’era il medico, c’erano… quindi per me è stato molto… l’esperienza, ti dico, stando alla domanda è di grande inadeguatezza, sicuramente di bisogno di formazione e sicuramente un’esperienza abbastanza faticosa e vorrei avere il tempo ogni volta che facciamo gli incontri magari per leggere tutte quelle cose che arrivano in qualche modo, ci sono tanti contributi che le persone poi vedo che le mandano. Però sicuramente è un’esperienza molto… e comunque un’esperienza positiva; cioè, al di là del fatto che le immagini che io sto usando per seguire che questa cosa non siano particolarmente positive, però è comunque per me, in questo momento, un’esperienza positiva. Forse anche lì, rappresento i cittadini che (? 15:10) dare appunto questa… c’è un po’ questa diversità dei ruoli, e forse il ruolo del cittadino andrebbe un po’ più aiutato. Cioè, io che come lavoro conduco gruppi di lavoro, che metto in rete, facilito, eccetera, mi dico che a volte il ruolo del cittadino o comunque di quelli che si sentono un pochino più… meno inseriti forse in quel mondo, forse andrebbe un pochino più facilitato, anche se ti dico che in certi momenti mi dico certo che se uno mi dicesse “ma tu cosa ne pensi?” Perché magari io non (? 15:57) a dirlo io direi che penso che sono inadeguata e quindi insomma la paura è sempre quella di andare un po’ di pancia, di… Poi mi metto tante volte nei panni… è chiaro che un cittadino poi si mette… poi forse anche con la mia esperienza mi metto tante volte nei panni proprio del paziente e cioè, mi dico sempre quanto hanno ragione a fare queste richieste, mi dico sempre che li capisco, perché io credo che farei la stessa cosa e quindi insomma è anche un’esperienza personale molto forte; un percorso personale che insomma, anzi, vale la pena di approfondire insomma. Però è comunque un’esperienza che malgrado tutto per me è in ogni caso positiva. Sicuramente potrebbe essere un po’ facilitata, sia dalla formazione sia appunto da una facilitazione della tenuta del… non so, non della tenuta insomma…
D:	Della conduzione?
R:	…dell’equilibrio di gruppo ecco, non so come dire.
D:	Dell’equilibrio.
R:	O forse della composizione, forse anche della composizione, anche se poi mi chiedi chi ci vorrebbe non saprei…
D:	No, intendevo conduzione.
R:	Sì, anche conduzione. Deve essere un po’ più facilitata, però capisco anche molto ad esempio (redacted); cioè, c’è anche una grande, come posso dire, attenzione a non far sentir troppo… cioè, a lasciare una grande libertà di espressione, eccetera. Quindi la capisco bene, quindi magari se parli con un medico, il medico ti dice perfetto, perché poi magari loro si sentono appunto più adeguati e magari riescono a parlare di più, ecco.
D:	Ho capito, okay.
R:	Però non è solo una questione di conduzione, penso che sia una questione caratteriale mia, sicuramente, e forse anche un po’ di composizione, che magari… se di cittadini ce ne fossero due, non lo so, cittadini insomma o ruoli che non siano strettamente legati alla classe medica, a quell’ambito lì; perché c’è molto confronto su cose che…
D:	Okay. Va bene.
R:	Cioè persone che non siano medici o giuristi, ecco; perché attualmente lì ci sono medici o giuristi e quindi chiaramente… magari uno in più potrebbe servire, perché proprio c’è uno che ci (? 18:55) c’è il resto del mondo e così. Quindi per il cittadino è abbastanza, penso che sia un po’ complessa questa cosa.
D:	Ho capito. Okay, allora adesso ti faccio una serie di domande invece più specifiche sul servizio e ti chiedo qual è, secondo te, rispetto a quello che hai capito, rispetto a quello che pensi, il ruolo del Comitato per l’Etica nella Clinica all’interno dell’azienda.
R:	Il ruolo è un ruolo secondo me è un ruolo di supporto, mi viene in mente supporto; quindi è un ruolo di supporto, è un servizio comunque, secondo me è un servizio, un servizio importante anche per i cittadini intesi come pazienti, intesi come familiari. Credo che non ci siano familiari o pazienti che sappiano che c’è questo Comitato, credo che ce ne siano veramente pochi; potrebbe essere utile che le persone lo sapessero, anche per interpellarlo di più probabilmente e anche per capire che c’è una parte, che noi immaginiamo che non si faccia e invece si fa. Cioè di valutazione che non è solamente medica, diciamo una valutazione di tipo diverso. E quindi penso che sia un ruolo di supporto appunto anche per i medici, l’ho visto molto come ruolo di supporto per i medici e se ha un ruolo che abbia un ruolo molto importante, secondo me, di cambiamento culturale, nel cambiamento culturale penso che sia molto, molto importante, perché si ha un cambiamento culturale insomma; insomma anni fa non se ne poteva neanche parlare di questa roba, adesso almeno si riesce e parlarne ecco. E quindi è un ruolo appunto di supporto, un ruolo di cambiamento culturale, un ruolo che secondo me porta alla luce questioni che secondo me vanno portate alla luce, di cui si deve parlare, perché se non se ne parla poi funziona che appunto si sta solo alle leggi, le leggi poi se sono scritte male… come, cioè (? 21:34) questo caso, che sto cercando di capire bene, ma io non è che… però anch’io da cittadina, ripeto, direi cioè (? 21:43) basta che finisca questa (? 21:44), poi a me ne me frega di quello che mi succede dopo, però oggettivamente… Però vedi che c’è, cioè ci sono delle grandi differenze e io sono evidentemente una cittadina mal informata, perché contribuisco a mal informare, (? 22:01) quindi insomma. Quindi sia un ruolo culturale, di supporto, del portare alla luce le questioni, un ruolo consultivo, consultivo perché ovviamente è così, un luogo di confronto, forse anche per i medici tra loro, chissà, io non so se abbiano luoghi magari sì, magari no, abbiano un luogo di confronto proprio su queste tematiche; in un’equipe, secondo me, un luogo di confronto su queste tematiche, che non sia “cosa vedi tu nella lastra”, “cosa vedo io nella TAC” e “cosa vedi tu che sei un radiologo, cosa vedo io che è così, che forma è, di che è così”; ecco, secondo su questa cosa… perché i medici hanno anche secondo me bisogno anche di essere un po’, come posso dire, di avere il loro lato umano tutelato ecco. Quindi secondo me il medico col lato umano molto tutelato è comunque un medico più forte anche nei confronti del paziente, al di là delle sue competenze che devono esserci. Partendo dal presupposto che uno sia un medico capace, però un medico con un lato umano più tutelato secondo me è anche un po’ più chiaro, più… cioè, chiarito in certi momenti, penso che possa essere anche un aiuto per un cittadino.
D:	Okay. Quindi ti chiedo in che modo, secondo te, questo servizio è diverso da altri servizi promossi dall’azienda, qual è un po’ la sua specificità?
R:	La specificità mi sembra che sia quella di… magari non conosco tutti i servizi dell’azienda, però mi sembra che sia quella di stare su altri temi, cioè su temi che di solito pochi trattano; ecco, secondo me questa è una specificità molto… e che di solito si tende un po’ a negare e quindi penso che sia forse sicuramente una specificità. Istintivamente mi viene da dire che sia questa insomma e poi è comunque un servizio, cioè la specificità sta anche secondo me molto nelle innovazioni; cioè è un servizio che io trovo molto innovativo, poi magari forse è una storia… anche lì, da ignorante, perché poi magari è una storia lunghissima che io non so, però io prima di essere interpellata, io che lavoro nel sociale, che comunque ho avuto per mia sfortuna insomma una serie di esperienze come paziente, come familiare, come… così, molto forti, molto pesanti, molto… però questa roba io non sapevo che ci fosse e penso che sia un servizio molto innovativo insomma. Quindi credo che la specificità sia quella.
D:	Quindi, prima abbiamo visto il ruolo, poi voglio chiederti se hai qualcosa da aggiungere magari sull’obiettivo di questo servizio e di come agisce nel contesto locale.
R:	Allora, oddio, a me più che dirti che mi sembra che l’obiettivo sia un obiettivo di avere una… come posso dire, uno sguardo e riuscire ad avere uno sguardo esterno ma diverso da quello che solitamente probabilmente si ha in queste pratiche e mi viene in mente questo insomma, uno sguardo, una vera e propria consulenza; cioè consultivo nel senso che cioè insieme si prova ad arrivare non dico a una risposta, però comunque a dare ulteriori spunti di riflessione a chi poi alla fine deve decidere. Quindi insomma penso che nell’ambito della necessità di decidere avendo davanti più punti di vista, in maniera da non considerare solamente proprio il proprio, penso che abbia questo come obiettivo insomma. Poi ce ne saranno mille altri di ufficiali, però quello che…
D:	La tua idea, la tua percezione, sì, okay. Allora, prima me l’avevi già anticipato, ti chiedo solamente se hai magari qualcosa da aggiungere rispetto a quest’altra domanda che ti faccio, ed è in che cosa consiste il tuo lavoro in questo servizio.
R:	Allora, il mio obiettivo… dunque, il mio ruolo; allora, diciamo che secondo me se consideriamo anche che penso che l’obiettivo del Comitato sia anche quello un po’ di… un po’ tra virgolette, non so come dirtelo, mi viene da dire così, ma sarà…
D:	Sì, vai…
R:	…un po’ umanizzare…
D:	…proprio sentiti molto, molto libera di esprimere… cioè non aver…
R:	Sì, è che vorrei usare dei termini che mi spiegano, però… allora, diciamo che se considero che tra gli obiettivi di questo Comitato c’è anche quello un po’ di umanizzazione, cioè di umanizzazione della pratica clinica e di forse anche aumentare, cioè migliorare più che aumentare la qualità di un servizio sanitario, perché siamo dentro l’azienda sanitaria, quindi comunque quello è; quindi attraverso appunto l’umanizzazione, ma anche proprio aumentando la qualità percepita delle persone, io mi sento quella che porta il punto di vista di chi poi queste decisioni potrebbe subirle. Cioè queste… cioè di chi ci si potrebbe trovare, perché è chiaro che anche un medico ci si può trovare in questa situazione, però il medico ha sue competenze, ha sue… ha un suo background che potrebbe forse non dico aiutarlo, ma comunque gli dà stimoli ecco diversi. Invece secondo me il ruolo del cittadino è quello di ogni tanto alzare la mano e di dire “attenzione che…” cioè se uno è lì, sdraiato sul letto e voi state decidendo se fare una cosa o meno, cioè porta proprio la… secondo me è forse anche un ruolo di tutela dei diritti, cioè lo collego molto proprio alla tutela del diritto, diritto a decidere. Poi io sono cattolica e praticante, ma io su queste cose ci credo moltissimo, cioè nel senso che sono convita che però ci sia un diritto quantomeno alla non sofferenza ecco; poi di decidere della propria vita sì, da cattolica ti dico in realtà non è un diritto che mi arrogo, però di puntare alla minor sofferenza fisica e psichica e proprio, come posso dire, emotiva ecco, di puntare a quello sì, perché va bene tutto insomma, si dice anche chi è morto in croce non è che ce ne siano stati tantissimi. Quindi insomma eviterei di essere io quella, cioè nel senso che l’hanno già fatto, sono già a posto. No, nel senso che… in questo senso, capito? Cioè mi interessa portarti anche la… delle volte anch’io mi pongo il problema, io da cattolica perché sono qua che dico e così, perché dovrei dire ragazzi, finché uno è lì decide… in realtà non è quello che penso, perché quando io dovessi decidere in una situazione del genere sono abbastanza… e sulle DAT, ad esempio, perché ad esempio (? 30:42), un’altra occasione di approfondimento e io sono tra quelle che adesso andrebbe, cioè adesso che conosco il servizio andrò a fare questa roba, ma penso che, voglio dire, il mio ruolo sia proprio quello di dire insomma, c’è un diritto a decidere quantomeno il livello di sofferenza sopportabile, ecco; c’è un diritto di avere una qualità di assistenza buona o insomma il più elevata possibile, c’è un diritto di essere trattati umanamente e finché noi non diamo questo diritto a essere trattati umanamente anche ai medici, perché delle volte mi sembra che non sia dato nemmeno a loro questo diritto, di essere umani. Il Covid credo che l’abbia fatto emergere in una maniera… ma penso che fosse così anche prima, cioè non è che la sofferenza non ci fosse prima, dentro agli ospedali, c’era anche prima. Per cui comunque penso che il mio ruolo sia quello ecco di ascoltare la voce del cittadino, cioè di chi potrebbe essere in quella situazione al di là di tutte le cose che appunto dice la legge, dice la procedura, dice il dosaggio, dice questo, dice quell’altro. Cioè poi insomma, ieri, no, ieri l’altro, quando è stato l’incontro, io mi immaginavo, cioè avevo questa immagine di questa donna persa in mille questioni di dosaggi, meno dosaggi, questa stava semplicemente male; cioè era… lo so che un medico vede tante altre cose, però la persona vede che sta male, cioè era talmente chiaro questa… cioè più che dirlo, più che scriverlo, più che far così non credo che avrebbe potuto fare insomma, quindi…
D:	Sì, chiaro. Va bene. Poi, abbiamo ancora un po’ di domande, io non so se hai ancora del tempo, se no…
R:	No, ce la faccio.
D:	Okay, va bene. Allora, l’altra domanda che ti faccio e anche qui ti chiedo se hai da aggiungere qualcosa, è che cosa ne pensi del valore di un servizio come questo per il tuo lavoro e per quello degli operatori sanitari.
R:	Allora, parto dagli operatori sanitari, per me è un valore altissimo; lo deduco anche dal fatto che, credo, le persone che sono dentro al Comitato e di conseguenza presumibilmente anche quelle che verranno poi informate di queste… cioè a cascata, teoricamente uno che è dentro un Comitato poi potrebbe anche informare gli altri operatori sanitari, eccetera. Allora, credo che queste persone, che sono persone tutte molto impegnate, cioè parliamo di primari, parliamo di responsabili di… eccetera, io però li vedo sempre molto presenti e sul pezzo, eppure sono persone che, voglio dire, non so, dico (redacted) perché mi ricordo che una volta, anni fa, una riunione con (redacted) non riusciva ad arrivare in fondo alla riunione perché lui veniva chiamato ogni quattro secondi e così. Quindi sono persone estremamente… dico (redacted) ma perché ho visto lui, (? 34:18). Allora, io penso che il fatto che loro ci siano proprio dentro a questa roba e che stiano comunque… stiano concentrati, cioè non è gente che sta facendo altro, io credo che tante riunioni che facciamo noi c’è gente è evidente che sta facendo altre cose. Invece stanno lì, stanno sul pezzo; ecco, secondo me è una prova del fatto che secondo me questo Comitato ha un valore importante, cioè ha un significato importante e un peso importante. Evidentemente c’è un vuoto lì, cioè nel senso che se… evidentemente cioè c’è un bisogno. Perché se uno sta lì e ci sta a ragione e manda documenti e risponde alle mail, eccetera, per me significa che in quel momento stiamo rispondendo a un bisogno che è reale e quindi credo che sia molto importante per il loro lavoro, anche proprio come momento di confronto, momento di condivisione di debolezze, momento di aiuto nelle decisioni, che possono essere le loro ma anche di colleghi. Quindi a me sembra… insomma, mi sembra che sia, abbia un valore molto alto. Per quanto riguarda me, cioè il mio lavoro, non è un caso che io abbia detto di sì, cioè mi sia poi proposta insomma, come Centro dei servizi abbiamo fatto una ragionata e ci siamo proposti perché tutto sommato noi lavoriamo continuamente con associazioni che magari c’è l’associazione che fa la tutela del Castello di (? 35:59) però c’è anche, ci sono tante, tante associazioni sanitarie che si occupano appunto di salute, non solo quelle che si occupano di malattie e ce ne sono miliardi, ma anche quelle che si occupano di prevenzione, di promozione di una buona qualità di vita, eccetera. Allora, secondo me questa mia presenza ha un significato e ha un (? 36:28-36:33) di promozione, di… come posso dire, forse anche proprio di promozione, cioè (? 36:44-36:49) di questo luogo di confronto, è un’informazione che io ho, ed è una cosa che io posso assolutamente riportare, quando necessaria, se necessaria o anche se non necessaria; perché mi sono anche chiesta, ma questo Comitato, quando avrà passato questa fase che forse è sperimentale, da quanto ho capito, potrà essere presentato, potrebbe essere presentato al mondo delle associazioni? Perché credo che sia anche di supporto e di consolazione sapere che c’è comunque chi sta lavorando su queste tematiche, soprattutto per le sanitarie, ma poi anche dietro a tutte le altre ci sono dei cittadini, quindi cioè le questioni sanitarie ce le hanno anche loro, non è che… dal medico ci andranno anche loro immagino. Quindi secondo me è un valore importante insomma.
D:	Ecco, e rispetto magari anche a questo ultimo punto che è molto interessante, anche per uno sviluppo prossimo del CEC, però ripensando un po’ a quello che è stato fatto, ti chiedo secondo te, la tua percezione, come sono state integrate le attività svolte in questi mesi da questo Comitato nel contesto locale?
R:	La mia percezione è poco, credo che non sia tanto conosciuto questo… cioè non sia proprio conosciuto per niente il Comitato. A me è capitato di parlarne con persone che conosco, ma amici, non è che… cioè, al di là dello staff qua, perché sanno che vado lì. Mi è capitato di parlarne, mi hanno chiesto “ma che cos’è e cosa”, mi ha detto “ma veramente? Ma dai, esiste questa roba qua, e chi c’è, che cosa fanno” e così. Io peraltro penso di essere una persona che conosce tanta gente ecco, però è una realtà che non si conosce ecco, a meno che uno non abbia avuto l’esperienza di… però non è una roba che ce l’hanno tutti, questa esperienza, nel senso che… fortunatamente. Però insomma credo che sia una realtà che andrebbe, secondo me, anche per una questione appunto di aiutare la cultura di queste… e credo che sia un’esperienza che vada promossa molto di più. Come d’altra parte, ti dico la verità, anche tanti servizi interni all’azienda, sai perché quando siamo andati a fare dei lavori con le associazioni ho visto che… o anche quando ci hanno chiesto di fare il progetto… tante volte capita che ti chiedono di fare dei progetti per costruire dei servizi che in realtà poi tu vai a vedere, i servizi ci sono già, soprattutto in una realtà come quella di Reggio; la realtà reggiana insomma avrà mille pecche, però insomma non quella di non funzionare e di non aver pensato a dei servizi, cioè anzi, a volte… però mi è capitato molte volte che associazioni mi venissero a dire “facciamo un progetto perché manca un luogo dove” ti dicono, “dove devi andare quando ci sono delle dimissioni complesse…” ma poi cioè ci sono, quei servizi lì; il grosso problema è che molto spesso non si conoscono. Non credo che sia una colpa di nessuno, credo che ognuno sia molto concentrato a fare le proprie cose e le parte di promozione di questi servizi sia a volte lasciata un po’ a parte oppure viene… salta fuori perché ne hai bisogno, non sai da che parte prendere, devi avere un letto con le sponde, devi sapere che dentro all’ospedale c’è qualcuno che ti dice dove devi andare. Però succede solo quando ti capita quella roba lì e io vedo che ho dato tante volte informazioni… ma questo lo dico perché non è secondo me un tema solo legato al CEC, credo che alcuni servizi dell’azienda non siano tanto conosciuti e sono invece servizi che sono molto utili, con tutti i limiti del servizio di una grossa azienda; quindi a volte è complicato accedere, non si capisce da che parte, devi fare dei fogli, però ci sono.
D:	Ecco, rispetto a questo ultimo punto, la mia prossima domanda era proprio questa, se secondo te ci sono dei limiti, ci sono stati dei limiti organizzativi interni, esterni, qualsiasi cosa rispetto appunto a questo servizio e se ci sono stati, se vuoi raccontarmi quali sono stati secondo te.
R:	I limiti sono tutti quelli secondo me di un servizio sperimentale, cioè forse il primo limite è di essere poco conosciuto; però anche lì secondo me c’è da fare un discorso un po’ più ampio, cioè sarà anche poco conosciuto però è un servizio sperimentale. Allora, magari prima si chiude la sperimentazione, poi si fa conoscere il servizio; cioè nel senso che se noi facciamo conoscere un servizio sperimentale e non abbiamo ben chiaro se il servizio funziona, se il servizio possa essere ancora migliorabile - poi migliorabile è migliorabile tutto da sempre, non è quello - però quali sono esattamente gli obiettivi, se abbiamo centrato il bisogno, se non abbiamo… cioè da progettista io ragiono… cioè, il progetto io lo diffondo quando ho dei risultati, perché se non ho dei risultati non vado dicendo “sto progettando però (? 42:32-42:36)” il valore forse di una maggiore promozione è appunto sempre quello del peso, quello culturale, guardate che si sta riflettendo su questa roba, però capisco anche che la parte di promozione anche io la farei in un secondo momento, perché ancora esiti precisi non ce ne sono e diciamo così un’esperienza nostra, cioè nostra di Reggio, questa roba qua non… cioè si sta facendo adesso, quindi… Per il resto io degli altri limiti, a parte quelli che ho sottolineato come punti secondo me un po’ critici, come ti dicevo, potrebbe essere aumentata la presenza delle persone diciamo non cliniche e non giuriste ecco, potrebbe… ma poi basta, mi sembra che il gruppo sia tenuto molto bene, ci sia un’efficienza elevatissima, posto che io sono nel sociale, nel sociale quell’efficienza lì è difficile da trovare, cioè sì, facciamo un incontro, (? 43:45) fai quattro anni, poi dopo devi ritrovarti, poi dopo… invece mi sembra che sia un’efficienza molto alta ecco, nella tenuta del gruppo, nell’organizzazione, probabilmente perché dovete rispondere appunto a medici che hanno bisogno di averne conto e non hanno tempo da perdere. Anche gli orari, è banale, però anche la tenuta in orario, io l’apprezzo molto, perché è una roba che non è da tutti, cioè non si trova sempre. E quindi penso che il limite sia solo quello che però è molto comprensibile ed è il fatto che sia un servizio poco conosciuto, cioè si ha una… però con i se che ti dicevo.
D:	Okay. Adesso mi hai raccontato anche dei punti di forza, cioè che mi sembrano dei punti di forza, quelli che mi hai appena citato adesso; ti chiedo, questa era l’altra domanda, se ci sono degli altri punti di forza che tu hai notato? Cioè che secondo te ci sono rispetto a questo servizio?
R:	Punti di forza, ci sono… cioè ti dico, quello che ho detto mi sembra comunque già abbastanza esaustivo, cioè, provo a recuperalo; la gestione, sia da un punto di vista proprio del coordinamento che della segreteria organizzativa, eccetera, la puntualità e l’organizzazione vera e propria, si sa sempre quando ci sono delle… quando c’è la riunione, c’è il recall, eccetera; questo molto tecnico, però comunque c’è e non è una parte minima ecco, a volte è importante. Ci sono delle persone molto competenti, a volte forse non tanto in bioetica, ma forse anche su altre questioni, però insomma persone molto competenti ci sono. C’è una grande attenzione, cioè c’è davvero una grande partecipazione, anche questa non è scontata, come ti dicevo prima insomma, ci sono, vengono portati temi abbastanza concreti, cioè non ci si parla addosso, si parla su temi e questioni concrete, quindi sicuramente questo è molto utile, affronta temi che, mia percezione, in questo momento sono importantissimi, cioè dal quale non si può proprio più forse rimandare ecco, penso che sia proprio adesso il momento di… forse dovevamo cominciare prima ecco, però insomma sia proprio adesso il momento di farlo. Quindi penso che siano questi i punti di forza.
D:	Okay. Va bene, io adesso ti faccio l’ultima domanda, abbiamo concluso, che è se alla luce di quanto fatto in questi mesi, ripensando un po’ al percorso tuo, una valutazione complessiva, diciamo così, come valuti in maniera complessiva questo servizio?
R:	Cioè vuoi una valutazione, come posso dire, una sorta di voto?
D:	È fondamentalmente una domanda di chiusura. Cioè, ripensando un po’ magari anche all’evoluzione…
R:	Secondo me è un servizio… è un ottimo servizio; io proprio se devo dare proprio una… secondo me è un ottimo servizio, io lo trovo molto utile (? 47:50-47:54) sono in questa storia, quindi la parte medica, la parte del… insomma generale, la parte sanitaria, la parte culturale, la parte dei cittadini, la parte dei pazienti che… la parte dell’azienda proprio e pensando a tutti questi ruoli credo che sia un servizio molto ben pensato insomma. Ripeto, anche molto innovativo e anche simbolo secondo me di grande apertura. Quello sì, sicuramente.
D:	Va bene. Allora, io l’intervista l’ho finita e ti ringrazio davvero tantissimo del tempo che mi hai dedicato.
R:	Figurati.
D:	Il tempo è prezioso, mi dispiace che non siamo riuscite a stare nei trenta minuti, però…
R:	No, ma ho parlato tanto anch’io.
D:	Ma visto che… cioè le altre insomma, più o meno, io ho detto questo tempo qui, perché anche rispetto alle altre il tempo era un po’ questo, però veramente ti ringrazio tantissimo, perché è stata un’intervista, perché chiacchierata… veramente interessantissima. Grazie, proprio.
R:	Grazie a te.
D:	Ė stato molto… degli spunti veramente utilissimi, al di là poi di quello che sarà il risultato di questo studio, però proprio per migliorare il servizio, è stato veramente il tuo un contributo molto importante, quindi ti ringrazio davvero tanto, davvero.
R:	Bene, sono contenta.
D:	Molto.
```
