# Supplementary material for: How to implement a clinical ethics committee in an oncological research hospital: Qualitative results from a process evaluation study using normalization process theory (EVACEC)
Source: PLoS One. 2025 May 6;20(5):e0318870. doi: 10.1371/journal.pone.0318870 (PMC12054913; doi:10.1371/journal.pone.0318870)
Supplement: Attachment — Submitted filename: Response to reviewers.docx [file pone.0318870.s007.docx]

# **Response to reviewers**

**REVIEWER N#1**

Thank you for your kind evaluation of our work and your precise comments. We really appreciated them, and we believe the revision you requested significantly improved our manuscript.

Thank you for your comment on whether the CEC was only for oncology; it provided us with the opportunity to elucidate a critical aspect of this service. We agreed that the paragraphs involving oncology were confusing. From their writing, it could be deduced that the CEC was dedicated only to oncologists and not–as it was instead–a service available for all healthcare professionals, no matter their clinical area.

Your comment was also an occasion to rethink the link with oncology throughout the manuscript. We have made multiple significant changes to the text to clarify this point and reformulated these concepts within the text. Also, we added a mention to the fact that we didn’t receive any request from oncologists in the limitation. Please, see: *Background*, lines 95-97; *Strengths and limitations*; lines 486-493).

Regarding your revision/suggestions, we made the following changes:

**Title, grammar, punctuation:**

- we removed the question mark;
- we revised the text, corrected all misspelling and punctuation errors, and fixed the formatting of the file as suggested;
- we welcomed all your suggestions on grammar and made the changes where applicable.

**Abstract**:

- we replaced the term “stakeholders” with “LHA directors” (line 24).

**Background**:

- We followed your instructions and revised the background accordingly: in fact, we first addressed CESS in general and then clarified that our study concerns a specific form of CESS (our CEC) (lines 39-61).

**Methods**:

- A pilot study was conducted with two participants. While no significant changes were necessary, adjustments and minor modifications were made based on the pilot responses (lines 137-138)
- we agreed it would benefit the reader to provide a brief description of the 4 NPT concepts within the main document and added it (lines 112-118)
- We revised the Methods section to include all the details of domains 1 and 2 of the COREQ to fill in all the missing information (lines 136-144). We did not write a separate “reflexivity” paragraph to avoid making the text less fluid. However, we believe the role of all team researchers and their relationship with the CEC and its members are now clear. We discuss potential biases in the *Strengths and limitations* section (lines 479-485).
- Yes, we developed two different topic-interviews guidelines: one for LDs and one for CMs. NPT concepts informed both and both are accessible in the *Supplementary Materials*.
- Thank you for suggesting combining some key areas together. They were indeed repetitive, so we revised them accordingly and synthesized them (lines 120-125).
- We have now explained in the manuscript how we found eligible participants and how we obtained consent (lines 122-125 and line 129)
- Since all the people involved in the study are native Italian speakers, all the interviews were conducted in Italian and were not translated. Also, all interviews were analyzed directly in Italian to respect the original meanings intended by the participants underlying their statements and to give voice to the semantic complexity of what emerged from the interviews. Only the quotations included in the manuscript were translated (translated by us) (lines 128-130)
- In the *Data Analysis* paragraph we referred to an excel file reporting all the data underlying the findings described in the manuscript, along with the codes from the qualitative analysis we conducted. That file was indeed missing in the first submission, we apologize. We have now uploaded it as *Supplementary material* (line 140).
- We added a phrase explaining how we managed conflicts and reached consensus, which was indeed missing (lines 145-147). Also, as specified in the text, the research team included a methodologist who helped structure the methodological section and reviewed the manuscript at each step.

**Results:**

- Thank you for commenting on Figure 1. Your comment helped us to better clarify an important aspect of our results. We agree with you, and we have amended the Figure accordingly.
- Since the president of the CEC is among the authors of the paper, we deemed it necessary to identify the president for transparency purposes regarding potential conflicts of interest or bias.
- We agree with the reviewer, and we confirm that the sentence in line 274 (now lines 282-285) is still part of our results. We modified the text to clear the sentence.

**Discussion:**

- We appreciate your compliment on how we addressed the humanization of care, which is a very crucial topic for us as well. Thank you very much. The humanization of care is a recurring theme in the bioethics literature; still, its explicit association with ethics is apparently only found in articles by Italian researchers (ref. 41 & 42 of the manuscript). We found no other records connecting the two concepts, and we hypothesize that this connection has something to do with the Italian cultural context. But it is, indeed, a hypothesis and we modified the text accordingly (line 397).
- We agree that our *Discussion* was too long, and that some concepts did not relate to the results section. We removed several phrases and paragraphs (see the track changes version).
- We agree that our use of CEC and CESS could have been clearer and revised the text to clarify it. We had to keep a reference to both CESS and CEC, as we refer multiple times to two main cross-sectional research lines (CESSs outcomes and CESSs implementation) but conducted a study on a CEC, a specific form of CESS. So, depending on the concept we were explaining, we had to refer generally to CESS or specifically to the object of our study. However, we believe the two concepts are more straightforward now and we better described which one we discussed in each paragraph (lines 378-382).
- We added comparisons to our results, as suggested (lines 409-412 and lines 422-424).

**Practical implications:**

- We greatly appreciate your comment on this section. In **Table 4** caption, we have now clarified that its content was developed through a combination of our results and personal suggestions (see Table 4 caption).

**REVIEWER N#2**

Thank you very much for appreciating our work. Your questions allowed us to reflect on crucial aspects of our research and revise the manuscript accordingly.

Regarding how ethical issues were managed before the CEC: we clarified that the Bioethics Unit operated as the only ethics support service until the CEC was implemented and dealt with every request for ethics support/training (lines 75-76). Also, the CEC has been developed because of the experience gained by the Bioethics Unit ethics support services (lines 79-81)

As for the diversity of the CEC members, we did include one citizen’s representative as member, who was the Head of the Territorial Planning and Organization Area of CSV Emilia (Volunteer Service Center - CSV Emilia brings together volunteer and patient associations active in the provincial territory). However, it was explicitly stated in the CEC regulations that specific external experts—who can meaningfully represent the patient’s cultural and/or religious perspectives—could be invited by the CEC president during consultations, if necessary.  We did not include those potential members in the sample, as they participate in the CEC discussion only occasionally, and do not offer a representative perspective on implementation (*Methods*, lines 122-125, *Strengths and limitations*, lines 494-496).

We concur that, due to the short time period under study, our findings can address the limitations of a CEC rather than establish a long-term impact; however, we believe our results are still valuable and useful for professionals who aim at performing similar evaluations.

Due to the CEC transition into a regional service, we cannot conduct a long-term or even longitudinal analysis of it, which would have shed more light on the implementation issue.

However, since the regional CEC (named COREC) was developed and implemented based on previous experience, it would be valuable to conduct an effectiveness evaluation of the COREC in the future.

While implemented within an oncological research hospital, the CEC was intended for healthcare professionals from all specialties within the institution, not only those working in cancer care (lines 95-97). Based on our results, we do not believe that every specialty should have its own specific CEC; rather, we believe that an effective CEC is well integrated into its context, and therefore, we think that each institution/hospital should have its own CEC for this to happen.

However, the CEC we studied has now become a regional committee (called COREC) for political reasons. Further research is needed to understand how a regional committee works and what is the best model.

We agree that patients' opinions (along with those of their loved ones) are pivotal in determining any changes the CEC would provide in healthcare services received. However, in this paper, we focused our evaluation on the implementation process. Our aim was to identify the components that make the CEC normalized and integrated in clinical practice for its users (healthcare professionals, HPs). We will include the perspectives of CEC users (HPs who requested an ethics consultation) in a dedicated paper.

We added some information about the ethics competence/training of the CEC members (lines 155-159).

**REVIEWER #3**

Thank you very much for your valuable contribution. The numerous and significant comments you have given on our work and your positive feedback have been a great source of encouragement for our team and motivate to continue our research with dedication.

**REVIEWER #4**

Thank you for your interest in our work and your kind comments. We appreciate your evaluation of our manuscript. We welcomed your suggestions and, accordingly, made a few changes to the *Discussion* section. We fixed all typos and misspelling errors.

We had already addressed the potential generalization of our *Results*; in fact, Table 4 (§ “Practical implications”) was built with that aim in mind. However, we had not explicitly mentioned potential generalization nor was the distinction between results and original proposals clear, so we added a short paragraph on the possible generalization of our results (lines 466-468).

**REVIEWER #5**

Thank you for your comments. We agree our manuscript was too long. We significantly shortened the manuscript whenever possible and removed all the content not directly related to the described results from the *Discussion.* We fixed all typos, tried to keep abbreviations to a minimum and spelled them out in full whenever possible (e.g. Bioethics Unit instead of BU). We revised the *Methods* section for clarity, as suggested. Thank you for pointing out that our language was restrictive. We made efforts to simplify it and believe our manuscript is now more readily understandable.
